# Signature of miRNAs derived from the circulating exosomes of patients with amyotrophic lateral sclerosis

**DOI:** 10.3389/fnagi.2023.1106497

**Published:** 2023-02-10

**Authors:** Yang-Fan Cheng, Xiao-Jing Gu, Tian-Mi Yang, Qian-Qian Wei, Bei Cao, Yang Zhang, Hui-Fang Shang, Yong-Ping Chen

**Affiliations:** ^1^Department of Neurology, Laboratory of Neurodegenerative Disorders, Rare Disease Center, West China Hospital, Sichuan University, Chengdu, China; ^2^Laboratory of Neurodegenerative Disorders, National Clinical Research Center for Geriatric, West China Hospital, Sichuan University, Chengdu, China; ^3^West China School of Medicine, West China Hospital, Sichuan University, Chengdu, China

**Keywords:** amyotrophic lateral sclerosis, microRNAs, exosomes, gene mutation, diagnostic model

## Abstract

**Background:**

Amyotrophic lateral sclerosis (ALS) is a progressive, fatal neurodegenerative disorder (NDS) with unclear pathophysiology and few therapeutic options. Mutations in *SOD1* and *C9orf72* are the most common in Asian and Caucasian patients with ALS, respectively. Aberrant (microRNAs) miRNAs found in patients with gene-mutated ALS may be involved in the pathogenesis of gene-specific ALS and sporadic ALS (SALS). The aim of this study was to screen for differentially expressed miRNAs from exosomes in patients with ALS and healthy controls (HCs) and to construct a miRNA-based diagnostic model to classify patients and HCs.

**Methods:**

We compared circulating exosome-derived miRNAs of patients with ALS and HCs using the following two cohorts: a discovery cohort (three patients with *SOD1*-mutated ALS, three patients with *C9orf72*-mutated ALS, and three HCs) analyzed by microarray and a validation cohort (16 patients with gene-mutated ALS, 65 patients with SALS, and 61 HCs) confirmed by RT-qPCR. The support vector machine (SVM) model was used to help diagnose ALS using five differentially expressed miRNAs between SALS and HCs.

**Results:**

A total of 64 differentially expressed miRNAs in patients with *SOD1*-mutated ALS and 128 differentially expressed miRNAs in patients with *C9orf72*-mutated ALS were obtained by microarray compared to HCs. Of these, 11 overlapping dysregulated miRNAs were identified in both groups. Among the 14 top-hit candidate miRNAs validated by RT-qPCR, hsa-miR-34a-3p was specifically downregulated in patients with *SOD1*-mutated ALS, while hsa-miR-1306-3p was downregulated in ALS patients with both *SOD1* and *C9orf72* mutations. In addition, hsa-miR-199a-3p and hsa-miR-30b-5p were upregulated significantly in patients with SALS, while hsa-miR-501-3p, hsa-miR-103a-2-5p, and hsa-miR-181d-5p had a trend to be upregulated. The SVM diagnostic model used five miRNAs as features to distinguish ALS from HCs in our cohort with an area under receiver operating characteristic curve (AUC) of 0.80.

**Conclusion:**

Our study identified aberrant miRNAs from exosomes of SALS and ALS patients with *SOD1*/*C9orf72* mutations and provided additional evidence that aberrant miRNAs were involved in the pathogenesis of ALS regardless of the presence or absence of the gene mutation. The machine learning algorithm had high accuracy in predicting the diagnosis of ALS, shedding light on the foundation for the clinical application of blood tests in the diagnosis of ALS, and revealing the pathological mechanisms of the disease.

## Introduction

Amyotrophic lateral sclerosis (ALS) is a fatal, neurodegenerative disease (NDS) characterized by selective loss of upper and lower motor neurons (Cleveland and Rothstein, 2001). This degeneration of neurons manifests clinically with insidious focal weakness that spreads to most skeletal muscles, eventually to the diaphragm, leading to death in most of those diagnosed 2–5 years after the onset of symptoms due to respiratory dysfunction (Kiernan et al., 2011). The cause of most patients with ALS is unknown, although 5–10% of them have familial forms and some of them are associated with mutated genes, such as *C9orf72*, *SOD1*, *TARDBP*, and *FUS* (Brown and Al-Chalabi, 2017). Pathologically, a specific mechanism for neurodegeneration in patients with gene mutation might differ from that in those with sporadic ALS (SALS) mainly in the initial stage, but the shared pathological alteration might also be seen, especially in the advanced stage. Clinically, the dilemma for clinical doctors is the delay from the onset to diagnosis owing to the huge heterogeneous clinical presentation (Paganoni et al., 2014). Thus, identification of clinically and mechanically relevant different or shared biomarkers, hidden in patients with or without gene mutation, will benefit unearthing the pathogenesis of ALS, and early and efficiently diagnosing the various forms of ALS.

Exosomes, also known as intraluminal vesicles, are a subpopulation of extracellular vesicles with a 30–150-nm diameter derived from multi-vesicular bodies that transmit cellular molecular constituents to promote intercellular communication (Cheng et al., 2014). It could be secreted by nearly all types of cells that cross the blood–brain barrier and enter the circulatory system. Transactive response DNA-binding protein 43 kD (*TDP-43*), a major disease-associated component in the brain of patients with ALS, was reported in the presence of cellular exosomes (Feiler et al., 2015). Basso et al. (2013) found that superoxide dismutase 1, encoded by *SOD1*, which is the most common causative gene in the Chinese patients (Chen et al., 2021), could be transferred from astrocyte-derived exosomes to spinal neurons, inducing selective motor neuron death (Basso et al., 2013). Moreover, Westergard et al. (2016) found evidence for cell-to-cell spreading of dipeptide repeat proteins (DRPs) produced by pathologic *C9orf72* hexanucleotide repeat expansions (HREs) *via* exosome-dependent in the spinal motor neurons derived from induced pluripotent stem cells from patients with ALS. These studies supported a prion-like cell-to-cell diffusion mechanism in ALS that was possibly mediated by exosomes.

More importantly, non-coding RNAs (ncRNAs) are among the most abundant contents in exosomes, which exist stably and can be tested easily (Valadi et al., 2007; Tang et al., 2021). Among them, microRNAs (miRNAs) are small single-stranded RNA genes (18–25 nucleotides) that are involved in the host cells by targeting mRNAs for cleavage or post-transcriptional gene regulation (Bartel, 2004). Many studies found that miRNA participated in nervous system development (Åkerblom et al., 2012; Zhu et al., 2013) and dysregulation of miRNA had been shown to influence the pathogenesis of neurological diseases (Feng et al., 2018; Pan et al., 2021). Our previous studies have also reported aberrant miRNAs in leukocytes from SALS (Chen et al., 2016), among which miR-183-5p (Li C. et al., 2020) and miR-193b-3p (Li et al., 2017) were involved in the neurodegeneration by functional investigations. Although the identified miRNAs in leukocytes or from plasma/serum practical significance for ALS diagnosis, it is difficult to explain the degeneration of the selected motor neuron due to the lack of the targeted from exosomes membrane receptors (Gonda et al., 2019). Thus, whether miRNAs derived from exosomes of patients with ALS are aberrant and how they might participate in the pathogenesis of ALS is yet unclear.

In this study, we sought to: (1) identify aberrant miRNAs from circulating exosomes of *SOD1*-ALS, *C9orf72*-ALS, and SALS by microarray screening and RT-qPCR validation; (2) construct a support vector machine (SVM) model by using differentially expressed miRNAs between SALS and HCs to help diagnose ALS; and (3) perform functional analysis of predicted gene targets of aberrant miRNAs to find potential pathophysiological pathways.

## Materials and methods

### Subject

The recruitment of patients and healthy controls (HCs) was conducted at the Department of Neurology, West China Hospital of Sichuan University, from August 2017 to August 2019. All the patients with ALS were diagnosed based on the revised El Escorial criteria for definite or probable ALS (Brooks et al., 2000) by board-certified neurologists. HCs were typically spouses and friends. They were also evaluated by experienced neurologists, and routine blood tests were carried out to eliminate neurological conditions. Demographic characteristics, clinical information, and blood samples were collected from all participants at baseline. Disease severity was assessed with the Revised ALS Functional Rating Scale (ALSFRS-R), and disease progression was calculated as (48-ALSFRSR)/disease duration (months) (Kimura et al., 2006). Besides, all the patients with ALS received a genetic test (Chen et al., 2021). Written and signed informed consent was obtained from all the participants. Approval was obtained from the Ethics Committee of the West China Hospital of Sichuan University (approval number 2016-097).

### Sample collection, study design, and microarray

Whole human peripheral blood, collected in sterile vacutainer tubes containing the anticoagulant ethylenediaminetetraacetic acid (EDTA), was centrifuged at 2,000 rpm for 10 min at 4°C within 2 h after collection. Plasma fractions were subsequently collected, aliquoted, and stored at −80°C. To avoid the interference of hemolysis, plasma samples were examined for hemolysis based on a two-step method. First, absorbance was measured at 414 nm by using a spectrophotometer (Thermo Fisher Scientific Nanodrop), and samples with results lower than 0.3 were selected for the next step. Second, plasma samples were further tested for expression levels of two miRNAs, namely, miR-451 and miR-23a. A ratio between two miRNAs calculated as delta Ct (miRNA-23a-3p–miRNA-451) was used as a hemolysis indicator. A ratio of more than seven indicates a high risk of hemolysis. For both absorbance_414*nm*_ < 0.3 and delta Ct < 7, the plasma sample could be extracted from the exosomes (Blondal et al., 2013).

**FIGURE 1:**
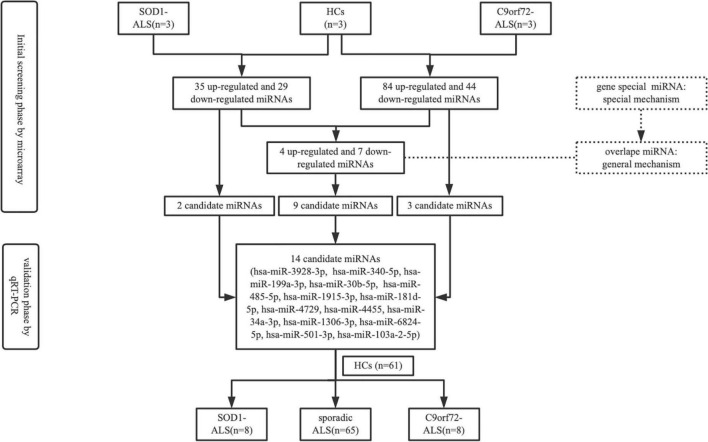
Schematic diagram illustrating the study design.

The schematic diagram of the study design is shown in Figure 1. In the initial screening phase, we selected three patients with *SOD1*-mutated ALS, three patients with C*9orf72*-mutated ALS (HREs > 30 in *C9orf72*, Dekker et al., 2016; Chen et al., 2021), and three HCs. Although microarray was based on a small number of samples, it also allowed us to explore the miRNAs of patients with ALS, compare them to HCs, then select an interesting subset of miRNAs for further study, and perform real-time PCR validation. Total RNA extracted from exosomes originating from plasma (1 ml) was labeled with the Flashtag™ Biotin HSR RNA labeling kit (Thermo Fisher Scientific) following the manufacturer’s instructions. Labeled RNA was hybridized at 48°C for 16 h at 60 rpm on an Affymetrix GeneChip™ human miRNA 4.0 array (Thermo Fisher Scientific), which contains 2,578 human mature miRNA probe sets and 2,025 human pre-miRNA (stem-loop) probe sets. GeneChips were scanned using the Affymetrix GeneChip scanner G3000 7G with the standard setting to capture signal intensities for the miRNAs. The raw intensity data were imported into the Affymetrix Expression Console software (version 1.4.1.46) for signal pre-processing, including background correction utilizing the robust multi-array average algorithm, median polish summarization from probe-to-probe set level of signal values, and the quantile method to normalize across multiple arrays. A detection call on the strength of miRNA signal was made using the Affymetrix “Detection Above Background” algorithm, which generates a *p*-value for the signal above background probability (Zheng et al., 2021). The normalized log_2_ intensity values were analyzed for differential expression between different time points using the R software “limma” package, which uses a moderated *t*-test with linear modeling and empirical Bayes statistics (Ritchie et al., 2015). The significance for differential expression was set at (absolute log_2_ fold-change) ≥ 1.2 and adjusted *p* < 0.05. In the validation phase, the selected miRNAs were further validated with 8 patients with *SOD1*-mutated ALS, 8 patients with *C9orf72*-mutated ALS, and 61 HCs. Meanwhile, 65 patients with SALS were also recruited to validate whether miRNAs, which were differentially expressed between ALS patients with *SOD1*/*C9orf72* mutations and HCs, had alteration in SALS. The selected differential expression of miRNA was based on the following conditions: (1) highly expressed in brain tissue relative to other tissues; (2) predicted target genes of miRNA included *TARDBP*, *SOD1*, *C9orf72*, *FUS*, *SQSTM1* (p62), *UBQLN2*, or neurofilament protein; (3) combined with bioinformatics functional prediction, online miRNA database, and pathophysiological characteristics of ALS; and (4) differentially expressed in the ALS group with absolute log_2_ fold-change ≥ 1.2 and adjusted *p*-value of < 0.05.

### miRNA isolation from exosomes, reverse transcription to cDNA, and quantitative RT-qPCR validation

Exosomes from frozen plasma (1 ml) were extracted by the exoRNeasy Midi Kit (Qiagen) according to the manufacturer’s instructions after thawing on ice and centrifuging at 13,000 rpm for 1 min to get rid of cellular debris. *Caenorhabditis elegans* miR-39 (cel-miR-39) synthetic oligonucleotide RNA (miRNeasy Serum/Plasma Spike-In Control) was used as an exogenous control (Aday et al., 2021), which was added to the plasma after the addition of a denaturing solution. The concentration of miRNA was measured using NanoDrop One (Thermo Fisher Scientific). For cDNA synthesis, 10 ng of total RNA was reverse-calculated from the concentration and was reverse-transcribed in a 10-μl reaction using the miRCURY LNA miRNA PCR Starter Kit (Qiagen). A miRCURY LNA SYBR^®^ Green PCR Kit (Qiagen) and miScript Primer Assays (Qiagen) were used on the Applied Biosystems^®^ StepOnePlus RT-qPCR system to quantify the miRNAs in exosomes. According to the abovementioned selected condition, 14 candidate miRNAs (Figure 1), including two *SOD1*-ALS exclusively (hsa-miR-3928-3p and hsa-miR-340-5p), three *C9Orf72*-ALS exclusively (hsa-miR-199a-3p, hsa-miR-30b-5p, and hsa-miR-485-5p), and nine overlapped in *SOD1* and *C9orf72*-ALS (hsa-miR-1915-3p, hsa-miR-181d-5p, hsa-miR-4729, hsa-miR-4455, hsa-miR-34a-3p, hsa-miR-1306-3p, hsa-miR-6824-5p, hsa-miR-501-3p, and hsa-miR-103a-2-5p), were amplificated using Qiagen miScript primers. Hsa-miR-16-5p was used as an endogenous control because it showed minimum variance and approximation value between patients and HCs according to miRNA microarray analysis and previous studies (Lange et al., 2017; Bohatá et al., 2021). Run in triplicate and comparative quantification was used to determine the relative quantities of miRNA from hsa-miR-16-5p and cel-miR-39-3p.

### Prediction of miRNA target genes, functional analysis, and preliminary validation

The prediction of miRNA target genes/sites was carried out by three different miRNA online databases, including TargetScanHuman 7.2^1^ (Agarwal et al., 2015), Diana Tools^2^, (Vlachos et al., 2015), and miRND^3^ (Chen and Wang, 2020). The expression of miRNA in human tissue refers to the YM500v2 database^4^ (Cheng W. C. et al., 2015). Venn diagrams were calculated and drawn by an online website^5^. Gene ontology (GO) analyses, which consisted of cellular components, molecular function, biological process, and Kyoto Encyclopedia of Genes and Genomes (KEGG) analyses, were conducted by DAVID Bioinformatics Resources version 6.8^6^ (Huang da et al., 2009a,b). Dot plots were drawn by the online tool bioinformatics^7^. A double fluorescent enzyme reporter assay was used to verify the binding between miRNA and the 3′UTR of the targeted gene.

### Statistical analysis and modeling

Statistical analysis for RT-qPCR was conducted on 2^–ΔΔ*Ct*^ values (ΔCt = Ct_miRNA_−0.5 * [Ct_miR–16_ + Ct_cel–miR–39_]) for each sample with GraphPad Prism version 6.0 and SPSS version 24. The continuous variable was presented as mean ± standard error of the mean (SEM). Outliers were identified using the ROUT method in GraphPad Prism version 6.0 (Q1/41%). The distribution of the data was determined using the D’Agostino–Pearson test and the Kolmogorov–Smirnov test. To compare the two groups of continuous variables, a two-sample *t*-test or Welch’s *t*-test was used when the data were in accordance with normal distribution, while the Mann–Whitney *U*-test was used. Correlations were analyzed using Pearson’s and Spearman’s rank correlation tests if the data were parametric and non-parametric, respectively. All statistics were two-tailed, and significance was set at *p* < 0.05.

The prediction model of ALS diagnosis was carried out by R environment (version 3.6.3). Standardized data were established for the machine learning-based SVM model by using the R package e1071. Patients were classified into a training cohort and a validation cohort in a ratio of 4:1. Approximately, 20% of the datasets were used to validate the predictive model created by the other 80% of the datasets. To obtain the average diagnostic performance, 5-fold cross-validation was used. Then, we created a confusion matrix that showed the results of the prediction of models to get the mean sensitivity, specificity, accuracy, and areas under the receiver operating characteristic curve (AUCs) of each model, which could be used to evaluate the efficacy of the diagnostic model.

## Results

### Clinical characteristics

A total of 65 patients with SALS, 22 ALS patients with *SOD1/C9orf72* mutations, and 64 HCs participated in this study, of which 3 patients with *SOD1*-mutated ALS, 3 patients with *C9orf72*-mutated ALS, and 3 HCs were in the initial screening phase and 8 patients with *SOD1*-mutated ALS, 8 patients with *C9orf72*-mutated ALS, 65 patients with SALS, and 61 HCs were in the validation stage. Demographic characteristics and clinical information are shown in Table 1. Genetic data for ALS patients with *SOD1/C9orf72* mutations are detailed in Supplementary Table 1.

**TABLE 1:** The demographic characteristics and clinical information of participants in this study.

Variables	Initial screening group			Validation group			
	*SOD1*-ALS	*C9orf72*-ALS	HCs	SALS	*SOD1*-ALS	*C9orf72*-ALS	HCs
Num.	3	3	3	65	8	8	61
Male (%)	1 (33.3%)	2 (66.7%)	1 (33.3%)	39 (60.0%)	2 (25.0%)	5 (62.5%)	34 (55.7)
Mean age (SD), years	51.0 (4.6)	51.3 (4.1)	51.6 (3.5)	51.3 (8.5)	51.8 (8.2)	59.8 (7.5)	51.4 (7.3)
Mean disease duration (SD), months	19.8 (14.2)	11.2 (7.3)	/	11.9 (6.8)	17.8 (13.9)	12.5 (5.3)	/
Mean age at onset (SD), years	49.3 (4.6)	50.4 (4.0)	/	50.3 (5.8)	50.3 (7.9)	58.8 (7.4)	/
ALSFRS-R	38.7 (4.8)	31 (7.1)	/	38.6 (7.1)	38.9 (7.5)	42.6 (3.6)	/

ALS, amyotrophic lateral sclerosis; ALSFRS-R, ALS functional rating scale revised; *C9orf72*-fALS, C9orf72-mutant familial ALS; HC, healthy control; SALS, sporadic ALS; SD, standard deviation; *SOD1*-fALS, *SOD1*-mutant familial ALS. ALSFRS-R score at the time of sample collection.

In the validation group, gender and age were not statistically significant in patients with SALS and *SOD1*-mutated ALS compared to HCs. Regarding disease duration, no statistical significance was shown in patients with SALS, *SOD1*-mutated ALS, and *C9orf72*-mutated ALS. However, the mean age of onset was much higher in the *C9orf72*-mutated group than in the *SOD1*-mutated group (*p* = 0.027).

### Differential expression of miRNAs from exosomes by using microarray

**FIGURE 2:**
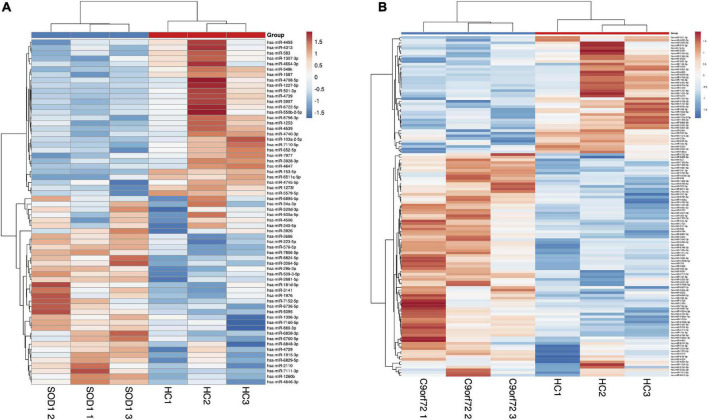
The heatmap of differentially expressed miRNAs by means of microarray. **(A)** The comparison of patients with *SOD1*-mutated amyotrophic lateral sclerosis (ALS) and healthy controls; **(B)** The comparison of patients with *C9orf72*-mutated ALS and healthy controls. Compared with HCs, we found 64 miRNAs differentially expressed in *SOD1*-mutant ALS where 35 of them were upregulated, and 29 of them were downregulated, and 128 miRNAs dysregulated including 84 upregulation and 44 downregulation in patients with *C9orf72*-mutated ALS.

Exosomes were isolated from hemolysis-free plasma and characterized by transmission electron microscopy (TEM), Nanosight Tracking Analysis (NTA), and expression of the exosome surface markers (Supplementary Figure 1). Compared to HCs, we found 64 miRNAs differentially expressed in *SOD1*-mutated ALS, of which 35 were upregulated and 29 were downregulated. Meanwhile, 128 miRNAs were dysregulated in patients with *C9orf72*-mutated ALS, including 84 upregulated and 44 downregulated miRNAs, based on our cutoff values for differential expression (fold change ≥ 1.2; adjusted *p*-value < 0.05) (Figures 2A, B). Interestingly, 11 miRNAs, including 4 upregulated and 7 downregulated miRNAs, were differentially expressed in patients with *SOD1*-mutated ALS and *C9orf72*-mutated ALS with overlapping (Supplementary Figure 2 and Supplementary Table 2), which might indicate a common pathology involved in ALS.

Among the differentially expressed miRNAs screened by microarray, the online miRNA databases were used to predict the potential target genes and their binding sites. We focused more on those miRNAs that were predicted to bind to ALS-related genes, including *TARDBP*, *SOD1*, *C9orf72*, *FUS*, *SQSTM1 (p62)*, and *UBQLN2*. For example, hsa-miR-340-5p (*SOD1*-ALS exclusively) was combined with UTR of *SOD1* potentially as a 7mer-A1 type [an exact match to positions 2–7 of the mature miRNA (the seed) followed by an “A”], while hsa-miR-199a-3p and hsa-miR-30b-5p (two of them were *C9orf72*-ALS exclusively) were predicted to combine with the UTR of *C9orf72* as 7mer-m8 type (an exact match to positions 2–8 of the mature miRNA). In addition, hsa-miR-181d-5p (overlapped in *SOD1*&*C9orf72* groups) was also predicted to combine with the UTR of *TARDBP* as 7mer-m8 type. More details can be seen in Supplementary Table 3. According to the criteria of candidate miRNAs selection mentioned in the Section ‘‘Materials and methods,’’ a total of 14 miRNAs, including two *SOD1*-ALS exclusively (hsa-miR-3928-3p and hsa-miR-340-5p), three *C9orf72*-ALS exclusively (hsa-miR-199a-3p, hsa-miR-30b-5p, and hsa-miR-485-5p), and nine overlapped both in *SOD1* and *C9orf72*-ALS (hsa-miR-1915-3p, hsa-miR-181d-5p, hsa-miR-4729, hsa-miR-4455, hsa-miR-34a-3p, hsa-miR-1306-3p, hsa-miR-6824-5p, hsa-miR-501-3p, and hsa-miR-103a-2-5p), were found to be highly expressed in the brain tissue^8^ and were selected to be validated by RT-qPCR.

### Differential expression of miRNAs from exosomes validated by RT-qPCR

To confirm the above-dysregulated candidate miRNAs, 8 patients with *SOD1*-mutated ALS, 8 patients with *C9orf72*-mutated ALS, and 61 HCs were recruited for validation by RT-qPCR. In addition, 65 patients with SALS were also included to verify whether these candidate miRNAs were also differentially expressed, particularly miRNAs that overlapped between *SOD1*-ALS and *C9orf72*-ALS. Only the samples that passed the hemolysis test were used (refer to Supplementary Table 4). The external and internal controls had a good stability and quality control (the mean Ct and SD of hsa-miR-16-5p: 19.85 ± 1.53; cel-miR-39-3p: 14.90 ± 1.33) (refer to Supplementary Table 4).

**FIGURE 3:**
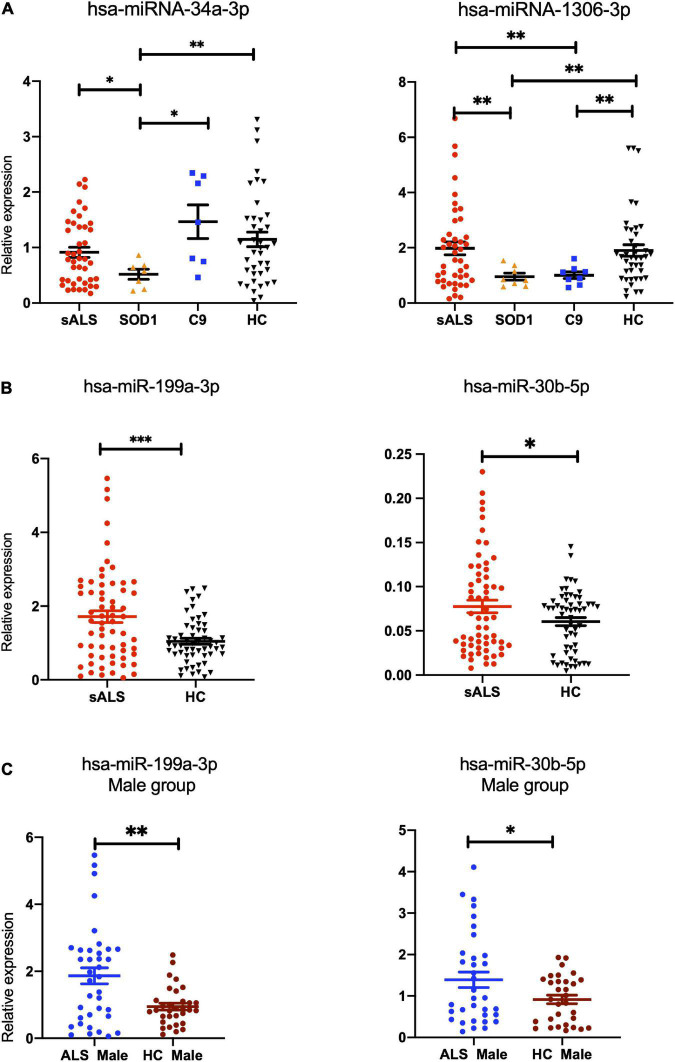
The differentially expressed miRNAs found in patients with gene-mutant and sporadic ALS compared with healthy controls validated by RT-qPCR. **(A)** hsa-miR-34a-3p was found to be specifically downregulated in patients with *SOD1*-ALS compared to patients with *C9orf72*-ALS (*p* = 0.0175), sporadic amyotrophic lateral sclerosis (SALS) (*p* = 0.032), and HCs (*p* = 0.0022), while no statistical significance was found between SALS and HCs (*p* = 0.151). Interestingly, hsa-miR-1306-3p showed significant downregulation in ALS patients with *SOD1* and *C9orf72* gene mutations, compared to SALS and HCs (SALS vs. *SOD1*-ALS: *p* = 0.0021, SALS vs. *C9orf72*-ALS: *p* = 0.0032, *SOD1*-ALS vs. HCs: *p* = 0.002, *C9orf72*-ALS vs. HCs: *p* = 0.0032) and no statistical significance was found between SALS and HCs (*p* = 0.807). **(B)** Compared to HC, hsa-miR-199a-3p (*p* = 0.0003) and hsa-miR-30b-5p (*p* = 0.0474) were found to be upregulated in patients with SALS (*p* = 0.0474). **(C)** In the subgroup analysis on gender, compared to male HCs, hsa-miR-199a-3p (*p* = 0.0014) and hsa-miR-30b-5p (*p* = 0.0309) were confirmed to be upregulated significantly in the male SALS group. HCs, health controls. ^***^p < 0.001, ^**^p < 0.01, *p < 0.05.

Hsa-miR-34a-3p was found to be specifically downregulated in patients with *SOD1*-ALS compared to patients with *C9orf72*-ALS (*p* = 0.0175), patients with SALS (*p* = 0.032), and HCs (*p* = 0.0022), while no statistical significance was found between SALS and HCs (*p* = 0.151). Interestingly, hsa-miR-1306-3p showed significant downregulation in patients with *SOD1* and *C9orf72* gene mutations, compared to patients with SALS and HCs (SALS vs. *SOD1*-ALS: *p* = 0.0021, SALS vs. *C9orf72*-ALS: *p* = 0.0032, *SOD1*-ALS vs. HCs: *p* = 0.002, *C9orf72*-ALS vs. HCs: *p* = 0.0032) and no statistical significance was found between patients with SALS and HCs (*p* = 0.807) (Figure 3A). Compared to HCs, hsa-miR-199a-3p (*p* = 0.0003) and hsa-miR-30b-5p (*p* = 0.0474) were significantly upregulated in patients with SALS (Figure 3B). Interestingly, these differences were found mainly in the male subgroup (male group: hsa-miR-199a-3p: *p* = 0.0014; hsa-miRNA-30b-5p: *p* = 0.0309) but not in the female subgroup (female group: hsa-miR-199a-3p: *p* = 0.1639; hsa-miRNA-30b-5p: *p* = 0.7760; Figure 3C). In addition, hsa-miR-501-3p (*p* = 0.0533), hsa-miR-103a-2-5p (*p* = 0.0603), and hsa-miR-181d-5p (*p* = 0.0782) were also potentially upregulated (0.05 < *p* < 0.10) in patients with SALS (Supplementary Figure 3). The comparison of these 14 candidate miRNAs by microarray and RT-qPCR can be found in Supplementary Table 5.

### The correlation between miRNAs and clinical phenotype

**FIGURE 4:**
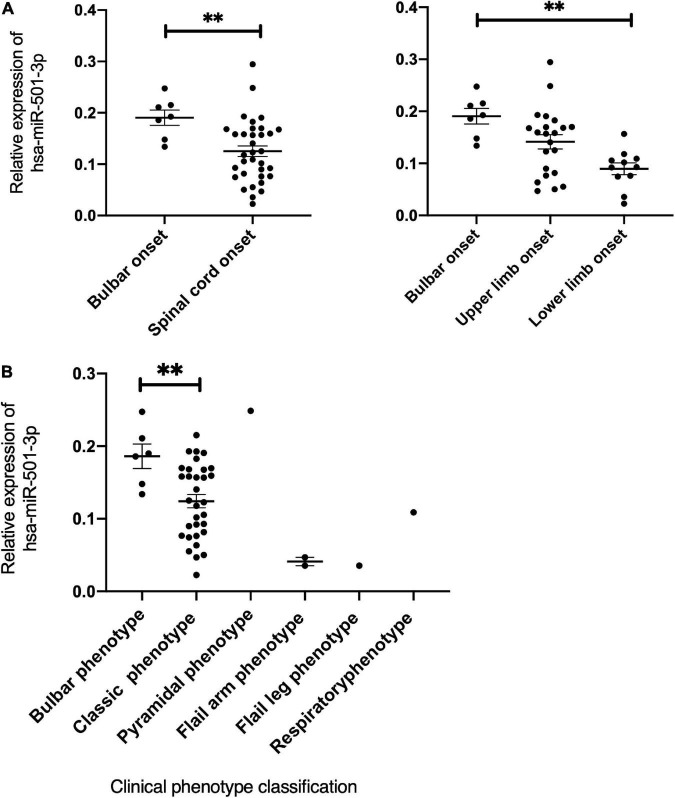
The expression of has-miR-501-3p and the correlation with clinical characteristics in patients with ALS. **(A)** The relationship of the relative expression of hsa-miR-501-3p and the onset site. Hsa-miR-501-3p was found to be specifically increased in patients whose initial symptoms presenting at bulbar-onset (*p* = 0.0098). Compared to lower limb onset, hsa-miR-501-3p was increased significantly in bulbar-onset of patients with ALS (*p* = 0.0027) and there was no significance between upper limb onset and lower limb onset groups. **(B)** The relationship of the relative expression of hsa-miR-501-3p and the clinical phenotype classification. ^**^p < 0.01.

Then, we conducted a correlation analysis of miRNA expression with the demographic and clinical characteristics of patients with ALS. We found that hsa-miR-501-3p was increased in patients with initial symptoms presenting at bulbar-onset (*p* = 0.0098) compared to the spinal cord-onset group. In more detail, compared to the lower limb-onset group, hsa-miR-501-3p was significantly increased in bulbar-onset patients with ALS (*p* = 0.0027), with no significance between the upper and lower limb-onset groups (Figure 4A). Interestingly, if we grouped patients with ALS according to the new clinical phenotype classification (Chiò et al., 2011), the expression of hsa-miR-501-3p was also increased in the bulbar phenotype in comparison with the classic (Charcot’s) phenotype (*p* = 0.0178, Figure 4B). Furthermore, we found a positive correlation between the expression of hsa-miR-501-3p and the age of patients with ALS at the last assessment (*r* = 0.3245, *p* = 0.0384, simple linear regression: Y = 18.88*X + 48.89) and a potentially positive correlation between the expression of hsa-miR-501-3p and the age of onset in patients with ALS (*r* = 0.3012, *p* = 0.0557, simple linear regression: Y = 19.85*X + 47.80) (Supplementary Figure 4A). Moreover, we found the expression of hsa-miR-30b-5p, and the disease progression in patients with ALS showed a potentially positive correlation (*r* = 0.2499, *p* = 0.0521, simple linear regression: Y = 0.5408*X + 0.9906) and a potentially negative correlation between the expression of hsa-miR-30b-5p and ALSFRS scores (*r* = −0.2169,95% CI: −0.4524 to 0.04684, *p* = 0.096), although they did not reach statistical significance (Supplementary Figure 4B).

### Prediction of miRNA target genes and functional analyses

**FIGURE 5:**
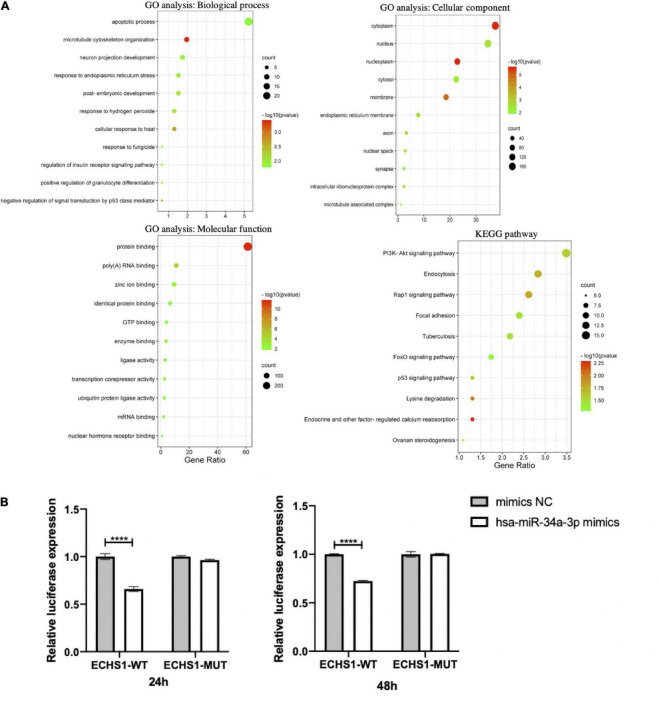
The downstream prediction and validation of hsa-miR-34a-3p. **(A)** The functional analysis including GO and KEGG analyses of target genes predicted hsa-miR-34a-3p. **(B)** Preliminary validation of putative target gene *ECHS1* of hsa-miR-34a-3p by Dual-Luciferase reporter assay. The result showed that hsa-miR-34a-3p bound to ECHS1 at both 24 h (*p* = 0.0001) and 48 h (*p* < 0.0001) and caused a statistically significant decrease in fluorometric intensity. ^****^p < 0.0001.

To speculate on how hsa-miR-34a-3p, hsa-miR-1306-3p, hsa-miR-199a-3p, hsa-miR-30b-5p, and three potentially statistically significant miRNAs (hsa-miR-501-3p, hsa-miR-103a-2-5p, and hsa-miR-181d-5p) participate in the pathogenesis of ALS through downstream genes, we used three different online miRNA databases, namely, TargetScan, miRBD, and Tarbase, to perform the prediction and took the predicted target genes, which were the subset of at least two databases to conduct functional analysis. The predicted target genes and Venn diagrams of these seven miRNAs (hsa-miR-34a-3p, hsa-miR-1306-3p, hsa-miR-199a-3p, hsa-miR-30b-5p, hsa-miR-501-3p, hsa-miR-103a-2-5p, and hsa-miR-181d-5p) can be found in Supplementary Figure 5 and Supplementary Texts 1−7. Functional analysis, including GO and KEGG analyses of hsa-miR-34a-3p, is shown in Figure 5A. GO and KEGG analyses of six other differentially expressed miRNAs (hsa-miR-1306-3p, hsa-miR-199a-3p, hsa-miR-30b-5p, hsa-miR-501-3p, hsa-miR-103a-2-5p, and hsa-miR-181d-5p) between patients with ALS and HCs are shown in Supplementary Figures 6−11. Of these, GO analysis of the biological process showed that apoptotic process and RNA transport were at the top for hsa-miR-34a-3p and hsa-miR-1306-3p, respectively. We also provided a graphical summary of five differentially expressed miRNAs’ KEGG network (Supplementary Figure 12). The predominant pathways, i.e., MAPK signaling pathway for hsa-miR-181d-5p, hsa-miR-30b-5p, hsa-miR-199a-3p, and hsa-miR-103a-2-5p; PI3K-Akt signaling pathway for hsa-miR-34a-3p, hsa-miR-181d-5p, and hsa-miR-199a-3p; and axon guidance for hsa-miR-30b-5p and hsa-miR-103a-2-5p, were also reported in an *in vitro* hSOD1-mutant model (Peviani et al., 2014) and participated in the aggregation of TDP43 (Aaron et al., 2016).

### Preliminary validation of putative target of hsa-miR-34a-3p

We conducted experimental validation of the putative target gene of hsa-miR-34a-3p. Enoyl-CoA Hydratase, Short Chain 1 (*ECHS1*) was predicted as a target gene of hsa-miR-34a-3p by three databases mentioned earlier. The predicted binding site of hsa-miR-34a-3p to ECHS1 was located in the 355-362 region of the *ECHS1* 3′ UTR from the TargetScan online database and the site binding type was 8mer. The dual-Luciferase reporter assay validated that hsa-miR-34a-3p could bind to *ECHS1* at both 24 h (*p* = 0.0001) and 48 h (*p* < 0.0001) and cause a statistically significant decrease in fluorometric intensity (Figure 5B).

### SVM model to aid in the diagnosis of ALS

**FIGURE 6:**
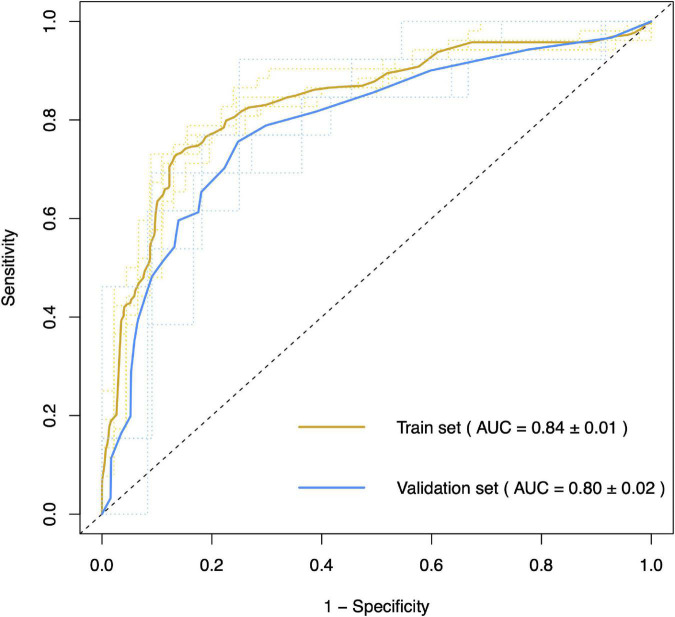
Area under curve of support vector machine model for differentiating patients with and without ALS. The 5-fold cross-validation support vector machine model classified patients with ALS and non-ALS participants in the test cohort with an accuracy of 78.67% (AUC = 0.80).

In the SVM model, we selected five miRNAs, including hsa-miR-199a-3p, hsa-miR-30b-5p, hsa-miR-501-3p, hsa-miR-103a-2-5p, and hsa-miR-181d-5p, as differentially expressed or potentially differentially expressed in patients with SALS and characterized to distinguish patients with SALS from HCs. Patients were classified into a training cohort and a validation cohort in a ratio of 4:1. The 5-fold cross-validated SVM model separating patients with ALS from non-ALS participants in the test cohort had an AUC of 0.80 and an accuracy of 78.67% (Figure 6). The sensitivity and specificity of the model for the diagnosis of ALS were 0.72 and 0.86, respectively. The detailed data of this model’s performance are shown in Supplementary Table 5.

## Discussion

Here, we presented a study showing the differentially expressed miRNAs in SALS and ALS patients with *SOD1*/*C9orf72* mutations and potential disease biomarkers for miRNAs derived from circulating exosomes in ALS. In this study, hsa-miR-34a-3p was exclusively downregulated in patients with *SOD1*-mutated ALS and hsa-miR-1306-3p was downregulated in ALS patients with *SOD1* and *C9orf72* mutations. Alternatively, hsa-miR-199a-3p and hsa-miR-30b-5p were confirmed to be significantly upregulated in patients with SALS. Furthermore, hsa-miR-501-3p was particularly significantly increased in ALS patients with the bulbar-onset group and the bulbar phenotype. The expression of hsa-miR-501-3p showed a potentially positive correlation with the age at onset, and the expression of hsa-miR-30b-5p showed a potentially positive correlation with disease progression and a potentially negative correlation with ALSFRS scores. In practice, the SVM model provided us with differential diagnostic performance between patients with ALS and HCs with an accuracy of 78.67% and an AUC of 0.80.

Exosomes, a subtype of EVs, are membrane-enclosed vehicles containing abundant proteins, lipids, and nucleic acids, which can participate in the bidirectional brain-periphery cross-talk. Owing to the ability of cell targeting to transfer materials (Sluijter et al., 2014), exosomes and their cargos were investigated in cancer, immune, and neurodegenerative diseases (Holm et al., 2018). Previously, efforts have been made to confirm exosomes may contribute to the spreading of toxic protein aggregates, such as TARDBP (Nonaka et al., 2013; Feneberg et al., 2014), SOD1 (Gomes et al., 2007), as well as the clearance of the toxic protein aggregates (Thompson et al., 2016). *TDP-43* has been reported to cooperate with other microprocessors such as Drosha and Dicer to promote miRNA biogenesis (Kawahara and Mieda-Sato, 2012). ALS pathogenesis may be a cause or consequence of the disrupted biological processes of miRNA, including synthesis, maturation, and degradation. Hence, detecting potential miRNA biomarkers in ALS could open up a whole new area of knowledge to help gain a better understanding of disease pathophysiology. The present study characterized the miRNA signature identified from exosomes which was collected from SALS and gene-mutated ALS. It is known that it is difficult and challenging to isolate low-concentration miRNAs from exosomes/biofluids efficiently. The isolation method used in our study was a commercial kit called exoRNeasy Serum/Plasma Midi Kit (EXR) from Qiagen (Germany). Ding et al. (2018) compared four commonly used commercial kits for exosomal miRNA profile and reported that EXR performed better in the specific exosomal miRNAs recovery; among four commercial kits for miRNA extraction from exosomes, EXR achieved a better correlation of the results obtained from serum and plasma samples. In addition, exoRNeasy is a spin column-based method for the isolation of total RNA from EVs in serum and plasma. This method isolates highly pure RNA of equal or higher quantity compared to the traditional method of ultracentrifugation, with high specificity for vesicular over non-vesicular RNA. This method has been an improvement over traditional methods in providing a faster, more standardized way to achieve reliable high-quality RNA preparations from EVs in biofluids such as serum and plasma (Enderle et al., 2015).

In this study, hsa-miR-34a-3p was found to be downregulated in patients with *SOD1*-mutated ALS, whereas it was found to be increased in patients with *C9orf72*-mutated ALS despite not achieving the statistical significance. Pioneering work on hsa-miR-34a was consistent with our findings. Zhou et al. (2018) found that miR-34a was decreased at three different stages of disease in the spinal cord and the hypoglossal, facial, and red nuclei of *SOD1*^G93A^ mice compared with *SOD1*^WT^ mice. Moreover, Rizzuti et al. (2018) reported that hsa-miR-34a was downregulated in motor neuron progenitors differentiated from human ALS-induced pluripotent stem cells. Inversely, miR-34a-5p was overexpressed in patients with *C9orf72* mutation and pre-symptomatic carriers compared with HCs, suggesting that miR-34a-5p expression was dysregulated in cases with *C9orf72* mutation (Kmetzsch et al., 2021). All of the above indicated the differential pathogenesis caused by *SOD1* and *C9orf72* mutations, although further studies are needed. Mechanically, miR-34a was also reported to be involved in the apoptotic signaling pathway, including the downregulation of BCL-2 expression in a double transgenic mouse model of Alzheimer’s disease (AD) (Wang et al., 2009) and in other neurological diseases (Mollinari et al., 2015; Modi et al., 2016), all of which being consistent with the GO analysis in this study showed that hsa-miR-34a-3p was linked with the apoptotic process and neuron projection development. Thus, miR-34a, an apoptosis-related miRNA, also regarded as a tumor suppressor or senescence inducer, was thought to involve in the p53 pathway (Raver-Shapira et al., 2007), synaptic vesicle regulation, and oxidative stress pathway, which might participate in the pathological mechanism of ALS. Hsa-miR-34a-3p was a specific differentially expressed miRNA found in ALS patients with *SOD1* mutation, suggesting that there might be a different pathogenesis between ALS with gene mutation and SALS, even underly ALS patients with *SOD1* and *C9orf72* mutations. Interestingly, hsa-miR-1306-3p showed downregulation significantly in patients with gene mutation, both in *SOD1* and *C9orf72* groups, but not in the SALS group. To date, hsa-miR-1306-3p has been reported in AD and cerebral ischemia/reperfusion injury in neurological diseases. Significant downregulation of hsa-miR-1306-5p was found in EVs (Li F. et al., 2020) and serum (Cheng L. et al., 2015) from patients with AD compared to HCs. The regulation of ADAM10 by hsa-miR-1306 was demonstrated in SH-SY5Y cells expressing the 3’UTR of ADAM10 under a luciferase reporting vector (Augustin et al., 2012). ADAM10, an essential AD gene, controls the proteolytic processing of amyloid-beta precursor protein and the formation of the amyloid plaques (Pereira Vatanabe et al., 2021). In addition, miR-1306-5p expression was significantly downregulated in the oxygen-glucose deprivation/reoxygenation-induced SH-SY5Y cell model, and the upregulation of miR-1306-5p could decrease cerebral ischemia/reperfusion injury (Chen et al., 2019), suggesting miR-1306-5p involves in the cell survival rate and inhibits the cell injury. GO analysis revealed that the top group of target genes for hsa-miR-1306-3p has process networks for RNA transport and mRNA export from the nucleus, which was also consistent with the theory that *C9orf72* is involved in ALS (Vatsavayai et al., 2019). In addition, RNA processing, including transportation and clear, especially proteins belonging to hnRNP classes, were reported to participate in the pathological biological processes or pathways of gene-associated ALS (*TARDBP* and *FUS*) (Guerreiro et al., 2015). Hsa-miR-199a-3p was confirmed to be upregulated significantly in patients with SALS by the microarray and RT-qPCR. In a previous study, miR-199a-3p expression was reported to be downregulated in Parkinson’s disease (PD) (Zhou et al., 2021); more exactly, it was specifically downregulated in stage II of PD and miR-199a-3p overexpression inhibited the cell apoptosis induced by MPP+ treatment and promoted cell proliferation (He et al., 2021). Moreover, miR-199a-3p has also been regarded as a tumor suppressor in various human cancers (Shatseva et al., 2011). The functional analysis showed that the potentially predicted genes of miR-199a-3p were involved in the transcription of DNA-template, signal transduction, and positive regulation of transcription from RNA polymerase II promoter, suggesting that they might be of importance in cell proliferation and migration. Therefore, we speculated that the elevation of miR-199a-3p found in patients with SALS might be a secondary compensatory response to alleviate the cell damage caused by the disease. Compared with HCs, hsa-miR-30b-5p was found to be upregulated in SALS. Interestingly, Brennan et al. (2019). reported that hsa-miR-30b-5p was the single miRNA that overlapped between all four NDS, including AD, PD, multiple sclerosis, and ALS, which suggested hsa-miR-30b-5p may involve in the same pathogenesis of these four NDS. Remarkably, KEGG analysis showed that hsa-miR-30b-5p was represented in the axon guidance, ubiquitin-mediated proteolysis, and ALS. Thus, we should pay more attention to hsa-miR-30b-5p and try to dig into the pathogenesis underlying the dysfunction of hsa-miR-30b-5p. Except for miRNAs discussed earlier, some other miRNAs were also reported to be differentially expressed in patients with ALS compared with HCs, such as miR-27a-3p, which was found as a downregulation miRNA in serum exosomes from patients with ALS (Xu et al., 2018). The reason for the diverse findings might be the sample size and the patients with different disease durations.

Regarding the clinical features, patients who were at bulbar-onset showed a higher expression in hsa-miR-501-3p. Similarly, patients with ALS classified with bulbar phenotype had a higher expression in hsa-miR-501-3p than those with classic phenotype. The result suggests that hsa-miR-501-3p could be a biomarker of disease classification, which will benefit early identification, improve the quality of life, and avoid complications for the bulbar-onset subgroup. In addition, miR-501-3p was reported to be dysregulated in the serum of patients with AD and positively correlated with the Mini-Mental State Examination, suggesting it might be a biomarker related to the progression of AD (Hara et al., 2017). Interestingly, we also found a positive correlation between this miRNA and the onset age in patients with ALS, suggesting miR-501-3p might be associated with aging and degeneration.

The support vector machine is a powerful tool that can analyze data whose number of predictors is approximately equal to or greater than the number of observations (Suh et al., 2018), commonly used in radiomics. SVM constructs hyperplanes of the covariates’ space that separate the observations according to their category (Huang et al., 2018). The current study highlighted the diagnostic relevance of selected miRNAs, which could be used for the clinical diagnosis of patients with ALS. Using five differentially expressed miRNA biomarkers in combination as a signature to classify the disease state of our samples, we observed an almost 0.80 classification accuracy between ALS and non-ALS samples and an AUC of 0.80 after 5-fold cross-validation in the test cohort. This is significant as it demonstrates that these biomarkers may prove useful in diagnosing the disease, but still, challenges are needed to be met. Patients with other NDS are needed to be as disease controls to verify whether these diagnostic tool work. And more detailed classification might enhance the predictive accuracy and increase miRNAs concentration from exosomes are needed.

Although our study provided valuable results, it did have some drawbacks. First, miRNA expression in exosomes was only collected at baseline, so we were unable to obtain the dynamics of miRNA expression in exosomes over time. Moreover, we do not know if these miRNAs varied before the onset of symptoms. Thus, more follow-up data, even in asymptomatic individuals with pathogenic mutations, and a functional study of selected miRNAs in the ALS model are the next steps that are envisioned. Second, the miRNA profile in exosomes might be altered by the isolation method, and the protocol used in our study was a spin column-based method for the isolation of miRNAs from the plasma. Further study is needed to validate the expression of the miRNAs reported in the current study from exosomes purified with a different protocol. While recent years have seen unparalleled advances in the isolation and analysis of miRNAs from exosomes, challenges such as how efficiently low-concentration miRNAs can be isolated from exosomes and the heterogeneity of miRNAs derived from exosomes still remain unresolved. Third, we noticed that all differentially expressed miRNAs identified in the study were also reported in other NDS, which might reflect an underlying change that occurs as a result of neuron breakdown. This might be the reason that the predictive accuracy of the comparison between patients with ALS and HCs was only 78.67%, which was a little low as a diagnostic tool. The pathological mechanisms underlying these aberrant miRNAs need to be further studied. In addition, we have only conducted the internal validation of five miRNA biomarkers by the SVM model. Exploring these dysregulated miRNAs in another external validation cohort of ALS will be considered to enhance their diagnostic potential for this disease.

## Conclusion

Overall, our results confirmed a key role in the unique signature of miRNAs from circulating exosomes in patients with ALS. By combining microarray and RT-qPCR results, we reported the dysregulation of hsa-miR-34a-3p and hsa-miR-1306-3p in ALS patients with *SOD1*/*C9orf72* mutations, and hsa-miR-199a-3p and hsa-miR-30b-5p in patients with SALS, suggesting the different mechanisms underlying the gene-mutant ALS and SALS. The machine-learning algorithms have high accuracy in the prediction of ALS diagnosis and, if replicated in further studies, will provide the basis for the clinical application of blood tests in ALS diagnosis and ALS classification diagnosis. Identification of the downstream genetic pathways predicted by candidate miRNAs might lead to the discovery of pathological mechanisms and the development of therapeutic strategies.

## Data availability statement

The datasets presented in this article are not readily available because of the ethical restrictions of West China hospital. Requests to access the datasets should be directed to the authors/corresponding authors.

## Ethics statement

The studies involving human participants were reviewed and approved by the Ethics Committee of West China Hospital of Sichuan University (approval number 2016-097). The patients/participants provided their written informed consent to participate in this study.

## Author contributions

H-FS and Y-PC contributed to the conceptualization, carried out the funding acquisition, wrote, reviewed, and edited the manuscript, and supervised the data. X-JG, T-MY, Q-QW, BC, and YZ performed the methodology, carried out the software, and validated and investigated the data. Y-FC carried out the formal analysis and data curation, wrote the original draft, and investigated the data. All authors read and approved the final manuscript.

## Footnotes

1
www.targetscan.org/vert_72/

2
https://dianalab.e-ce.uth.gr/html/diana/web/index.php?r=tarbasev8

3
www.mirdb.org

4
ngs.ym.edu.tw/ym500v2/index.php

5
bioinformatics.psb.ugent.be/webtools/Venn/

6
https://david.ncifcrf.gov/home.jsp

7
www.bioinformatics.com.cn

8
http://ngs.ym.edu.tw/ym500v2/sample.php
