# Supplementary material for: Signature of miRNAs derived from the circulating exosomes of patients with amyotrophic lateral sclerosis
Source: Front Aging Neurosci. 2023 Feb 10;15:1106497. doi: 10.3389/fnagi.2023.1106497 (PMC9951117; doi:10.3389/fnagi.2023.1106497)
Supplement: Supplementary file 1 [file Data_Sheet_1.docx]

**Supplementary Table 1:**

The detail of genetic data of gene-mutated ALS patients.

| **Screening cohort** | **Mutation gene** | **Mutation site** | **Amino acid change** | **Family History** | **Note** |
| --- | --- | --- | --- | --- | --- |
| 1 | SOD1 | c.341T>C | p.Ile114Thr | No |  |
| 2 | SOD1 | c.455T>G | p.Ile152Ser | Yes | The younger brother presented ALS-like symptoms and died at 39. |
| 3 | SOD1 | c.140A>G | p.His47Arg | Yes | The *SOD1*-mutated pedigree. |
| 4 | C9orf72 |  |  | No |  |
| 5 | C9orf72 |  |  | No |  |
| 6 | C9orf72 |  |  | No |  |
| Validation cohort |  |  |  |  |  |
| 1 | SOD1 | c.16G>T | p.Val6Leu | No |  |
| 2 | SOD1 | c.208A>G | p.Arg70Gly | No |  |
| 3 | SOD1 | c.199C>T | p.Pro67Ser | No | The mother was diagnosed depression. |
| 4 | SOD1 | c.140A>G | p.His47Arg | No |  |
| 5 | SOD1 | c.199C>G | p.Pro67Ala | No |  |
| 6 | SOD1 | c.255G>C | p.Ala85Phe | YES | The mother presented ALS-like symptoms and died at 44 after 4 years of the symptom onset. |
| 7 | SOD1 | c.404G>A | p.Ser135Asn | No |  |
| 8 | SOD1 | c.14C>T | p.Ala5Val | No |  |
| 9 | C9orf72 |  |  | Yes | The father presented dementia symptoms and died at 81years old. |
| 10 | C9orf72 |  |  | No |  |
| 11 | C9orf72 |  |  | No |  |
| 12 | C9orf72 |  |  | No |  |
| 13 | C9orf72 |  |  | No |  |
| 14 | C9orf72 |  |  | No |  |
| 15 | C9orf72 |  |  | No |  |
| 16 | C9orf72 |  |  | No |  |

**Supplementary Table 2:**

The differentially expressed miRNAs screened by micro-array from *SOD1*-ALS v.s HCs, *C9orf72*-ALS v.s HCs and overlapped dysregulated miRNAs both in *SOD1* and *C9orf72* ALS compared with HCs.

| **Groups** | **Total** | **Up-regulated miRNAs** | **Down-regulated miRNAs** |
| --- | --- | --- | --- |
| Overlapped both in *SOD1* & *C9orf72* | 11 | hsa-miR-6848-3p hsa-miR-34a-3p hsa-miR-3926 hsa-miR-1915-3p | hsa-miR-4455 hsa-miR-103a-2-5p hsa-miR-4729 hsa-miR-6824-5p hsa-miR-1306-3p hsa-miR-501-3p hsa-miR-181d-5p |
| *SOD1*-ALS v.s HCs | 53 | hsa-miR-6829-5p hsa-miR-3926 hsa-miR-5579-5p hsa-miR-520d-3p hsa-miR-7152-5p hsa-miR-7856-5p hsa-miR-153-5p hsa-miR-4740-3p hsa-miR-3686 hsa-miR-4646-3p hsa-miR-1253 hsa-miR-34a-3p hsa-miR-4664-3p hsa-miR-1976 hsa-miR-583 hsa-miR-4506 hsa-miR-6848-3p hsa-miR-576-5p hsa-miR-6858-3p hsa-miR-6756-3p hsa-miR-4739 hsa-miR-500a-5p hsa-miR-6511a-5p hsa-miR-6895-5p hsa-miR-6760-5p hsa-miR-509-3-5p hsa-miR-548k hsa-miR-1915-3p hsa-miR-550b-2-5p hsa-miR-7111-3p hsa-miR-4708-5p hsa-miR-4313 hsa-miR-4745-5p hsa-miR-4539 hsa-miR-6736-5p | hsa-miR-7110-5p hsa-miR-1227-5p hsa-miR-1307-3p hsa-miR-1260b hsa-miR-1587 hsa-miR-2110 hsa-miR-501-3p hsa-miR-103a-2-5p hsa-miR-181d-5p hsa-miR-4455 hsa-miR-3141 hsa-miR-652-5p hsa-miR-3064-5p hsa-miR-3928-3p hsa-miR-7977 hsa-miR-340-5p hsa-miR-660-3p hsa-miR-1273f hsa-miR-4647 hsa-miR-3907 hsa-miR-7160-5p hsa-miR-6722-5p hsa-miR-1306-3p hsa-miR-29b-3p hsa-miR-4729 hsa-miR-2681-5p hsa-miR-223-5p hsa-miR-6824-5p hsa-miR-5095 |
| *C9orf72*-ALS v.s HCs | 117 | hsa-miR-6848-3p hsa-miR-4524a-3p hsa-miR-505-3p hsa-miR-6514-3p hsa-miR-520a-3p hsa-miR-4794 has-miR-7161-3p hsa-miR-362-3p hsa-miR-6764-5p hsa-miR-2052 hsa-miR-6769b-3p hsa-miR-34a-3p hsa-miR-302d-5p hsa-miR-6788-3p hsa-miR-1255a hsa-miR-6857-3p hsa-miR-449c-5p hsa-miR-3074-3p hsa-miR-6747-5p hsa-miR-2053 hsa-miR-6081 hsa-miR-4733-5p hsa-miR-1233-3p hsa-miR-616-5p hsa-miR-449b-3p hsa-miR-607 hsa-miR-3148 hsa-miR-5590-3p hsa-miR-3150b-5p hsa-miR-150-5p hsa-miR-323b-3p hsa-miR-6813-3p hsa-miR-604 hsa-miR-3926 hsa-miR-3199 hsa-miR-1252-3p hsa-miR-6798-3p hsa-miR-574-5p hsa-miR-296-5p hsa-miR-1233-5p hsa-miR-6867-3p hsa-miR-4687-5p hsa-miR-8087 hsa-miR-4635 hsa-miR-4695-3p hsa-miR-664b-3p hsa-miR-6754-3p hsa-miR-548as-3p hsa-miR-3151-5p hsa-miR-4324  hsa-miR-3940-3p hsa-miR-4722-3p hsa-miR-6786-5p hsa-miR-762 hsa-miR-636  hsa-miR-3620-5p hsa-miR-149-3p hsa-miR-4763-3p hsa-miR-4707-5p hsa-miR-4749-3p hsa-miR-210-5p hsa-miR-6756-5p hsa-miR-6789-5p hsa-miR-3162-3p hsa-miR-7108-5p hsa-miR-877-5p hsa-miR-2861 hsa-miR-6869-5p hsa-miR-6791-5p has-miR-6088 hsa-miR-1234-3p hsa-miR-6724-5p hsa-miR-4687-3p hsa-miR-4516 hsa-miR-4274 hsa-miR-4433-5p hsa-miR-6805-3p hsa-miR-4466 hsa-miR-6090 hsa-miR-3665 hsa-miR-1915-3p hsa-miR-8069 hsa-miR-1228-3p hsa-miR-5001-5p | hsa-miR-199a-3p hsa-miR-199b-3p hsa-miR-18a-5p hsa-miR-744-5p hsa-miR-30b-5p hsa-let-7a-5p hsa-miR-200c-3p hsa-miR-4633-5p hsa-miR-505-5p hsa-miR-503-5p hsa-miR-488-3p hsa-miR-4717-3p hsa-miR-378c hsa-miR-501-3p hsa-miR-885-3p hsa-miR-4436a hsa-miR-485-5p hsa-miR-1306-3p hsa-miR-181d-5p hsa-miR-890 hsa-miR-514b-5p hsa-miR-103a-2-5p hsa-miR-7703 hsa-miR-6769b-5p hsa-miR-4455 hsa-miR-4684-3p hsa-miR-4713-3p hsa-miR-548an hsa-miR-374c-5p hsa-miR-6856-5p hsa-miR-6861-5p hsa-miR-197-5p hsa-miR-6824-5p hsa-miR-181c-3p hsa-miR-4436b-3p hsa-miR-4729 hsa-miR-4731-5p hsa-miR-4633-3p hsa-miR-15a-3p hsa-miR-5705 hsa-miR-4685-5p hsa-miR-1909-3p hsa-miR-541-3p hsa-miR-548az-5p |

**Supplementary Table 3:**

More details including target genes related to ALS, binding site type and target region of candidate miRNAs predicted by online miRNA databases.

| **Source** | **miRNA** | **Online database** | **Predicted target gene** | **Predicted binding site** | **miRNA binding site type** | **Predicted consequential pairing of target region** |
| --- | --- | --- | --- | --- | --- | --- |
| SOD1-ALS exclusively | hsa-miR-3928-3p | TargenScan | FUS | Position 172-178 of FUS 3' UTR | 7mer-m8 | 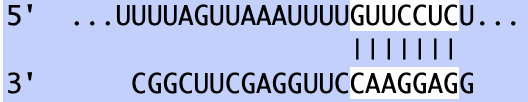 |
|  | hsa-miR-340-5p | TargenScan | SOD1 | Position 185-191 of SOD1 3' UTR | 7mer-A1 | 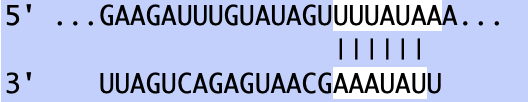 |
| C9orf72-ALS exclusively | hsa-miR-199a-3p | TargenScan | C9orf72 | Position 296-302 of C9orf72 3' UTR | 7mer-m8 | 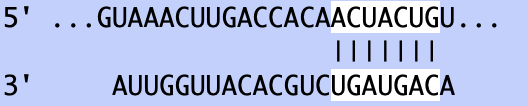 |
|  | hsa-miR-30b-5p | TargenScan | C9orf72 | Position 227-234 of C9orf72 3' UTR | 8mer | 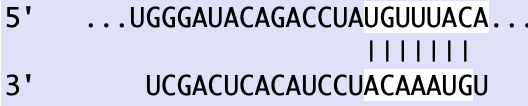 |
|  | hsa-miR-485-5p | TargenScan | FUS | Position 907-913 of FUS 3' UTR | 7mer-m8 | 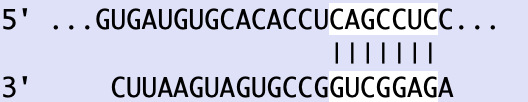 |
| Overlapped both in SOD1 and C9orf72-ALS | hsa-miR-1915-3p | TargenScan | C9orf72 | Position 573-580 of C9orf72 3' UTR | 8mer | 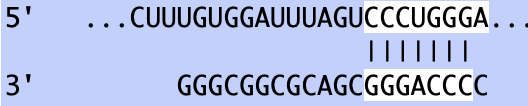 |
|  | hsa-miR-181d-5p | TargenScan | TARDBP | Position 1047-1053 of TARDBP 3' UTR | 7mer-m8 | 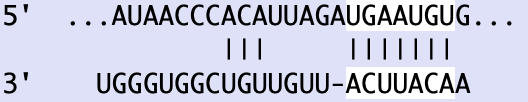 |
|  | hsa-miR-4729 | TargenScan | FUS | Position 350-357 of FUS 3' UTR | 8mer | 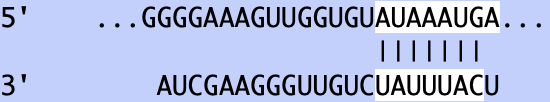 |
|  | hsa-miR-4455 | TargenScan | UBQLN2 | Position 62-74 of UBQLN2 3' UTR | non- canonical | 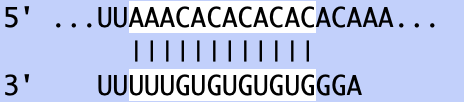 |
|  | hsa-miR-34a-3p | TargenScan | C9orf72 | Position 1181-1187 of C9orf72 3' UTR | 7mer-A1 | 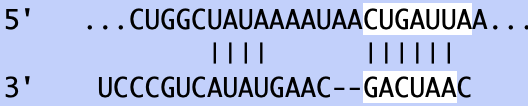 |
|  | hsa-miR-1306-3p | TargenScan | TARDBP | Position 23-29 of TARDBP 3' UTR | 7mer-A1 | 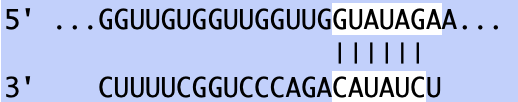 |
|  | hsa-miR-6824-5p | TargenScan | TARDBP | Position 3246-3252 of TARDBP 3' UTR | 7mer-A1 | 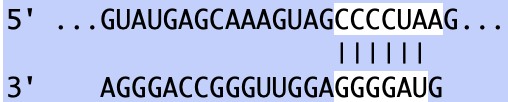 |
|  | hsa-miR-501-3p | TargenScan | TARDBP | Position 865-872 of TARDBP 3' UTR | 8mer | 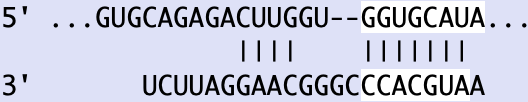 |
|  | hsa-miR-103a-2-5p | TargenScan | TARDBP | Position 564-571 of TARDBP 3' UTR | 8mer | 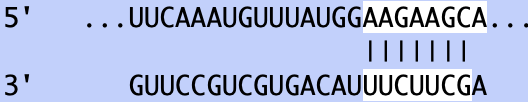 |

**Supplementary Table 4:** Hemolysis assessment of samples analyzed and the stability of external control (cel-miR-39-3p) and internal control (hsa-miR-16-5p).

| Training set | Original Ct of cel-miR-39-3p (external control) | Original Ct of hsa-miR-16-5p (internal control) | Abs<0.080 | 0.080<Abs<0.3 | Delta Ct (miR-23a/miR-451) | Hemolysis status |
| --- | --- | --- | --- | --- | --- | --- |
| *SOD* 1-ALS 1 | / | / | ✔️ |  | 0.90 | Pass |
| *SOD* 1-ALS 2 | / | / |  | ✔️ | 3.41 | Pass |
| *SOD* 1-ALS 3 | / | / |  | ✔️ | 2.35 | Pass |
| *C9orf72*-ALS 1 | / | / |  | ✔️ | 2.74 | Pass |
| *C9orf72*-ALS 2 | / | / | ✔️ |  | 1.45 | Pass |
| *C9orf72*-ALS 3 | / | / | ✔️ |  | 1.73 | Pass |
| HC 1 | / | / | ✔️ |  | 1.32 | Pass |
| HC 2 | / | / |  | ✔️ | 1.63 | Pass |
| HC 3 | / | / | ✔️ |  | 0.95 | Pass |
| Validation set | | | | | | |
| sALS 1 | 13.58 | 18.6 | ✔️ |  | 0.62 | Pass |
| sALS 2 | 14.16 | 19.3 |  | ✔️ | 4.75 | Pass |
| sALS 3 | 14.51 | 17.93 |  | ✔️ | 2.13 | Pass |
| sALS 4 | 13.65 | 18.11 | ✔️ |  | 1.98 | Pass |
| sALS 5 | 13.58 | 18.37 | ✔️ |  | 2.53 | Pass |
| sALS 6 | 15.24 | 18.69 | ✔️ |  | 2.40 | Pass |
| sALS 7 | 15.08 | 17.12 | ✔️ |  | 2.49 | Pass |
| sALS 8 | 14.42 | 18.1 | ✔️ |  | 2.24 | Pass |
| sALS 9 | 14.22 | 17.21 |  | ✔️ | 3.24 | Pass |
| sALS 10 | 12.39 | 17 |  | ✔️ | 4.18 | Pass |
| sALS 11 | 13.58 | 19.31 | ✔️ |  | 1.74 | Pass |
| sALS 12 | 11.39 | 19.75 |  | ✔️ | 1.15 | Pass |
| sALS 13 | 12.55 | 15.1 | ✔️ |  | 1.46 | Pass |
| sALS 14 | 13.72 | 18.07 | ✔️ |  | 2.69 | Pass |
| sALS 15 | 14.21 | 17.28 | ✔️ |  | 1.59 | Pass |
| sALS 16 | 13.64 | 16.97 | ✔️ |  | 2.44 | Pass |
| sALS 17 | 13.02 | 18.29 | ✔️ |  | 1.75 | Pass |
| sALS 18 | 13.15 | 18.51 |  | ✔️ | 2.41 | Pass |
| sALS 19 | 14.77 | 19.15 | ✔️ |  | 1.76 | Pass |
| sALS 20 | 14.05 | 17.85 |  | ✔️ | 3.47 | Pass |
| sALS 21 | 13.47 | 19.23 |  | ✔️ | 3.60 | Pass |
| sALS 22 | 15.6 | 19.09 |  | ✔️ | 1.71 | Pass |
| sALS 23 | 15.52 | 19.82 | ✔️ |  | 1.51 | Pass |
| sALS 24 | 14.02 | 18.04 | ✔️ |  | 1.10 | Pass |
| sALS 25 | 13.49 | 19.51 |  | ✔️ | 4.46 | Pass |
| sALS 26 | 14.47 | 18.43 | ✔️ |  | 5.47 | Pass |
| sALS 27 | 13.89 | 19.93 |  | ✔️ | 4.69 | Pass |
| sALS 28 | 15.84 | 18.16 | ✔️ |  | 2.57 | Pass |
| sALS 29 | 15.36 | 19.54 | ✔️ |  | 3.86 | Pass |
| sALS 30 | 15.05 | 18.44 |  | ✔️ | 4.79 | Pass |
| sALS 31 | 15 | 19.32 |  | ✔️ | 5.10 | Pass |
| sALS 32 | 14.73 | 19.4 |  | ✔️ | 2.59 | Pass |
| sALS 33 | 15.02 | 18.05 | ✔️ |  | 3.65 | Pass |
| sALS 34 | 15.15 | 17.53 | ✔️ |  | 3.26 | Pass |
| sALS 35 | 15.21 | 16.65 | ✔️ |  | 4.55 | Pass |
| sALS 36 | 14.76 | 21.29 | ✔️ |  | 4.15 | Pass |
| sALS 37 | 15.97 | 17.75 |  | ✔️ | 3.73 | Pass |
| sALS 38 | 14.42 | 17.06 | ✔️ |  | 3.95 | Pass |
| sALS 39 | 14.06 | 19.38 |  | ✔️ | 3.80 | Pass |
| sALS 40 | 12.15 | 19.7 | ✔️ |  | 2.38 | Pass |
| sALS 41 | 13.68 | 21.19 | ✔️ |  | 1.25 | Pass |
| sALS 42 | 14.26 | 21.58 | ✔️ |  | 2.87 | Pass |
| sALS 43 | 13.41 | 18.18 |  | ✔️ | 4.02 | Pass |
| sALS 44 | 14.82 | 21.64 | ✔️ |  | 1.47 | Pass |
| sALS 45 | 14.73 | 18.04 |  | ✔️ | 3.61 | Pass |
| sALS 46 | 15.41 | 19 |  | ✔️ | 1.71 | Pass |
| sALS 47 | 13.99 | 21.55 |  | ✔️ | 1.51 | Pass |
| sALS 48 | 14.35 | 21.29 | ✔️ |  | 2.46 | Pass |
| sALS 49 | 14.45 | 21.21 | ✔️ |  | 1.13 | Pass |
| sALS 50 | 15.03 | 20.71 | ✔️ |  | 2.32 | Pass |
| sALS 51 | 16.91 | 21.09 | ✔️ |  | 0.71 | Pass |
| sALS 52 | 16.8 | 20.19 | ✔️ |  | 1.43 | Pass |
| sALS 53 | 16.59 | 19.03 |  | ✔️ | 4.43 | Pass |
| sALS 54 | 16.86 | 22.02 |  | ✔️ | 5.31 | Pass |
| sALS 55 | 15.3 | 20.74 |  | ✔️ | 3.42 | Pass |
| sALS 56 | 14.12 | 21.29 |  | ✔️ | 4.56 | Pass |
| sALS 57 | 14.61 | 20.42 | ✔️ |  | 2.96 | Pass |
| sALS 58 | 15.43 | 19.75 |  | ✔️ | 3.09 | Pass |
| sALS 59 | 15.49 | 20.35 | ✔️ |  | 1.20 | Pass |
| sALS 60 | 15.01 | 20.99 | ✔️ |  | 1.20 | Pass |
| sALS 61 | 15.37 | 20.75 | ✔️ |  | 4.18 | Pass |
| sALS 62 | 14.32 | 20.62 |  | ✔️ | 3.95 | Pass |
| sALS 63 | 14.23 | 20.63 | ✔️ |  | 4.96 | Pass |
| sALS 64 | 15.07 | 20.93 | ✔️ |  | 0.59 | Pass |
| sALS 65 | 15.05 | 20.36 | ✔️ |  | 2.06 | Pass |
| *SOD* 1-ALS 4 | 15.25 | 20.03 | ✔️ |  | 3.35 | Pass |
| *SOD* 1-ALS 5 | 15.08 | 20.01 |  | ✔️ | 4.28 | Pass |
| *SOD* 1-ALS 6 | 15.86 | 20.89 | ✔️ |  | 4.59 | Pass |
| *SOD* 1-ALS 7 | 15.08 | 20.94 |  | ✔️ | 2.08 | Pass |
| *SOD* 1-ALS 8 | 16.42 | 21.91 |  | ✔️ | 3.14 | Pass |
| *SOD* 1-ALS 9 | 14.54 | 19.36 |  | ✔️ | 2.75 | Pass |
| *SOD* 1-ALS 10 | 14.22 | 21.05 | ✔️ |  | 1.04 | Pass |
| *SOD* 1-ALS 11 | 15.27 | 20.64 | ✔️ |  | 3.64 | Pass |
| *C9orf72*-ALS 4 | 14.58 | 20.51 |  | ✔️ | 3.22 | Pass |
| *C9orf72*-ALS 5 | 14.94 | 20.56 | ✔️ |  | 3.44 | Pass |
| *C9orf72*-ALS 6 | 13.32 | 21.62 |  | ✔️ | 3.29 | Pass |
| *C9orf72*-ALS 7 | 14.63 | 19.75 | ✔️ |  | 2.87 | Pass |
| *C9orf72*-ALS 8 | 16.42 | 21.75 |  | ✔️ | 4.74 | Pass |
| *C9orf72*-ALS 9 | 16.46 | 21.95 | ✔️ |  | 3.36 | Pass |
| *C9orf72*-ALS 10 | 16.39 | 21.82 |  | ✔️ | 3.51 | Pass |
| *C9orf72*-ALS 11 | 16.2 | 20.15 |  | ✔️ | 2.96 | Pass |
| HC 4 | 16.96 | 20.25 |  | ✔️ | 3.09 | Pass |
| HC 5 | 16.41 | 16.77 |  | ✔️ | 1.2 | Pass |
| HC 6 | 12.98 | 19.6 | ✔️ |  | 1 | Pass |
| HC 7 | 13.52 | 20.99 | ✔️ |  | 3.92 | Pass |
| HC 8 | 15.79 | 20.46 | ✔️ |  | 2.8 | Pass |
| HC 9 | 15.93 | 17.71 |  | ✔️ | 2.91 | Pass |
| HC 10 | 13 | 18.6 |  | ✔️ | 4.05 | Pass |
| HC 11 | 13.57 | 18.35 |  | ✔️ | 3.74 | Pass |
| HC 12 | 12.52 | 17.66 | ✔️ |  | 2.06 | Pass |
| HC 13 | 12.84 | 19.64 |  | ✔️ | 3.35 | Pass |
| HC 14 | 16.05 | 18.64 | ✔️ |  | 1.28 | Pass |
| HC 15 | 15.62 | 19.59 |  | ✔️ | 4.59 | Pass |
| HC 16 | 15.37 | 20.28 | ✔️ |  | 2.08 | Pass |
| HC 17 | 16.47 | 19.03 | ✔️ |  | 3.14 | Pass |
| HC 18 | 12.98 | 22.02 |  | ✔️ | 2.75 | Pass |
| HC 19 | 13.52 | 20.74 | ✔️ |  | 2.04 | Pass |
| HC 20 | 19.79 | 22.29 | ✔️ |  | 3.64 | Pass |
| HC 21 | 16.93 | 20.42 |  | ✔️ | 3.22 | Pass |
| HC 22 | 13 | 19.75 | ✔️ |  | 3.45 | Pass |
| HC 23 | 13.57 | 20.75 | ✔️ |  | 3.29 | Pass |
| HC 24 | 12.52 | 20.62 |  | ✔️ | 3.47 | Pass |
| HC 25 | 12.84 | 19.36 | ✔️ |  | 4.87 | Pass |
| HC 26 | 16.05 | 21.05 | ✔️ |  | 4.74 | Pass |
| HC 27 | 15.62 | 22.37 | ✔️ |  | 3.36 | Pass |
| HC 28 | 15.37 | 21.32 |  | ✔️ | 3.51 | Pass |
| HC 29 | 16.47 | 21.23 |  | ✔️ | 2.96 | Pass |
| HC 30 | 16.86 | 22.07 |  | ✔️ | 3.09 | Pass |
| HC 31 | 18.3 | 22.05 | ✔️ |  | 1.91 | Pass |
| HC 32 | 16.12 | 22.25 |  | ✔️ | 1.71 | Pass |
| HC 33 | 16.61 | 22.08 | ✔️ |  | 4.89 | Pass |
| HC 34 | 15.43 | 22.86 |  | ✔️ | 4.66 | Pass |
| HC 35 | 15.49 | 22.08 | ✔️ |  | 5.67 | Pass |
| HC 36 | 14.32 | 23.42 | ✔️ |  | 1.3 | Pass |
| HC 37 | 15.23 | 22.01 | ✔️ |  | 3.14 | Pass |
| HC 38 | 14.22 | 20.32 | ✔️ |  | 2.23 | Pass |
| HC 39 | 15.27 | 20.63 | ✔️ |  | 2.89 | Pass |
| HC 40 | 14.58 | 19.6 | ✔️ |  | 1.89 | Pass |
| HC 41 | 14.94 | 19.3 | ✔️ |  | 1.75 | Pass |
| HC 42 | 14.32 | 19.93 | ✔️ |  | 2.74 | Pass |
| HC 43 | 14.63 | 19.11 |  | ✔️ | 5.00 | Pass |
| HC 44 | 16.42 | 20.37 | ✔️ |  | 2.46 | Pass |
| HC 45 | 16.46 | 19.69 | ✔️ |  | 1.95 | Pass |
| HC 46 | 16.39 | 20.12 | ✔️ |  | 3.75 | Pass |
| HC 47 | 16.2 | 19.1 |  | ✔️ | 2.37 | Pass |
| HC 48 | 16.96 | 19.21 | ✔️ |  | 2.18 | Pass |
| HC 49 | 16.41 | 18.87 | ✔️ |  | 4.21 | Pass |
| HC 50 | 12.98 | 19.41 |  | ✔️ | 5.84 | Pass |
| HC 51 | 13.52 | 18.75 | ✔️ |  | 1.33 | Pass |
| HC 52 | 12.72 | 17.1 | ✔️ |  | 1.75 | Pass |
| HC 53 | 16.14 | 19.07 |  | ✔️ | 3.89 | Pass |
| HC 54 | 14.38 | 20.28 | ✔️ |  | 2.18 | Pass |
| HC 55 | 16.66 | 21.97 |  | ✔️ | 5.05 | Pass |
| HC 56 | 15.52 | 19.76 |  | ✔️ | 6.12 | Pass |
| HC 57 | 16.73 | 21.72 | ✔️ |  | 1.58 | Pass |
| HC 58 | 16.46 | 18.89 | ✔️ |  | 0.69 | Pass |
| HC 59 | 14.51 | 21.02 | ✔️ |  | 2.59 | Pass |
| HC 60 | 15.29 | 20.63 | ✔️ |  | 1.44 | Pass |
| HC 61 | 16.29 | 21.34 |  | ✔️ | 4.75 | Pass |
| HC 62 | 15.21 | 20.22 |  | ✔️ | 5.74 | Pass |
| HC 63 | 14.72 | 21.13 | ✔️ |  | 2.90 | Pass |
| HC 64 | 15.43 | 19.98 |  | ✔️ | 2.04 | Pass |

Samples with results of absorbance lower than 0.3 and the delta Ct (miR-23a/miR-451) lower than 7 were suggested to pass the hemolysis test.

**Supplementary material Table 5:** Comparison of differentially expressed miRNAs by means of microarray and RT-qPCR.

|  |  | Microarray | | qRT-PCR | | |
| --- | --- | --- | --- | --- | --- | --- |
|  | Regulation/p-value | SOD1-fALS | C9orf72-fALS | SALS | SOD1-fALS | C9orf72-fALS |
| Overlapped both in SOD1 and C9orf72-ALS | hsa-miR-1915-3p | Up (0.0087863) | Up (0.0029815) | / | / | / |
|  | hsa-miR-181d-5p | Down (0.0233269) | Down (0.013243) | Up (0.0782) | / | / |
|  | hsa-miR-4729 | Down (0.0421926) | Down (0.0237601) | / | / | / |
|  | hsa-miR-4455 | Down (0.0055092) | Down (0.0452519) | / | / | / |
|  | hsa-miR-34a-3p | Up (0.0402462) | Up (0.0372543) | Down (0.032) | Down (0.0022) | / |
|  | hsa-miR-1306-3p | Down (0.0187917) | Down (0.0011582) | / | Down (0.002) | Down (0.0032) |
|  | hsa-miR-6824-5p | Down (0.0151404) | Down (0.0043387) | / | / | / |
|  | hsa-miR-501-3p | Down (0.0425964) | Down (0.0348612) | Up (0.0533) | / | / |
|  | hsa-miR-103a-2-5p | Down (0.0054956) | Down (0.0146218) | Up (0.0603) | / | / |
| C9orf72-ALS exclusively | hsa-miR-199a-3p | / | Down (0.0127442) | Up (0.0003) | / | / |
|  | hsa-miR-30b-5p | / | Down (0.0402851) | Up (0.0474) | / | / |
|  | hsa-miR-485-5p | / | Down (0.049208) | / | / | / |
| SOD1-ALS exclusively | hsa-miR-3928-3p | Down (0.0117261) | / | / | / | / |
|  | hsa-miR-340-5p | Down (0.0417499) | / | / | / | / |

*, significantly differential expressed miRNAs in the ALS validation group compared with healthy controls; /, means *p*-value more than 0.1.

**Supplementary Table 6:**

Performance of support vector machine model with 5-fold cross-validation.

|  | AUC | Accuracy | Sensitivity | Specificity |
| --- | --- | --- | --- | --- |
| Training set | 0.84±0.01 | 0.81 | 0.79 | 0.82 |
| Test set | 0.80±0.02 | 0.79 | 0.72 | 0.86 |

AUC, area under the curve; SD, standard deviation.

**Supplementary Figure 1:**

Characterization of exosomes derived from plasma. a Transmission electron micrograph of exosomes. b Nanoparticle Tracking Analysis (NTA) of exosomes. c Western blot analysis of CD63, CD81 and Calnexin expression in exosomes. Each experiment was repeated 3 times

**
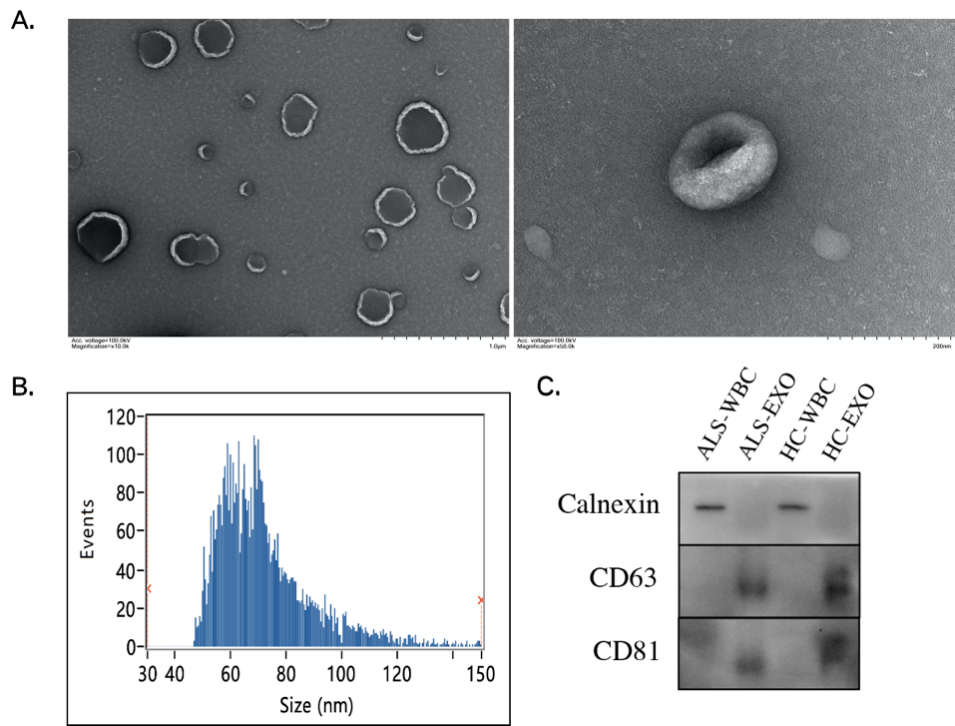
**

**Supplementary Figure 2:**

The Venn diagram of differentially expressed miRNAs screened by micro-array from SOD1-ALS vs. HCs group, C9orf72-ALS vs. HCs group and overlapped dysregulated miRNAs both in SOD1 and C9orf72 ALS compared with HCs.


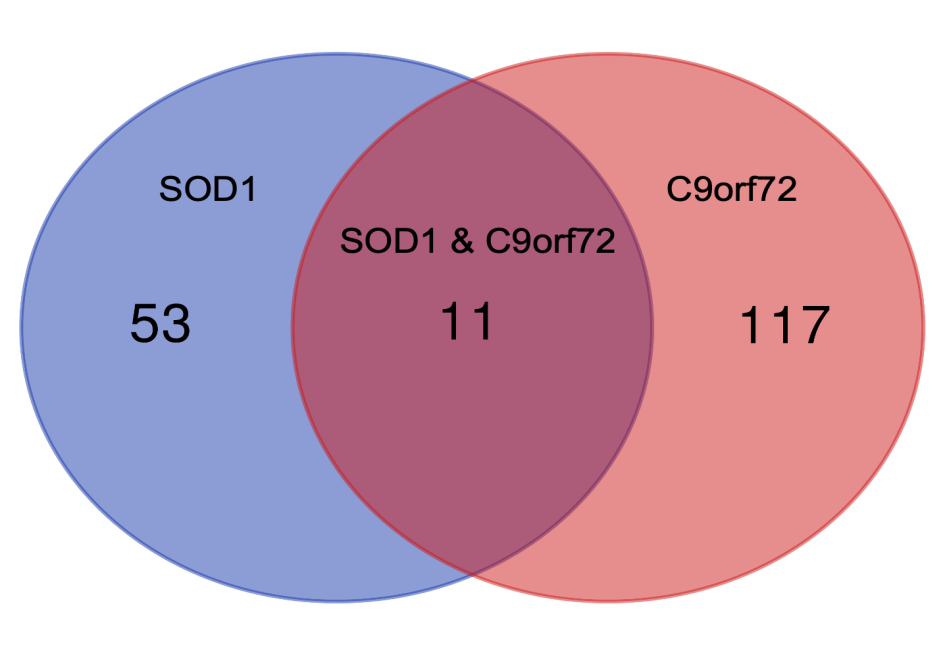


**Supplementary Figure 3:**

Compared with healthy controls, hsa-miR-501-3p(*p*=0.0533), hsa-miR-103a-2-5p(*p*=0.0603) and hsa-miR-181d-5p(*p*=0.0782) were also up-regulated potentially in SALS patients（NALS=61，NHCs=65）


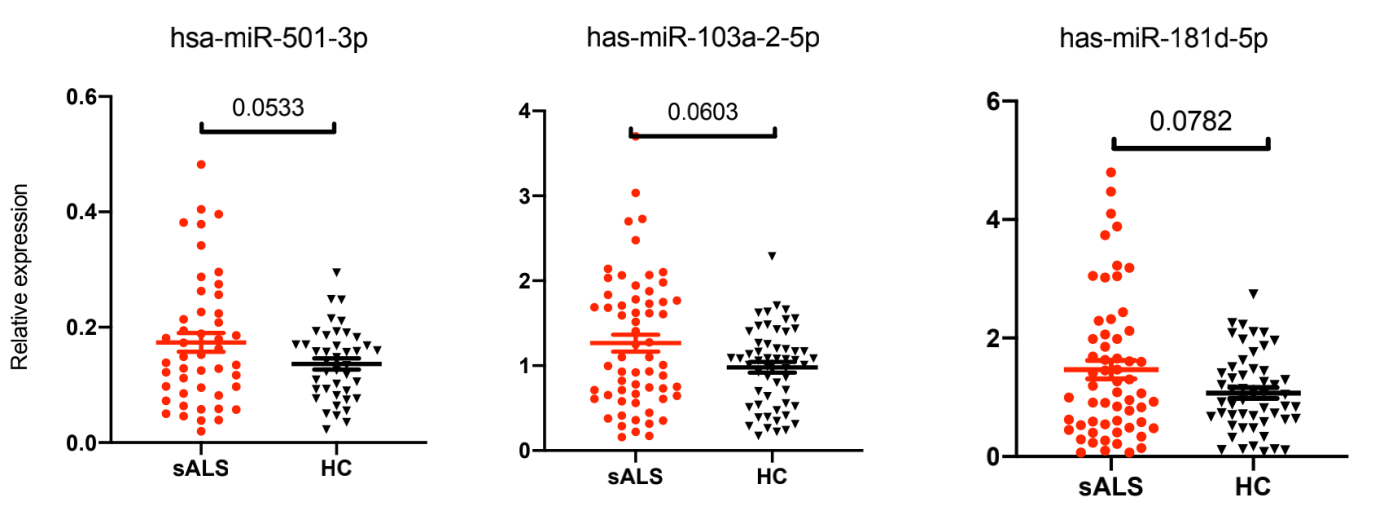


**Supplementary Figure 4**

a. The expression of hsa-miR-501-3p and the age of ALS patients at last assessment showed the positive correlation (r=0.3245, p=0.0384, simple linear regression: Y=18.88*X +48.89) and the expression of hsa-miR-501-3p and the age of onset in ALS patients showed a potential positive correlation (r=0.3012, p=0.0557, simple linear regression: Y=19.85*X +47.80).

b. The expression of hsa-miR-30b-5p and the disease progression of ALS patients showed the potentially positive correlation (r=0.2499, p=0.0521, simple linear regression: Y=0.5408*X + 0.9906), and the potentially negative correlation between the expression of hsa-miR-30b-5p and ALSFRS scores(r= - 0.2169，95% CI：-0.4524 - 0.04684，p=0.096), although they did not reach the statistical significance.


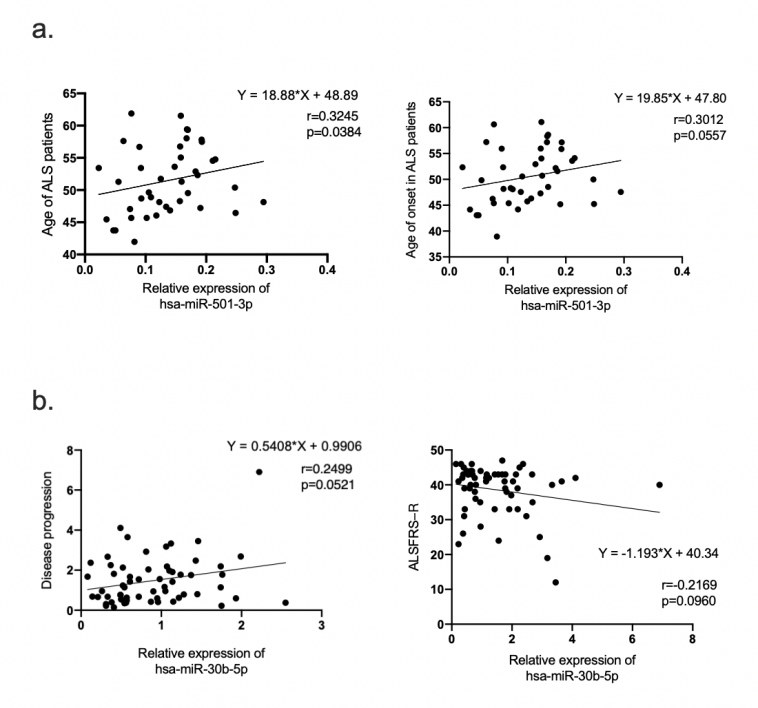


**Supplementary Figure 5:**

Venn diagrams of hsa-miR-34a-3p, hsa-miR-1306-3p, hsa-miR-199a-3p, hsa-miR-30b-5p and three potential statistical significantly miRNAs (hsa-miR-501-3p, hsa-miR-103a-2-5p and hsa-miR-181d-5p) predicting target genes.


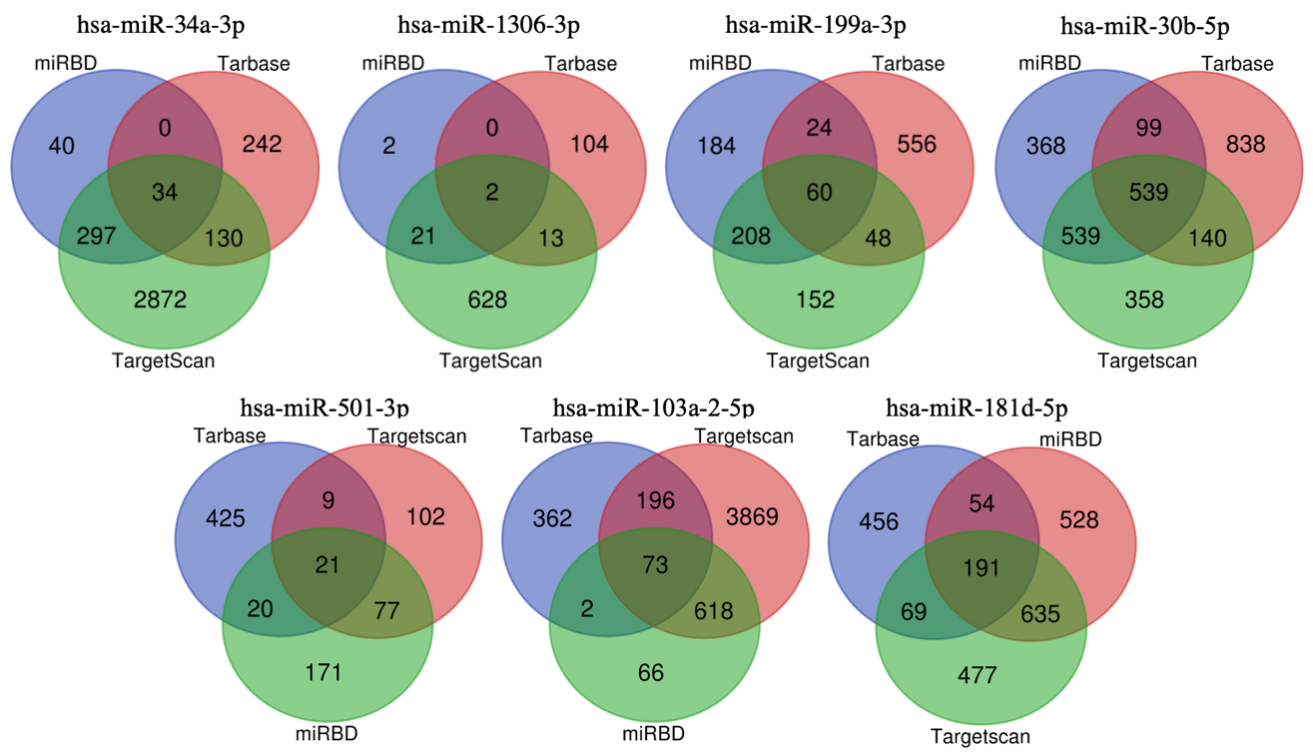


**Supplementary Figure 6:** The functional analysis of hsa-miR-1306-3p including GO and KEGG analysis.


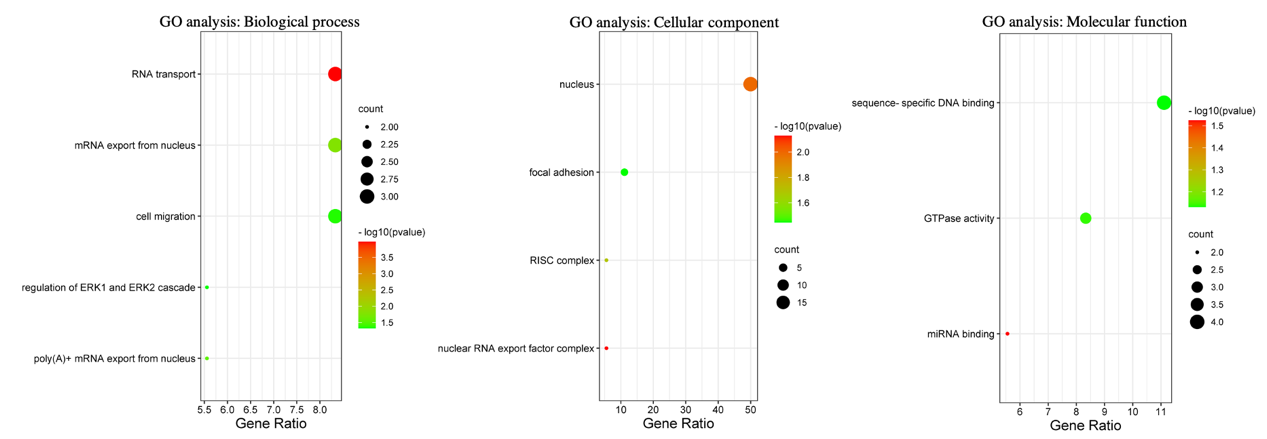


Since the number of hsa-miR-1306-3p prediction genes was small, KEGG pathways could not be carried out

**Supplementary Figure 7:** The functional analysis of hsa-miR-199a-3p including GO and KEGG analysis.


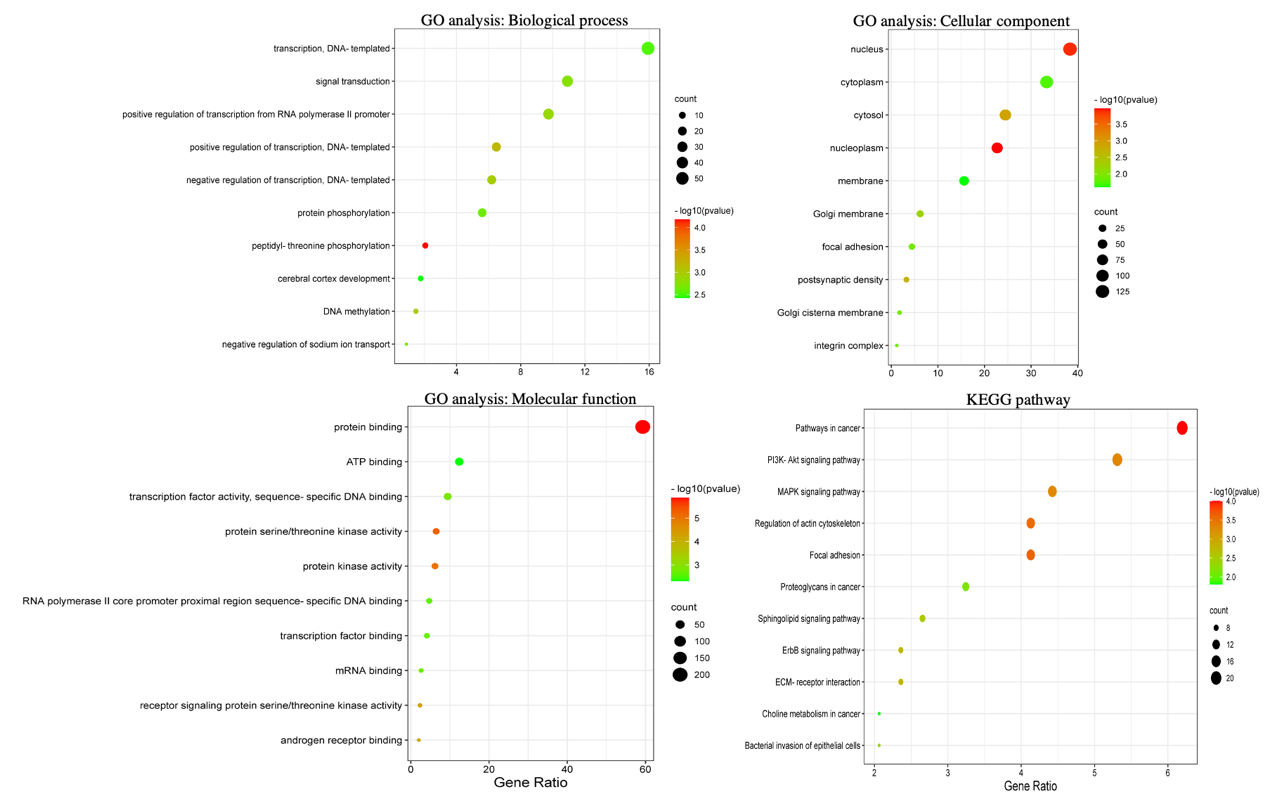


**Supplementary Figure 8:** The functional analysis of hsa-miR-30b-5p including GO and KEGG analysis.


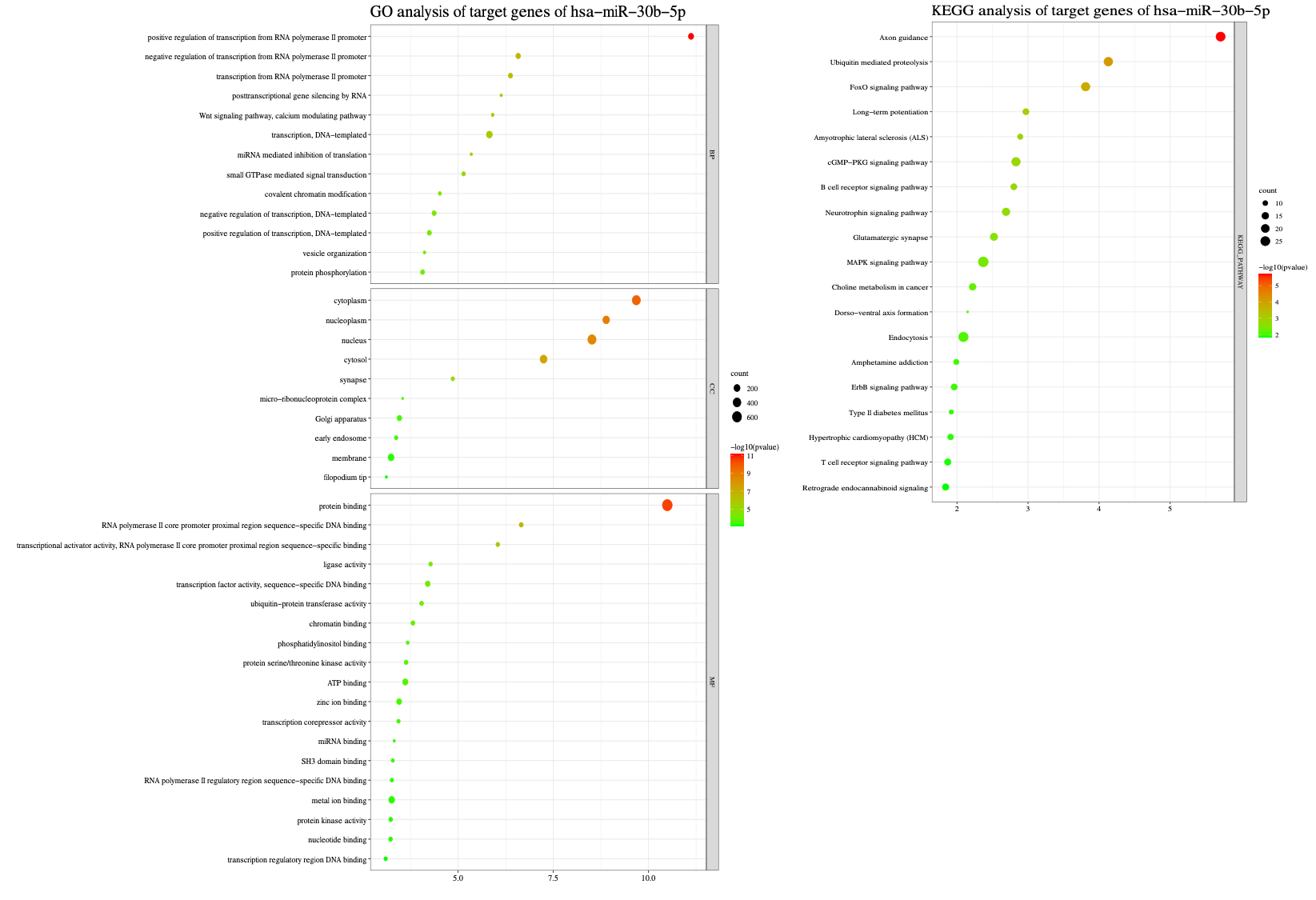


**Supplementary Figure 9:** The functional analysis of hsa-miR-501-3p including GO and KEGG analysis.


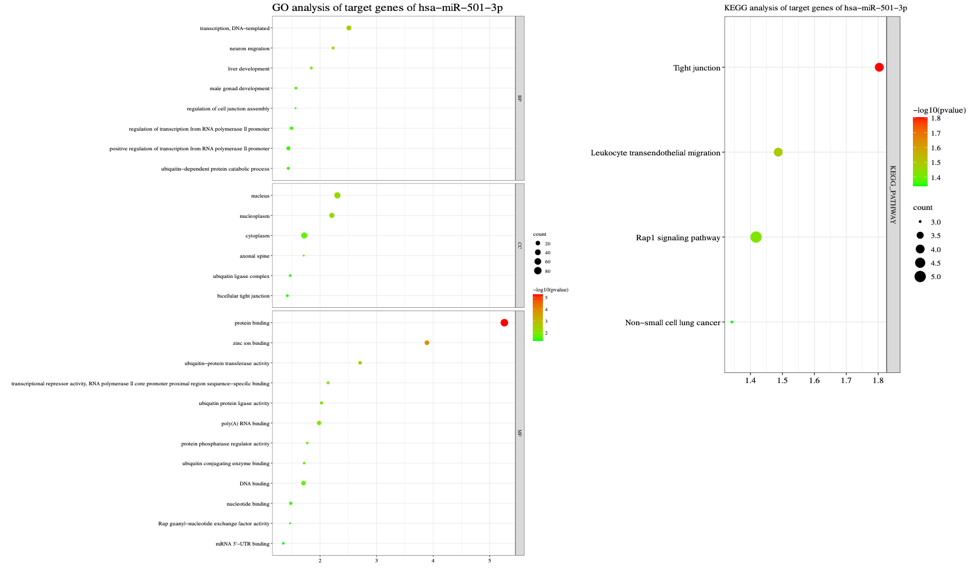


**Supplementary Figure 10:** The functional analysis of hsa-miR-103a-2-5p including GO and KEGG analysis.


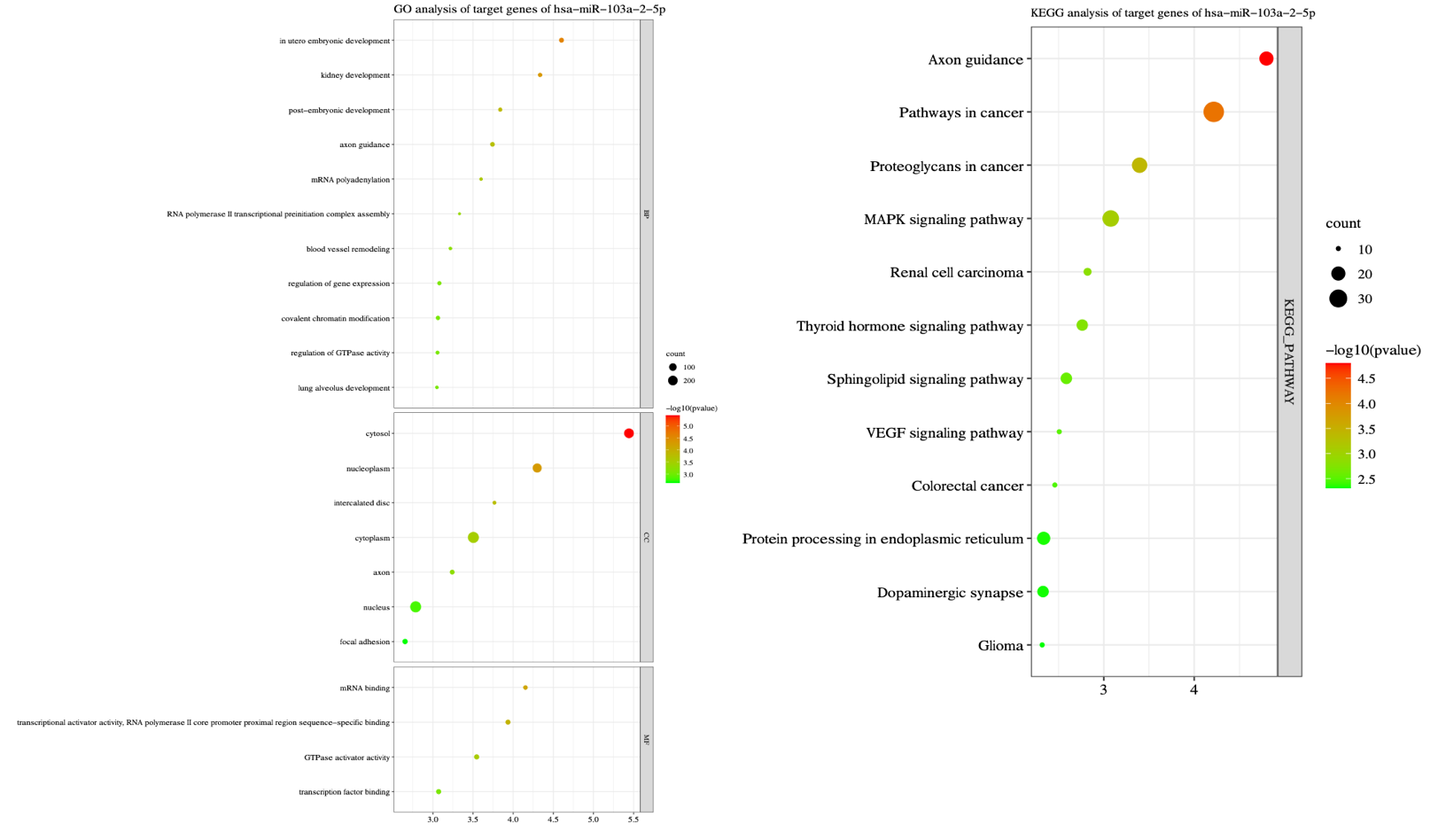


**Supplementary material Figure 11:** The functional analysis of hsa-miR-181d-5p including GO and KEGG analysis.


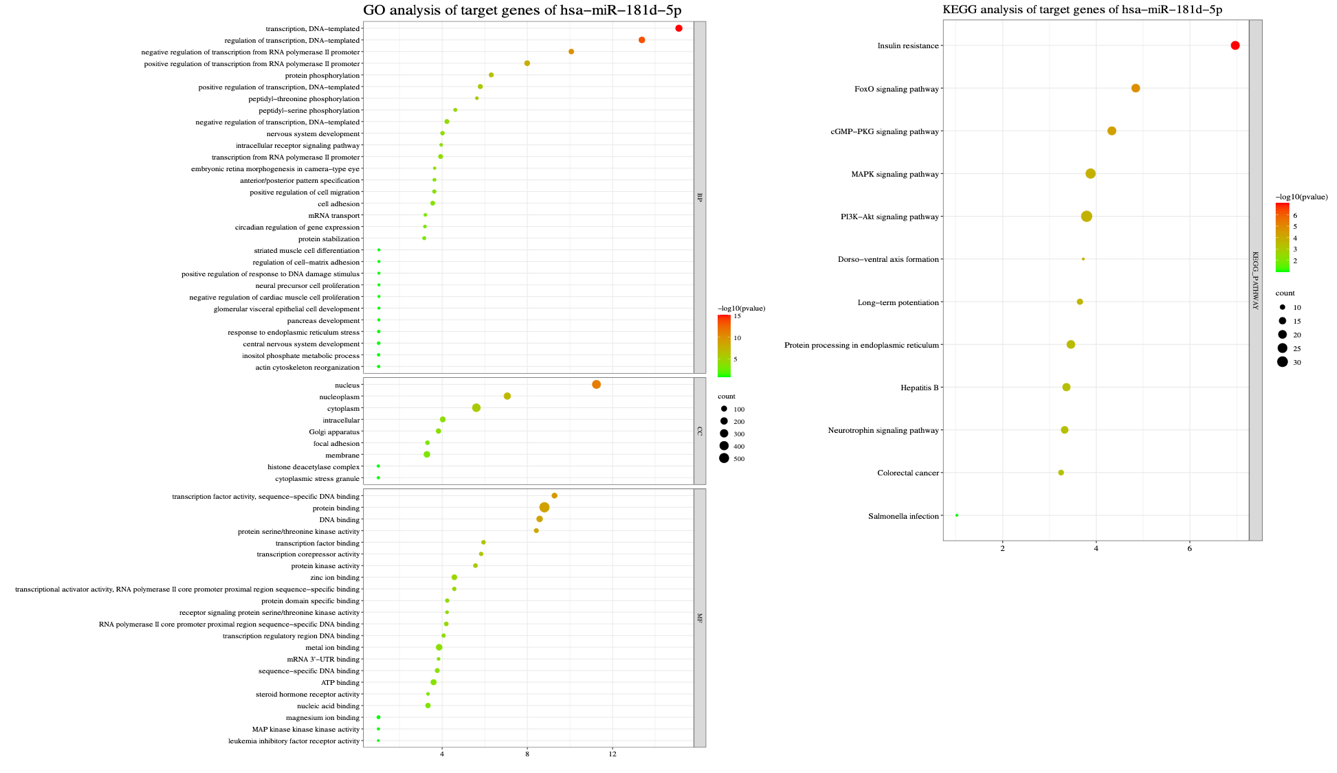


**Supplementary Figure 12：**

A graphical summary of four differentially expressed miRNAs’ KEGG network, which included hsa-miR-34a-3p, hsa-miR-30b-5p, hsa-miR-199a-3p, hsa-miR-103a-2-5p and hsa-miR-181d-5p.


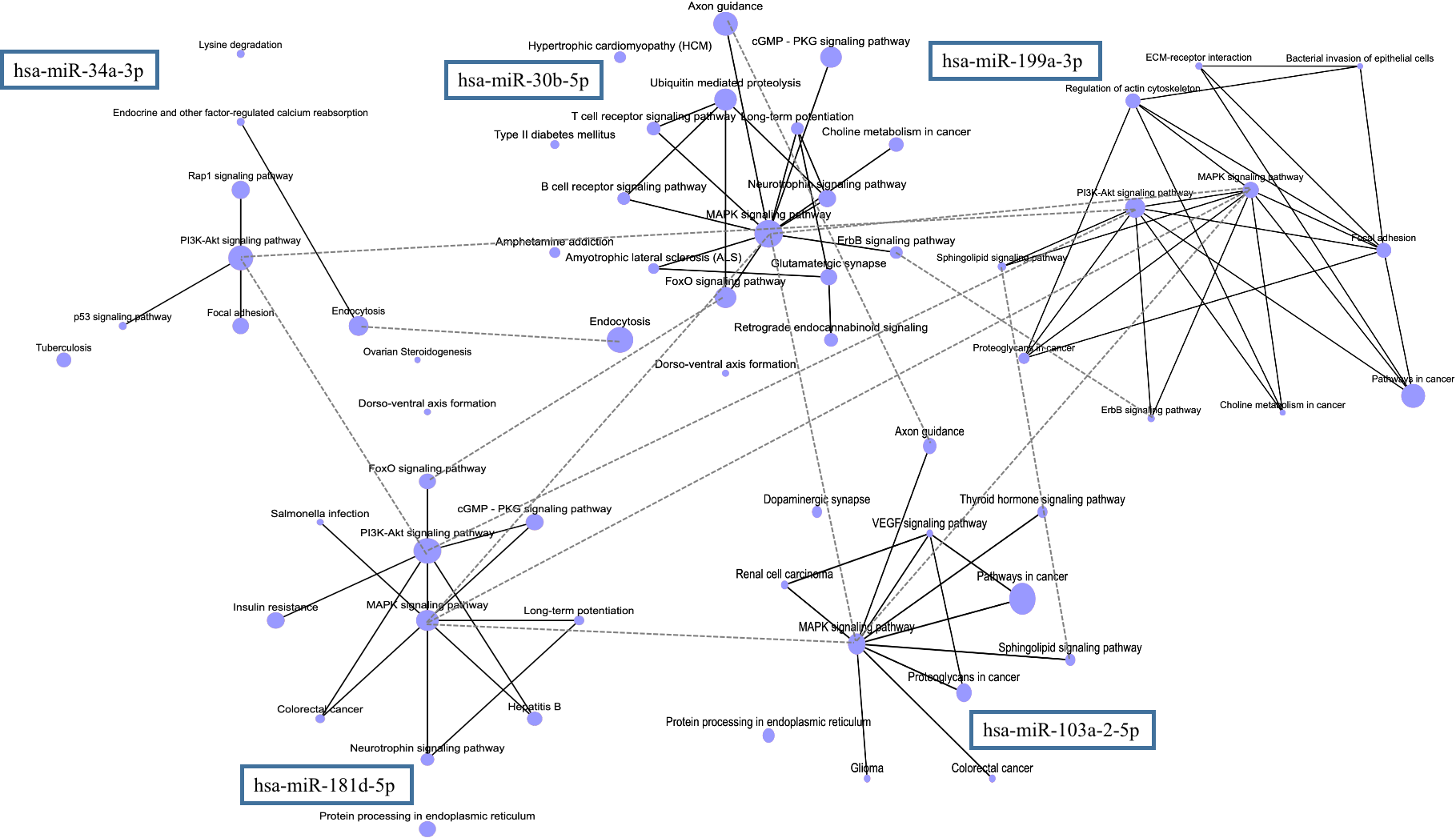


**Supplementary Text 1:**

The list of prediction of hsa-miR-34a-3p target genes of venn diagrams.

**Tarbase TargetScan miRBD: 34**

ECHS1 CAND1 ATXN7L3B OTUD4 HNRNPA0 NSD1 FAM122B SLC22A5 PDCD6 KDM5B FOXP2 AMMECR1L TBL1XR1 THBS1 SDE2 TET1 ZHX1 TMEM107 RBM25 NUFIP2 ARHGEF12 PFN2 PKP4 DUSP1 CLTC PTAR1 PLAG1 TGOLN2 CAB39 HOXA9 CADM1 OLA1 NCOA4 MIB1

**TargetScan miRBD: 297**

TACC2 SUMO1 ZEB1 G2E3 RAB3IP CASP6 IGSF3 SLK PALLD PARP16 A1CF GSS UTS2B RAD23B PODN CDK8 UGT2B10 KBTBD3 KL STPG1 FAM110C XRN2 RSRC1 LUC7L2 TRIP12 UQCC1 KDM2A SLC38A4 ATF1 CGA FGF1 UFL1 ZBTB20 GNB4 GORAB ZNF711 ZNF672 SNN PARG MAGEB16 YIPF6 BMP6 CUL4B TMEM170A TNF ELAVL2 FBXL20 FAM120A ATP6V0A2 ZNF561 GK5 CD93 MSR1 PDCD6IP MEX3D FASTKD2 ITGB8 TMC6 PTPN4 ELL CDH11 SLC31A2 LCE1C OIP5 PIGK PLS3 MRC1 SP4 CER1 LSAMP ANGPTL1 UBE2QL1 PNISR MYBL1 EMCN EIF5B TSPAN12 F13A1 KIDINS220 G3BP2 C5orf51 DMXL2 NETO1 PPFIA2 NR4A1 MXRA7 CLEC7A LRP8 AKAP12 TMC5 KLF5 MAPK8 TMEM167A STAR CAMSAP2 CRIPAK PRDM10 TMBIM6 LRP2 C11orf45 NCOA1 GUF1 SNRK EHMT1 RNPEP SYT11 YTHDF2 MAP3K4 PCDH17 SOX6 ZFYVE16 MED31 ABHD10 VASP PAXBP1 LURAP1L ILDR2 CPS1 SOSTDC1 GSG1 ADM STAMBP RAB1A MASP1 GFRA1 WARS2 ASF1A RFFL ADSS EIF3CL CERS6 ZC3H15 IFRD2 GALNT1 PLEKHA5 PDK3 WRB ACTG1 KRTAP13-1 RHOBTB1 TMC7 ACSL4 THEMIS TMEM176B RYBP C9 IFIT3 HMGB1 MOB3B IL2RG PLXNA3 USP30 GPHN RNF135 LPP MEF2D KMT2E APOBEC4 VCPIP1 MFSD6 CALB1 TRIM22 LRRC8C TRDN DDX27 ELOVL3 RBBP9 RBM33 ARMCX5 HRH4 ZFP37 EDC3 KIAA1143 HSD17B11 SH3GLB1 CXCR2 CLDN22 IRF6 PAPPA ANTXR2 RUNX1 ENAH PLXNC1 ASPH PRH2 TMEM47 ANKRD46 SNX4 ARHGEF35 ATG5 SPRED1 TMEM169 SCAI COL4A3BP MAN2A1 SPECC1 ALDH7A1 DSG1 NMT2 WRAP73 DDX46 GOPC LIX1 HSDL1 MIEF1 SEH1L CLASP2 EIF2AK3 FGF14 USP37 PSMA2 SPESP1 ZNF140 KCNK2 DENND5A RMI1 SHCBP1 SCYL2 SESN3 ZDHHC15 RORA MAOB ACADSB PRSS12 HIVEP2 MTG2 NPEPPS PHF6 PLXDC2 HNRNPF HCCS MXI1 PCDH19 HNRNPDL FUT9 GNAQ PDZD8 SASH3 PLEKHM1 RRM2 HNRNPAB WDR72 NLGN1 ATRIP STRBP LHX6 NAV1 SHISA9 HDAC2 DNAJB9 CTBS GPN1 EIF3C SCRN3 RYR2 LARP1B MAP2 FLRT2 FAM210A ARID4A XKR6 PPP2R2D C9orf170 INSR RNF212 RAD51D DYRK1A RAB11A CDV3 DESI2 CTPS2 YAP1 NAA25 ZNF330 FREM1 APLF DPAGT1 SERBP1 JUN KCNJ15 ATP2A2 ADAMTSL3 TXNDC17 SDCBP2 RPL37 KRT40 ZNF562 GHSR DNAH5 RNF44 TOB1 ATF7IP2 MDM4 CNST VPS13B JOSD2 PUM1

**Tarbase TargetScan: 130**

SLC7A6 LMAN1 C2orf49 NDUFS1 SOD2 EPT1 KIAA1919 EMC1 TMEM98 SPPL3 CAMTA1 FAXC RAB5B NEBL CNOT6 NR2C2 PALM2-      AKAP2 HNRNPUL2 LMO4 MOB1B GABBR1 CSRP1 CHAMP1 TMED7 TRAM2 ABCC12 RBM26 DDI2 XIAP PRKDC FKBP5 SIK2 ZC3H12C C6orf120 STAT1 QSOX2 IP6K2 FLCN VKORC1L1 MDM2 SPTY2D1 SEC22C MTMR4 ZNF624 CPSF6 TMEM181 TRIM11 GSPT1 ZBTB10 MTPN ZNF507 MSMO1 MKI67 DOPEY1 CALM1 CHPF CSNK2A1 ARL8B LBR ACTR3 RGMB GABARAP EXOC5 CLN8 STAG2 YTHDF3 ARL5B SGPL1 ATP13A3 RNF34 MAGI3 ITSN2 PPIG MID1 TOM1L2 PBRM1 UHMK1 COG2 DYNLL2 ARPP19 CACUL1 CCSAP DRAM1 NUP50 MCL1 TXNIP NUDT3 AGO1 CLDN12 DNM3 OGT DFFA DEDD BCL2L11 TATDN2 DIP2B HSPA9 ZNF451 SECISBP2L MED1 IGF1R SF3B1 ELF2 MED28 DST KMT2A RAB12 MAP1B BACH1 DSEL RHOBTB3 CANX CCND2 SSX2IP DUSP16 CBX5 FBXL18 PEG10 PEX5L MGEA5 BCAT1 LARP4B TBC1D9 TNR MISP PDGFC DPY19L1 APP IGF2BP1 DNAJC10

**miRBD: 40**

CDC14A MAP3K13 SPX DEPDC5 DEUP1 FBXO3 ZNF480 TMEFF2 ADGRF5 44531 NTRK2 LNPK SINHCAF SELENOI TBC1D7 LAMP2 NEXMIF PLPPR4 FMC1-LUC7L2 ENPP3 AASDH ZNF407 TMEM74 PCNX4 CXorf40A CTNNA3 TCAIM PDCD2 STN1 OOSP2 PPDPFL GNPDA2 CORIN XBP1 FBXO4 FAM122C ELP1 BCL11A ADGRF4 CIART

**Tarbase: 242**

KCNMA1 PYROXD2 IFT52 BTG2 SLC25A3 PXYLP1 G3BP1 CHD8 UBB OSBPL8 PI4KB KPNA2 TSPAN13 CSNK2B-LY6G5B--991 YWHAQ ENOSF1 CPEB3 PCBP4 BCL2 SON IL24 ERLIN2 TOMM34 FUS CELF1 SNAPC1 IRF3 H3F3B SLC35A5 EPHA4 ARAP1 MED14 XPC MTR ARMC6 ASH1L C7orf60 RER1 RBM7 NSMF BICD1 EIF5A EHD2 CTNNB1 UBE2J1 NCSTN PCDH15 NOL4L ENPP2 USMG5 RPS4Y1 SMG1 RALB KIF1B TNRC6B KIAA1109 ARMCX3 MAGEF1 DCTN4 RBM4B NUCKS1 EFNA5 ZIC5 TFAM C6ORF174 USP15 CHD7 DPM1 CLIC4 TMEM70 CXCL2 DZIP1 MCM7 NDUFV1 GLT8D1 HSPA14 TCEB1 IARS PDE5A RPL3 RBBP7 PSMB4 NOP14 SLC35D1 CANT1 POLR2A TWIST2 ADAMTS3 BRMS1L MAFG TGFBR3 PDIA6 SRPR DGKE PLEKHJ1 MRPS25 CD47 ELMSAN1 SEPW1 FNDC3B NUP205 RPL23A C5orf22 IGF2R RPL31 TOP2B PSMA6 PAGR1 ZNF91 NCOA7 CYBRD1 MTCH2 ATXN2L LPCAT1 PEBP1 MSANTD4 EEA1 SIK1 ARID1A SMIM13 ZNF850 LYPLAL1 PHF23 SLC19A2 MAST2 ITIH5 GK FASN BTNL9 GPNMB MAN2A2 FBN2 NEDD4 YWHAE KIF11 EFNA4 ASF1B LARS CPSF7 HSPA4 TUSC2 RNF103 CHCHD7 SYNGR2 KDM1A LIMA1 MORF4L2 FBLN1 PPHLN1 AFF4 TCP11L1 HOGA1 VIM SUMO3 ITGAV KIF21A EDNRB STXBP5 ZSWIM8 USP25 SPEN MRPS22 TGFBR1 LAT2 AMDHD2 RND3 PRDX5 LENG8 CD46 ILF3 CSNK1A1 KPNA5 ABHD17C HEATR1 HSP90AA1 RNF213 SIAH1 VDR SEC23A IRF4 CPNE8 SIGIRR GRB10 SLC11A2 LATS2 SLU7 ZYG11B SERTAD3 PNRC2 CEP78 SERPINE2 SLC36A1 LAMB1 SMARCD2 IRF2BP1 AP1G2 MAP3K2 MBNL1 HSPA5 APH1A TP53INP1 XPO1 DDX6 RAB29 SLC20A1 TSG101 SHROOM3 PCDH10 TMEM109 ASNS VAMP3 RELA STOML2 ACTB SUGP2 ZC3H11A BRWD3 MDN1 TMEM30A GPRIN3 FAM114A1 ZBTB44 CERS2 HDGF GMPS EIF4G2 ZNFX1 GATAD2B FMNL2 KPNA6 DDX3X CASK WAC TRAF3IP1 HIPK1 SEMA3C PPWD1 KBTBD7 PATL1 GNL2 SNX17 KCNA7

**TargetScan: 2872**

C5orf42 CLMP RPS11 SLC18A1 PLCE1 FARP1 SAMD4A HAUS2 FAM106A FIBIN GRAP2 APBB2 C10orf25 CNBP PDHA1 VPS4A ADAM12 CRTAM AC117395.1 CXCL3 NT5DC1 LRRC6 PLA2G2C DCAF5 DLEU7 GNAL CCNI MITF RDH10 RTN1 KLHL14 CSTF1 BTN2A1 DDX5 HOXD11 IMP4 KBTBD8 ZSCAN9 1-Dec SLC33A1 PROP1 DCLK3 ZNF131 TRIM33 TMX4 CBWD7 PPP1R13B HCN1 AL355390.1 MIER1 CCDC97 KCNK3 C1orf27 MSRB3 KCNK6 XK SLC4A8 SLC15A2 MRPL45 CHRM5 ING3 TET3 NINL TMEM39A PCSK1 WSCD1 MAX NR4A2 IPO9 ARF6 RRP7A EVC ANP32E LY6K HOXA13 AC115618.1 SNX33 ARL6IP1 FADS1 TSC22D1 DKK2 RPS6KA5 ZNF182 CACNG5 POTED TP53AIP1 DIP2A CCL22 ARL4A TANGO2 RIOK1 ZNF48 GEN1 BRCA1 UNC45B ZNF510 TMEM180 WDR89 HNMT CLCC1 ACSM3 C16orf98 NOL8 CDK20 COL9A2 USP6NL ABCD4 RWDD2A SNX16 GPC5 TBL3 BBS12 LRGUK AGBL4 SLC25A46 INTS6 ZCCHC5 SEC14L5 SLC8A1 PDCD5 CLDN16 AP1S2 ASB13 RAB33B CECR2 C1QTNF3 AC106017.1 TEKT1 RNF103-CHMP3 KPNA3 KCNA1 CD1A SUPT4H1 SRRM4 FAM73A SSTR2 CLN5 PPP3CA ZNF529 ALDH3A2 RAPGEF6 GNAS LGI2 SHROOM1 GHR STARD5 BCLAF1 TRA2B EXOC3 STK17A PPIL4 KIAA1715 ZNF345 SERPINB9 KLK8 CCNY WDR59 EVI2A GTF3C3 EXOSC7 RBPJ WHSC1L1 LRRC8D FOXM1 TRUB2 CEP170 MAOA DNAJC5 ZNF280C GATA6 ANGPT4 MSH6 DSTN RASGRF1 FBXO31 PSMG4 ST8SIA4 RFXAP CR769776.1 GOLGA6L10 IP6K1 EMP2 FIGF GJB1 TPM3 SLC6A15 EPB42 FAM19A4 POLR1E FRMD5 CDK1 FRRS1 N4BP2L2 BTN3A1 RNF217 TRIM2 OPHN1 ADH5 PLEKHG7 HS2ST1 RFX3 HNRNPR EIF1AX PPM1A TMEM120B PCDH7 SLC25A35 ORC1 TMEM156 ZNF239 SLC36A3 FAM198A KIAA1324 ABHD2 FAM162B TRIM29 NUP37 PXDN RHEB CDS1 PPP1R9A CDCA4 AVEN ZNF35 CARD8 C14orf132 ADAM19 ZSCAN4 CLCN4 CHST11 DNAL4 ASS1 SLC9A9 DAO SEC23IP RCSD1 ZNF331 ADAM23 B3GALT5 HMGN4 RICTOR RAG1 EIF3M SRP72 ZNF24 ZNF334 PIP4K2B TLCD2 NME1-NME2 DDHD2 CRIPT BDH2 GRIK2 PLD6 PHLDA1 BACE2 LIF KDM5A ANKRD49 ZNF395 ABI2 CACNB1 AFAP1L1 KDM6A NCAM1 ZNF549 SLC25A21 AC012360.2 CLSTN2 TMEM33 ARIH1 POLDIP3 DAPP1 LRRC10 TMEM125 C12orf4 CDC6 PLD1 COL7A1 GLG1 CIITA AKNA PKHD1 CRP L2HGDH RP9 PSG3 TMEM11 UBXN7 LYSMD2 CDH8 PIGP PCCB TPBG LZTFL1 UGT2B4 WDR12 KLHL7 HOXD13 DCUN1D5 TCF4 ANKLE2 AGTPBP1 NBPF3 OSBP NKTR GALNT18 CEP85 NAALADL2 RBM44 ASAH2 RHOBTB2 SMTNL2 ZNF432 ASAH1 UTP14C LONRF2 COLCA1 AP3B1 BST1 NFYA NRG4 IL5RA STON1 DEFB125 PERP SMC6 PBX1 GOLGA8N RSU1 MYLK2 RPL4 PPP1R12A RSPO2 VAMP4 UBE2N SORBS2 GLYATL2 EML5 MUC15 SPINK5 ACVR2A PRIM2 ACAD11 PRRX1 PGM2L1 OPA3 ZNF805 RPS6KA6 STRC SYTL5 PRND MAGOHB IDS BCL2L15 HUS1 OLFML2A TMEM140 WNT3A AC005606.1 SOWAHB ZC3H6 C17orf77 ASAP2 DISC1 SFRP2 FAM71F1 ACOT2 KIN PPARGC1A DUS4L KCNJ6 VIT ASXL2 ZNF738 ZNF891 CCDC6 NCOA3 ZNF146 DOK4 RASSF4 ENTPD7 WDR96 TPT1 SEC63 CCDC113 SLC35F1 SYTL4 VSTM4 FOXL1 BCKDK DNAJC9 ITSN1 BOD1 CMYA5 KIAA1161 TNKS2 CXorf23 TMEM241 FANCA TTC3 DIXDC1 SEMA3G ZNF354C OR2H1 TCF21 MEIS1 GJA9 NGFRAP1 LRRC2 GLDN SLA WDR91 EGF PAQR3 ANKRD26 NHLH2 CPVL RGS20 NBPF16 DKFZP667F0711 BRCC3 HIATL2 ERCC1 VWCE YPEL5 TIGD2 KRT77 PREX2 ASTN2 PI15 FAM177A1 PROSER2 KLF2 KIAA0930 TNFAIP8L3 RNF7 TANC2 FAM161A SNX13 CPD DCT RCAN1 DNAJB4 TMEM189 CNTNAP3B EPHA7 PDE6H LMAN2L FLJ00388 C1GALT1C1 TYRP1 DSE SCN4B OSBPL3 PIGG RP11-366L20.2 ZNF264 AC069547.2 AKAP2 TCEANC CD8A GTF2H5 ISCU NSG2 LRR1 XAF1 MSANTD3 PLA2G4C ABCG8 ZNF177 MIP ZNF442 XPO4 RP1-170O19.20 EPS15 FDX1 FAM19A1 SH3BGRL2 TRPA1 TRABD2B GNS WIPI2 TCTA PEX26 PTGS2 ACBD7 CCDC90B LPPR4 UBE2D3 DAGLB NRAP PPFIBP1 FAM49A NEGR1 ARFGEF2 KIAA1551 C17orf80 ZNF614 ANKHD1-EIF4EBP3 PARD6G COX7B NAV2 TMEM185B MICA SLMO2 GRPEL2 C15orf38 REPS2 HAS2 FAM78A PRDM6 RNF150 DNMT3A FOXN2 GAD2 ITGA6 C1GALT1 NKAP SC5D NOTCH2 IMPA2 ZFP90 FAM122A SLC30A7 PSMD12 RASSF9 TAL2 NET1 CCDC144A ESR1 ACLY SYT7 PGGT1B LRRC19 GABRG3 SURF4 CHCHD5 BCAS2 NFAT5 NUDCD2 GIGYF2 CHMP3 CMKLR1 EDA2R TNFRSF19 PLCXD3 NAP1L6 FAM107B ECHDC3 LUZP2 PTPN14 KCNAB1 RBM3 PP13439 DMTF1 ANKRD40 BMS1 PODXL GRM1 ACOT9 PDE4D MUC20 FAM53A ZNF551 RABAC1 SLC48A1 ZFHX2 ARHGAP26 EPSTI1 KCNMB3 JAGN1 NPNT AC022532.1 PDE3A PCDH11X ITGB2 DMGDH PCBD2 NTPCR RIMS1 RCN1 WSB1 CDKN2B DRP2 TXNL4A SRRD CLLU1 PDZD7 DDIT4L PAK1IP1 PARVB ABRACL FAM96A FAM89A MFAP3L FHL5 ZBTB41 AL031666.2 CDS2 DCUN1D2 MME GOLGA6L9 RP5-1052I5.2 FAM213A SHANK3 ZIC3 EEF2K CEP72 SLC5A3 WDR26 ATP6V1E1 C3orf70 SPTLC3 UNKL ART4 EVX1 KIAA1841 TMEM53 MALL C1orf180 ACPL2 RSPH3 SPIC PIANP RPS24 KLF9 ISCA1 IL1RAPL1 RPL24 NLRP9 KNOP1 PCSK9 C6orf57 KIAA0087 NKX2-1 GBX2 LAMA3 SDHAF1 FAM120AOS COCH APOBEC3F ARID2 AC124890.1 RGS18 SLC11A1 ASAH2B HUNK GNA13 GPD1L B4GALT4 TSPAN14 CBLN2 PDK4 PANK3 CNKSR2 FAM211A AGA NEK10 ZC2HC1A ZNF74 ADCYAP1R1 SAMD5 LRRFIP1 RBMXL1 POLR2M SERPINA4 FKTN IKBKAP EPHB1 COLEC12 CDCA5 SLIT1 LPHN3 REEP1 UBE2T MYOCD GALNT10 SETD3 C9orf156 ELK4 ATP2A3 GPR110 ACOX3 WEE1 NR2F2 SSFA2 GOLGA8J RGS1 FHOD3 FAM13A ACER3 ZNF862 AC104841.2 TDP1 AVL9 TIMM8A RAD21L1 TRIM24 AC004899.1 AJAP1 WIBG RP11-422N16.3 ECT2 SLC25A42 DEPDC1 7-Sep PRSS42 OLFM2 B3GALT2 RP11-455G16.1 VWC2L ABCC9 CDK18 SNAP29 HFM1 KDM4A PHAX HLCS GPR155 GCSAM RAB24 RWDD2B GREM2 HDDC2 VPS26B CACNA1C VASH2 ZNF676 FIG4 IFFO1 CDC27 UBR1 PTTG1IP VPS35 BRI3BP DBP LMO7 PAM TMEM178B WDTC1 TTC19 UBASH3B OGFRL1 ARHGAP32 CITED2 C12orf49 KPNA4 GGACT MRPS10 FAM126A TRPM7 CECR1 RABIF TMED3 IFNE PPIL3 NEIL2 EIF4E RCOR1 CDRT1 ZNF528 SMAD2 CCS YWHAG TMEM68 ATP5J2 HEATR5A IMPG2 SLC29A4 AC007375.1 FOXJ3 MRAS WDR70 CBWD1 YAE1D1 KRT80 SHE PPP5D1 ALG10B GIPC2 OGN C6orf89 EMC2 SRSF7 C9orf72 NAMPT PCNXL4 SLC5A7 ERCC4 FAM184B SLC31A1 ABCA10 EXD1 TLN2 ZNF493 FAM83B FLJ30594 GDNF IRAK2 BLMH MYO9A FOXN3 ADCYAP1 TMEM182 PFKFB2 KIAA1644 SH3TC2 MAPK1 PAK7 HN1L KCNS2 PYGB HOXD12 NHLRC2 ANKH AC011997.1 TNRC6A FTO SAMHD1 OCLN EPC2 SERTAD4 SPRY1 DDI1 MYSM1 STIM2 GYS2 NUPL2 SF3A1 C1QL2 COA4 TAP2 RPGRIP1L THSD7A IPPK BLVRA ICMT LHX4 AKT2 ZDHHC3 IKBKB BTN2A2 TMED8 PIP4K2A MRPL46 PLGLB2 MAPKAPK3 B4GALNT3 AFAP1 TMEM136 SIRT5 FXYD5 GPR161 TRIM66 ZNF317 EMC10 TTC39A CBWD6 GLMN PCNP SIRPA CSGALNACT1 TRIM52 ZNF786 METTL21B LYVE1 CREB1 FANCM TSC22D2 NAA38 TRAK2 C14orf37 CEACAM5 POLDIP2 RAB3GAP2 RIOK2 CFL2 C4orf32 PDLIM4 LRRTM2 STAG1 UPB1 SLC22A15 FUT3 AC127496.1 CCPG1 SORCS3 ABCF1 ANKHD1 PDP2 VPS37A SELPLG BAZ2B PRG2 TGFBRAP1 POU6F2 GCOM1 NFE2L3 PSD4 OPCML EYA3 KRT38 OBFC1 TTC39B HSD3B2 GNE MGAT4C WDR77 KIAA1257 CEP85L FKBP3 PLEKHB2 IPCEF1 TUSC3 CCNT1 MPP6 LIPG PHACTR2 SZT2 EVI2B MRC1L1 TNFSF8 ARHGAP29 TMEM251 PAOX CDR1 ZNF541 KCNJ12 GOLGA8K MFN1 RTCA CPEB4 SCN1B SNX11 RNASEH2B NIF3L1 ESCO1 TMEM52B MTAP C1orf65 RFC1 HCFC2 FAM206A CMAS FAM175B TXNRD1 SLC30A1 SRGAP1 TNFRSF21 SP2 LILRA1 TMEM38B CHRFAM7A KAL1 CWC22 ELMO1 ZNF423 CYTIP MTDH SCIMP BARHL1 RAD50 TRIOBP KCNJ3 GPR113 MBTPS2 ZNF573 RHOT2 EIF4EBP1 APLN UVSSA GTF2B NKX6-2 FER PATE1 CDK7 UTP6 GSDMB COPS3 PMPCB TMEM200C MACC1 MED21 KCMF1 ZDHHC13 GGCX SPTBN2 ZNF585A MCC CAMK4 RAB22A KBTBD4 DEFB118 PJA1 TMEM189-UBE2V1 ARHGAP5 FGF5 RB1 VWC2 MTO1 GRIK3 GPX8 NUMB CELF2 CCDC85C ALG8 SHOC2 PDHX SRC MRPL27 MINA CLCN3 SETD1B METTL24 XRN1 NABP1 NR0B2 GPAM SSR1 GOLGA6L4 SETD9 C17orf102 HEATR6 VSTM2A PGR RP13-996F3.5 ZNF804B CSNK1G1 MBNL3 NR2F6 ZBTB8B DLGAP5 PHEX TCTE1 LUC7L3 SNF8 MECOM HAT1 POLH C11orf74 PLEKHA2 LRRC1 PKIA ESCO2 SCN7A SEC62 NKPD1 ESRP1 SEC14L6 RIC8B NADK SBDS SGSH HOXD3 GIPC3 ZMYM4 GPR85 P4HA1 SP3 CBWD2 RAP2A ZNF532 LRRC20 TYR ZNF106 RP11-664D7.4 LCP2 GAB3 MB21D2 FBLN7 NR2C1 VCAN SLC25A10 TMEM64 OSBPL2 ABHD17B GLS SGK494 CNTNAP5 KANK2 SHROOM4 DDHD1 ALDH1A3 CCDC69 SHPK BICD2 NUDT13 ZNF560 TM4SF1 PLGLB1 C10orf131 CDH7 GPR150 ENTPD1 TMEM41A GDE1 RAPGEF5 POTEI C3orf58 FAM124A PLGRKT CHIC1 ERVMER34-1 SULF1 PRDM5 UBE2E2 FAM71F2 KIF3A RPS27L CDCA2 MCUR1 ZMYM2 EHD1 UPRT MIPOL1 PPIP5K2 C2orf15 ZNF550 SPIN3 SH2D4B UNC5D TIRAP CD84 APH1B RASA1 TBC1D15 SMCO3 EVA1A UBE2D4 ITCH KLHL42 MBP ZCCHC13 GJC1 TANGO6 HIST1H4H C20orf194 ATP10D WDR48 NRXN3 KIAA0226 MAF CYB5D1 FAM210B RASSF8 DOK5 HELZ TRAK1 USP12 AGPAT5 PTRH2 ZNF668 RHPN2 HINFP RABGAP1L FPR2 KLHL5 SRM PPIC ANKRD33B ABCB11 SPRY4 C9orf40 FZD3 KCNC2 SOCS5 MXD1 ASXL1 TMIE ATP5L2 HADHA TBRG4 TIMM50 PER1 IMP3 ZFYVE27 LMOD2 CNIH1 ATG12 SNX12 MTCP1 JAKMIP3 PCTP MRPL42 VRK3 SIRT1 RPAP2 ATRNL1 CAV2 MIA3 SPHKAP TMEM135 ARHGAP35 BCL6B CHRM3 NEURL1B PREX1 TBX22 TRIM46 SNX15 HPSE ADNP MMADHC 11-Sep INPP4A SLCO5A1 UBE2V1 AFTPH SNRPB2 AIG1 ST6GAL1 ZNF418 LARP4 ZMPSTE24 SNTB1 KLHL11 HMGCS1 UBE3A C9orf169 LRRC27 MT1A PYGO1 RPL10L CTC-432M15.3 PIGU ZBTB3 ZNF570 NCAN AC008948.1 EIF1AD CTSO ORC4 MARS2 SFMBT1 RSPO3 ZNF207 KLF13 GOLGA8R GAK TOLLIP DNAL1 HSF5 AP5S1 PSPC1 PFKFB4 PCDHB5 IDE RNF11 CLEC12B COL11A1 UCP3 DEXI TXLNB IFNAR2 CYB5A NBPF14 SPRED2 CYP8B1 PITPNC1 LPIN1 RSF1 MKS1 CX3CR1 POU3F3 MYLK3 LIN7C WDR13 COX7C CNNM2 NXPE3 CCDC152 ELK3 INADL GOLGA8G RRP15 PTCHD1 PSG11 P2RX7 RSL1D1 STX6 CDC42EP1 B4GALT5 ZBTB25 MACROD2 AKAP11 FBXO28 MCMBP CCER1 NOXRED1 PLAC1L KRBA1 WDR3 TMTC1 TSHR XKR4 ETV1 MOCS2 ADAM11 CDC20B WFDC6 CTTNBP2NL RGS7BP PARP9 ROR1 SYNM FSD1L LPHN2 PPARGC1B MRPL30 PTDSS2 PLEKHA6 KDSR GLP2R MCM8 HNRNPU TMEM56 NME1 BMP5 C20orf96 FAM101B RFC5 MAP7D1 TWIST1 HEATR2 RBM28 MTFR1L AAK1 RPL14 FBXO2 AOX1 JRKL GMEB1 TMEM26 CRB1 KLK3 LTBP3 POLA2 NOL10 OTOG PHKB EDEM3 TBC1D8B ZNF776 FAM45A SF3B3 RBAK RANBP9 LAPTM4B USP8 EVL NUDT21 LNX1 C4orf46 SYT4 SERPINA10 KCNE4 RBM48 RCN2 TRMT5 C18orf21 KAT2B CYP1B1 MEDAG AMER1 PDLIM5 MAP3K9 BTAF1 AC013269.5 BRWD1 AC008443.1 RHBDD1 ZNF445 AGO3 PGBD4 STT3B PSD3 ISCA2 AP1AR AGR2 FNBP1 EYA2 C15orf61 DCAF17 CHRNA7 CKAP4 FANCD2 SLFN12L ARL6 HECW1 MEF2C MCTP1 UCK2 PSPH CBFA2T3 PGK1 AFF3 DRD1 NBPF24 KLF3 MLXIP FZR1 XRCC2 GMPR2 B3GNT5 TBC1D19 PTGER3 CLPB DONSON ERGIC1 USP29 KIF26B ZNF416 OTUD1 HECA EIF4EBP2 DFFB TNIK DIO3 NBN BID IRS4 PANX1 FGL2 COX5A SOS1 ARHGAP11A MPZL2 LAMA2 EHF TOMM20 ZDHHC2 PTEN AFF1 GAL3ST3 TBC1D14 RAP1A ERI1 TC2N ATXN7L1 ZNF385D S100P ZNF783 PACSIN2 WEE2 ZNF473 C8A RFX5 FAM127C RDH13 CSNK1A1L CLGN SPTLC1 HLTF FAM109A AP1G1 CTNNBL1 KLF11 CLHC1 C9orf85 NDUFAF3 GPR180 GNAI3 NETO2 PHLDA3 C7orf55-LUC7L2 C14orf23 NDST1 TRIM23 ZNF248 PTPRE DPYSL2 SSH2 SYNPO2 C22orf46 ZNF230 THOC5 DNAJB1 KIAA0141 PTPLAD1 MYO3B USP20 DUSP22 BPIFA2 MCRS1 ZNF347 TPP2 GPR3 MDGA2 RC3H1 MED29 CCDC50 ATAD2B MRPS14 GPR116 PHLPP2 SFXN2 DIRC2 KIAA2022 TTF2 CD33 FGFBP3 SLC39A14 NXPH3 TIGD6 NGRN CDK15 DDX19B EXT2 TUBAL3 NOM1 CRY2 GPR137B MECP2 DIP2C AL020996.1 ARHGEF7 NOS1 BMPR1A SNIP1 TMEM86A MPPE1 MOB3C ZNF704 MAML2 RP11-159G9.5 CLCN6 ABHD4 SAMD12 10-Sep PLN SLC35F6 MTHFR PRDM9 SCOC INTU KCND1 PTPN11 YOD1 NUP93 KDM5C ZNF274 TBX5 TBC1D12 FBXO22 UCHL1 TTLL13 DGKH ZNF581 CDK12 APOL6 ASB8 PLCG1 HTR2C CARD18 ST3GAL1 RFTN1 RASL10B NEK4 MRPL19 PPP3R2 DICER1 CACNA1E KLHL23 APOC3 SPATA17 RPL7L1 ZNF148 PGRMC2 OSTF1 CAPZA2 IFNA2 ZNF749 ZNF321P C14orf28 ANGPTL3 SEC16B CYFIP1 GPR173 MFAP3 RAP1GAP2 GAN EXOC6B SRI HDAC5 SLC25A32 LMLN PELO FAS AFG3L2 SWAP70 NUP62CL BTN3A2 ADAT2 CNKSR3 REEP3 BTBD17 HBEGF OSMR ZNF574 BTRC EIF4EBP3 CTD-2368P22.1 SLC25A16 TFCP2L1 COMMD2 WIZ ACBD5 SEMA3E FAM105B CHEK1 DTX3L LRRC58 EXD2 SCIN MBOAT1 TRIM72 PARVA SLC40A1 MRAP2 PHC3 MYO5B PKLR C3AR1 FOPNL TLX2 MRPS17 NUP153 ZNF117 NKRF ANAPC13 TMEM233 TTYH3 FANCL TNFSF15 GABRR2 RNF180 PAG1 PIK3C3 C12orf76 KCTD18 DCLK1 ZNF665 PACRGL SPOPL RAB14 PIGV TRAF3 TMEM237 CAMK2D ALG14 USP10 C1orf123 AC007390.5 TMEM161B WDR75 GCM2 FKBP14 FGD4 FAM135B NPAT FRAT1 ZNF81 APMAP CDKL4 PDXK PIGR LRRC4 ZNF670 RCOR3 NRIP1 CS BNIP2 IFFO2 ZNF609 FCRL3 FGB FUT6 PTPRB UFD1L ITGA1 CCDC83 TMCC2 TCF12 HACE1 CATSPERG SIPA1L2 CCNG2 ZNF641 CDX2 NECAP2 LSG1 PRRG4 ACTR1A MTMR8 FBRSL1 MERTK ARMC8 SLC47A1 PCDHB11 CCRN4L RNF144A CBR4 EVI5 DCTN3 POC5 RP11-192H23.4 SMC1A ZWILCH GRIK4 IFT80 MPV17L DTX1 RGAG4 GNB1L C11orf58 RET MAPT MRE11A PRKAA1 HOXD4 ZNF527 TRIM55 CCDC104 PRSS35 GABRG1 RASEF NUPL1 RPL22L1 MAPK9 KCTD5 PPTC7 SLC12A2 ESRRG FBXW2 CNGA1 EFNB3 CHRNB4 ZDHHC24 OGG1 STON2 CSTF2T WIPF3 C1orf51 EPB41L1 DNAJC3 PTGFR IREB2 RAB21 GRSF1 CDR2 SELE MSX2 AGAP9 ABCB1 KIAA1549L ZFP36L2 TADA3 TRAT1 USP9X FAM188A VAPA IFIT2 DYRK2 PCLO ARIH2 PCNX STAP1 PIK3C2A CYP4V2 ABCD2 TMEM246 MAP2K6 SLC32A1 CD180 EMP1 AGO4 ZC3HAV1 CCDC109B COX8C ARPC5 NBPF6 CKS1B SV2B HECW2 UBR5 ZNF585B LSMEM1 PRPF4B NBPF1 INS-IGF2 BNIP3 NBPF20 LEF1 PEAK1 STK38L MAPK14 AOC3 MTHFD2 MYPN CBLB NME6 RORB STAC2 SLC9A7 RRP36 ADAMTS19 POC1B-GALNT4 GABRB1 KLLN TUFT1 SLC25A13 LILRA2 MFSD2A PATE2 TLL2 LUC7L VANGL2 PRKAB2 QPCTL ALG13 ANO1 ZIC1 NAA20 ARL10 EGR2 ZNF281 PLEKHA3 ST8SIA1 GAS2L3 SMIM12 UNC79 CNRIP1 EDN3 ANK2 LMBRD2 SYT15 RAB23 AKR7A2 SENP6 COPS2 RIF1 GGPS1 CFHR4 GATAD1 DBT SRBD1 CGGBP1 SLC1A2 SDC4 INVS TMC8 SUGT1 PDGFB KLK2 HIGD1A TMEM57 SNRPD1 UQCRB LGALS3BP NAA50 ASPA TMEM209 HOXA10 SAMD9L ANGEL1 IFLTD1 RBBP4 ARHGEF3 PRKAR1A SUPT3H HLA-A HSD3B1 KIAA1244 SARM1 JHDM1D GABRB2 PEX13 PHF19 TTC5 GRIN2B CMC1 FEM1A COX20 HSPA6 C1orf122 CHUK RAB6B HS6ST2 GM2A PDPK1 C3orf17 SRGAP2 UBXN10 CAPG STK32A TPCN2 ZFP30 GID4 MPZL1 SPATA6 CRISPLD1 MYLK4 XRRA1 SPIN4 ZSCAN29 CYP7B1 KCNC3 AC015987.2 HARBI1 ITPR1 NSL1 EIF5A2 KIF5A SLC44A1 NT5DC3 PCK1 NPR3 AP5M1 DMRTA1 STX7 CNNM4 MYO5A IRAK3 CPM NDUFB5 P2RY2 GNG4 PLD5 N6AMT1 GPRC5A AL353791.1 LRRC34 SZRD1 CDC42 ACVR1C GALNT12 DIEXF CRIM1 SALL2 ZNF546 RP11-156E8.1 TNKS SMPDL3A SLC35G1 SNX2 C8orf22 NRG2 PYCR1 TULP4 ANKRD54 TSNAX RP4-758J18.2 MEI4 LTN1 KCNK1 SLCO1A2 BLOC1S3 RUNDC3B AC079602.1 CDH6 NDFIP2 IPMK CDCA7 RNF168 C12orf68 GABRA2 RNF115 STRAP DPY19L2 CLMN LRRC3C IL1RAP MTMR10 CDHR3 MED6 CSE1L MRPL50 WFDC13 RCAN3 CBWD5 POM121L2 ATG13 C4orf33 DOK6 KY SIT1 SMAD4 CD3D 7-Mar CSMD1 CENPC SLC4A7 STAT6 DRGX DNAJC18 MANEAL SLC24A2 ASB16 ARMC10 PSG1 PTCH1 UBD CDKL2 TADA2A COL25A1 CAMK2N1 GREM1 NFIC ELP3 AK2 VPS13A UTP23 PRPF38B OSBPL6 NSUN3 CELF3 AC005609.1 CCNT2 BCL2L13 C16orf52 GOSR1 JAZF1 DCDC2 ZNF621 JOSD1 ZBTB39 SHC3 C7 DYNC1I2 SULT4A1 DRG1 INHBB LONP2 MFSD1 ADRM1 TXLNA RAB31 DCAF16 BUB1 RNF138 TNFRSF13C MAP3K1 MRPS30 MR1 1-Mar TXNDC16 DIS3 AP3B2 THAP3 SPATS2 TMEM184B DIS3L2 ZNF292 CLEC6A FREM2 PTF1A SPIN1 IFRD1 TBX15 C20orf202 ZNF813 CTCF SNTB2 RGS9BP FUT10 AMIGO1 SKA1 SLC9B2 SHMT1 C4orf40 TMEM41B MOGAT2 TRIP13 ADA NEB KLK15 ZBTB8A COL13A1 HEBP2 RAP2B GABPA OPALIN TLR3 GALNT4 CTGF KIF3B PRICKLE2 CSRNP3 CDC7 ATF7IP CCDC8 TRIM58 GPR26 TAS2R14 GMEB2 EEF1A1 KDM3B FOXK1 POLR2D MRPL44 KANSL1L TMEM154 B3GALTL EPOR MTX3 RPF2 HHAT FAM179A C1orf109 ZNF558 FXR1 UBE2D1 FAM169B MX2 CREBL2 IDH3A ICK FAM120C B3GAT2 FAM200B SEL1L NDUFC2 ZNF831 UBE2G2 PHLPP1 FAM60A COPS7B SUCLG1 SH2D4A SLC7A2 TMEM67 NCKAP1 C1orf95 KDM4E ITGA4 CLOCK MLX KATNBL1 AE000662.92 PRKCE LGALSL STX3 SKA3 NCAPH SH3RF2 PDPN CNTN3 KIAA1737 TIGD7 FOXP4 GAPVD1 LETM2 ZBTB37 GOLGA8M KIAA1324L TMBIM4 GTPBP8 CACNA1D PRKAA2 IVNS1ABP PGP DUSP19 SULT1E1 ARMC3 NDUFV3 VEZF1 DNAJC14 PRKAR2B PPP1R2 TBC1D4 FOSL2 MRPL18 EPPK1 ZNF394 PHIP ASXL3 ZNF701 P4HA2 FAM103A1 MFAP4 RFWD3 PCDHB7 SEMA5B SS18 ECM2 ARHGEF37 IKZF2 RP11-676J12.7 PML DLG2 HIST1H3H GADD45G PPP1R12B TTLL12 LRP6 SERPINH1 UBE2S PPARA TPRG1 LEFTY2 CHP2 IL2RA STXBP5L ERP29 KCNJ1 GLRX3 TTR NFASC UVRAG NUS1 EIF1B LSM6 SMURF1 LRRC55 RANBP3L PRIM1 AL953854.2 ATP5L ONECUT1 CYP1A2 TPM4 COPS5 TG OPTN CABP4 SERINC5 FAM105A CCNL1 USP6 MRPL3 MAPKAPK5 INPP4B ELK1 PCDH11Y BMPR2 INHBA KLF7 RBP4 TFB2M NME2 LINC00923 GDAP2 GCC1 METAP1D MYO1A CTD-2117L12.1 FRMD8 FAM198B MGAT2 GPR183 USP31 UBE2R2 ZNF229 LRRK1 DENR GRIK1 TXK B3GNT4 SENP2 KLF8 ORC2 SOX5 PCGF5 KIF6 SFT2D3 PRKG1 WIPF2 DGKI ZNF283 ZNF354B RASGRP1 ZNF699 PSG2 SLX4 PCF11 UBL3 CAPRIN1 PIK3CG CD5L SLC15A1 COL4A4 GUCY1A2 POLR2K UTS2 TBCA TBC1D30 FMNL3 LRCH2 TRIM13 GPR115 AADACL3 MB ENPP5 LPHN1 STXBP6 SP1 ZC3H12D LSM5 GOLGA8H GINS4 SLITRK5 TAF1 SPNS2 S1PR1 CD79A CLCN5 SGCA C22orf29 EXPH5 YIPF1 TNPO3 GALK2 COLEC10 ANKRD13C JMY C16orf91 RAB3B DCUN1D1 REEP5 HTR5A-AS1 RP11-180C1.1 RTTN ARHGAP19-SLIT1 FGF9 ZNF844 PLCL1 ABI1 FGA SERPINB1 GSX1 IFI44L SLC1A4 VGLL2 MCM9 C11orf87 ZNF766 ENY2 CALU KIAA1671 TBC1D1 FOS MRPS5 EDA FIGNL1 ZNF226 SAP18 PAFAH2 PUS7 NPEPL1 WNT7A ZNF300 RNF144B MTMR7 C2orf71 TFAP2C ABCB10 ENDOD1 CNTNAP3 PPP4R1L CCDC105 GLUL ZDHHC5 HK2 ZNF460 RP11-383H13.1 ST8SIA3 COPS8 NGLY1 KLHL12 SLC30A10 ARAP2 GPR50 NR5A2 OXGR1 SLC27A4 NECAB1 TPK1 AVPR1A ASB6 FEN1 COL14A1 FAR1 CPNE3 GJB2 TNFAIP8 MICALCL ANKRD50 CALCRL IPO7 CXCL9 RECK NMNAT3 HECTD3 CEBPG GTF3C4 TNS4 CLEC1A SCARB2 PIGN KIAA1549 BAG2 PAQR5 PRR18 CCL28 ATPAF1 ISM2 PLAGL2 MICAL3 CBWD3 TMEM164 TRIM38 GCFC2 KIAA1456 CD300LB TMCC1 LRRC40 TM2D2 CRYZ WNT2B ETS1 SMU1 CASZ1 PSMB5 PRPF18 MOB1A GTDC1 SORD MFSD4 STEAP2 FAH MON2 CD81 HNF4A WDHD1 CNTNAP2 TIMM8B FRK RNASE11 TRIM44 ST6GAL2 PPAP2B CDK5R1 TRAF1 NBPF11 ACAD10 TRIM32 SCAMP1 PTS PTCHD4 SEMA3D BIRC8 SLFN13 CYP51A1 TGFA MMP17 ZNF34 IBTK TMEM170B C21orf91 PRLR RP11-17M16.1 FAM26E CDC73 SNX3 FGFR1OP FCRL4 CAMK1D GOLGA8O GOLGA8F TBX20 INMT ADSL FTSJ1 DR1 C18orf42 ANAPC5 SLC47A2 C6orf195 TMPPE MSH2 LNX2 CYLD GDI2 RAD51L3-RFFL NKX2-4 MGAT4A SELT SLC39A2 EIF4E3 SLC46A1 CHRM2 SLC12A5 KCNH5 PHF14 ERCC6 GABRA4 NAA30 RPL13 DGCR6 POLR3H NOTCH4 SKIDA1 GCNT4 MEAF6 MAP9 MRS2 VSTM5 RNF214 AQP1 MBLAC2 KLHDC10 FAM133A DPP8 TKTL2 ZFP64 CYP26B1 OTUD7B IL7R FAM83F TLR4 NKAPL RPL23 XG FAM84A ANKRD12 SDHC DDX51 ATF6 YLPM1 GNB5 RBM18 ZNF626 RGAG1 ADAMTS13 IL16 RPTN ITIH1 IMPG1 ACVR2B ZNF185 UBTD2 C11orf1 UBQLN1 RAVER2 SPATA5 ZNF470 NKAIN2 AKAP5 CD96 ACVR1B VCAM1 ROM1 UBA5 PTAFR CD1E FAM83D TMEM242 CSNK1G3 NOL9 SMIM20 SRFBP1 TSPAN6 C15ORF37 CREBRF SRSF9 FAM124B TDRP TMOD1 RABL3 RSAD2 TM7SF3 ANGEL2 ARHGEF6 KRTDAP TGFBR2 ORAI2 PLEKHA8 PLLP MST4 DTHD1 SRGAP3 NT5C2 PDK1 PHF13 AKAP13 WWTR1 DDX60L COBL PPP1R16B GAS7 TSSK1B ZNF337 HHIP GXYLT1 OXSR1 RAD51 TNFAIP2 AMOTL1 SLC35A3 ADH1B PEMT DLD SLC38A1 RGP1 PAICS PPFIA1 MYO1F GOLGA8I DNAJB12 CD300LF BRPF3 TCF7L2 C6orf106 ETV5 SLC39A11 GPD2 SETD7 TRAPPC4 CDK19 BTBD7 ITGA9 MYCBP VANGL1 NRBP2 TOR1AIP2 ULK2 FABP4 SLC35E3 LYRM4 CUL4A ADIPOR2 ENPP6 RIMS2 CYB561D1 SLC34A2 TMEM55A MCM6 ANKIB1 RNF125 OR4C11 SEC14L4 FRZB ART3 PABPC1 CLEC4G RRM2B CATSPER2 NDUFS5 CCR5 AL136115.1 GPR125 SLC5A1 CISD1 POFUT1 LYSMD1 PDLIM3 SNRPD3 CXXC4 HSD17B12 FAM107A FRG1B FBXO48 SUDS3 SERINC1 CAMKK2 SPCS2 SELRC1 PAX3 FBXO45 ERCC6L CNP CCDC121 LMX1B KSR2 RAD21 RRAGD HLA-DQB2 RPUSD4 C1orf204 RNF130 DLGAP2 SMIM15 DYX1C1 POMT2 KIAA0430 SLFN5 SOX11 TIMP2 ST13 SIX1 MKI67IP ITGB3 TCEA3 GPR156 QRSL1 TECRL PPIE SEMA5A STC2 TLL1 TMEM132B ZNF222 FAM9C COX4I1 AMT BLOC1S6 PTPLAD2 SAMD9 HSD11B1 S100A7A PVRL3 TXN DDR2 WWP2 ZNF326 LRRC16A COL5A1 ASB7 TRMT10C LGALS8 ANKRD1 IFT57 FKBP9 ZNF592 PTGS1 ALDOB ZNF579 PMEPA1 HOXD9 RPF1 STX16 DLAT CDC5L EFCAB1 TMEM119 NDUFA10 RBFA FOXO3 DCTN5 CEP44 LARS2 TRIM59 TTPA ZNF12 CLNS1A PSEN1 NUP43 SIGLEC7 ZXDB HIP1 ING1 C18orf54 DLX1 FAM120B CCDC67 TMED1 MFHAS1 RELN RASGRF2 MALSU1 LRCH3 VPS13C TRPS1 ATP5E HS3ST4 ATCAY MFSD9 LIG3 ERN1 GPR111 SERPINI2 DHODH B3GALT1 C4orf26 ONECUT2 C1orf116 FAM110B KRBOX4 TNFSF14 STRN CRISPLD2 CA6 C15orf37 MTMR3 RBM43 AR ZNF845 TRIM25 VSIG10 CDKN3 TMEM168 ITPRIP GLI4 SLC22A10 DHX36 E2F3 PHTF2 APOL3 CHCHD4 APPL1 FTSJ2 ADCY9 IL2RB FAM155B AC074212.3 LRPAP1 NT5C1A FAM160A1 SUCLA2 ST8SIA6 VAV3 MTMR11 CNPY1 GOLT1B ARHGAP22 PRKCA CCDC115 TTC38 C17orf51 SMARCE1 SUSD1 URM1 KRTAP19-3 STX4 TMEM106B LRIG2 MBOAT2 PISD CDKL1 1-Mar USP1 SAR1B SCN9A ZNF383 VHL CSF2RB LMBR1 PARM1 C6 SMAD5 SLC16A10 ANKRD23 BCKDHB NBPF15 IFNA8 BET1 FRMD4B ATRN PSMB9 FBLN5 SLC25A53 TTC30A HAPLN1 CRISP1 ADRBK2 C3orf67 PDE12 NADK2 SLC35B4 SMCR8 RPS6KB1 GPLD1 RP11-332O19.5 LIPT2 ZNHIT6 SPOCK1 DCAF8 SPEF2 CDCA8 APBA1 ZNF638

**Supplementary material Text 2:**

The list of prediction of hsa-miR-199a-3p target genes of venn diagrams.

**Tarbase TargetScan miRBD: 60**

CD44 ATRX NUFIP2 ARHGEF12 CMIP NEDD4 EMC1 LCOR ATAD1 DUSP5 PIK3CB MAPRE1 CDK17 IFFO2 QKI RUNX1 FN1 SNN C6orf62 MTOR CD151 ADRB1 VLDLR TAB2 SUMO3 SCD MCFD2 RABGAP1 ANKRD17 NET1 SLC7A11 GREM1 FXR1 PDE7B NAP1L1 DDIT4 SERPINE2 G3BP2 PLAG1 EPG5 RBM47 SLC20A2 APLP2 DCP2 SMOC1 VAMP3 CDK5R1 KCMF1 GOLIM4 TMEM87A NACC1 RAP2A PPP4R2 SLC7A1 ZHX1 CALD1 ETNK1 GNA12 TAOK1 EDEM3

**Tarbase miRBD: 24**

DONSON VPS33A IRF2BP2 PRKCB EIF3M HVCN1 ZNF614 ATP6V1A MAP3K1 HSDL1 RAP2B PRPF40A MTF2 MYSM1 SLC22A15 TBX3 NABP1 UBQLN1 IMP3 UPF2 B3GALNT2 NCOA4 TRIM5 CYP1B1

**TargetScan miRBD : 208**

ERBB4 TACC2 TNIK FGL2 ITPK1 ADD3 HIC2 DEPDC1B TUBGCP3 C2orf49 COL4A5 SLC33A1 ZEB1 FOXQ1 TSPAN3 MFSD6 TEAD1 MDGA2 PHLPP2 ITGA3 LPAR4 ABL2 PTPRZ1 HNMT MECP2 MAP3K5 ENOX2 ABHD4 MPP7 DLX2 CHMP5 DCBLD2 SCUBE3 RAPH1 SEC16B EXOC6B ST8SIA4 SEMA3A LLGL2 RFX3 CBLL1 MEIS2 ACOX1 SH3GLB1 SDC2 INO80D KDM5A NACC2 KDM6A ZBTB18 HACE1 AEBP2 TANC1 ITGA8 CXCL11 ZBTB20 MYO10 FUBP1 UBE2W MAGT1 ZBTB43 ACVR2A RPS6KA6 GPBP1L1 CBLB PXN LIN28B PLEKHA3 PAQR3 PDE8A PSD2 PAWR ELAVL2 ARHGEF3 CYB5R4 PEX13 CD2AP BEND7 IL13RA1 KIAA0319L ADAM10 UBE2J1 TGIF2 ZNF217 DNMT3A CNOT7 ACVR1C ITGA6 EBF1 ZNF740 GCNT2 KLF12 TMED5 FAM199X CISD2 SIK2 PNRC1 ITGB8 BCAR3 SLC24A2 RUNDC1 SLITRK6 LZTS3 ZBTB4 CHAD TMEM62 MVB12B BBX PIGB CNEP1R1 WDR47 KATNBL1 PRKCE ARHGAP20 NUTF2 DPF3 SESN3 PLCB1 CNIH2 PHF6 MLLT6 AK4 NLK FGF7 IPPK SP1 MAP3K2 FUT9 CFL2 SECISBP2L EML4 PHYHIPL GNPTAB CEP85L PPP2R2A CPEB4 PLEKHH1 ANKRD44 ALX4 DIO2 UXS1 NCOA1 CDK7 NID2 RABEP1 ST6GAL2 TRIM71 RB1 CELF2 MAP3K4 CCDC85C KMT2A FBXW11 PDE4B FAM133A LRRC1 ESRP1 ACVR2B FLRT3 ADAMTS3 MED12L GGNBP2 BRWD3 CREBRF LOX SYPL1 SLC39A10 SLC38A1 PALD1 CA5B PAK4 CSRP2 CELSR2 PTPN3 PDGFRA FOXP1 NOVA1 C9orf40 KCTD16 CHSY3 PON2 YAP1 ITPKC WHAMM ARL15 AREL1 CABLES1 TPPP STC2 PTPRU NAA25 MARS2 KLF13 PPP2R5E EPAS1 CTNNA2 SBNO1 WNK1 TMSB4X KPNA6 KDM3A ADAMTSL3 KLHL3 DTNA KTN1 COL12A1 MEDAG RNGTT

**Tarbase TargetScan: 48**

NFIA LIN54 TRA2B YWHAE CELF1 ARHGEF2 STRIP2 IREB2 USP9X YWHAZ ANKRD52 TMEM170A RPAP3 DSTYK DDI2 ZNF710 ZKSCAN1 HIPK3 CSNK1A1 TSPAN14 USP37 PTAR1 ATF7IP GREB1L FAM60A ADARB1 RORA TFAM LONRF3 TACC1 PTPRF MBNL1 PURA FOS CAD MAPK8 HOXA9 NOL7 TAB3 VANGL1 CAV2 PVRL3 ZNF703 TMEM184C RFX7 GOLGA4 FAM49B UCK2

**miRBD: 184**

GFOD2 AMZ2 CCDC28B TRMT61B PTPRE SYNPO2 MBL2 ARL6IP1 SORL1 CXADR FAM129A ARHGAP21 BRCA1 CLCC1 ROCK2 NECTIN2 TBC1D12 ANO5 CCDC141 ZNF749 SLC16A12 CETN3 LAMP3 SEMA3E PCDH7 FAM76B CCSER1 LRRC17 FAM110C SOS2 DCLK1 TAF2 RGS4 GRK3 KCNH2 ANKRD61 GIP ARL6IP6 ZNF227 IMMT PNOC IL1RL1 DNHD1 COLCA1 GORAB CNR1 WAPL SMIM8 PROSER1 DNAJC30 ASAP2 FMN1 TENM1 DELE1 KLLN RP1 RIMBP2 UQCRB XKR9 PHKA1 DOLPP1 HYPK FDX1 TCTA GFM1 IRAK3 P2RY12 FCGR3A PYHIN1 PTER OSTM1 DIRAS2 TNFRSF19 PLCXD3 SLC44A5 GALNT7 HS3ST5 DNAJC18 TWNK BCL2L13 LONP2 CHKA YES1 DIMT1 MAN1A2 MCCC2 CR1 TRIP11 SLC22A5 ATL1 PTPRC THAP2 FAM20B GIMAP2 KDR SLC5A7 NTRK2 KIDINS220 RNFT1 OPN5 ID4 CAPRIN1 SNX18 NETO1 TENT5D NDST3 PLGLB2 RAB6A SLC49A4 RAB3D NECTIN3 TCAF2 LRRTM2 WDR7 LPAR5 SPIRE1 GABRP PDE5A SINHCAF ERO1A FCGR3B CCL28 DLX5 SRD5A3 LRP2 LOC100144595 ZCCHC17 PCMTD1 RSBN1 RAB6D KHDC4 RALGPS2 CAMK4 ID2 44453 TMEM218 LUC7L3 HAT1 SEC62 RAB6C MS4A7 CCDC88C ARG2 TMEM220 PCDHB12 CABYR ATP6V1C2 C9orf170 PLGLB1 NLRP1 WDR41 UPRT SRA1 SYNJ1 KCTD7 HBS1L NRBP2 CDC42BPB CLDN8 STARD9 CDNF KSR2 WFDC8 ZNF736 UBR2 PIP5K1B RNF19A MPRIP CLEC12B N4BP2L1 KPNB1 MKRN1 APLF ZBTB25 DPAGT1 LIG3 EOGT NXPH1 UNC45A EPB41L5 SYT16 HGF GPAT3 THAP9

**Tarbase: 556**

PCNXL3 HMCES DLG1 UGCG BTG2 HIVEP1 EIF4EBP2 DHCR24 WASF1 MEF2D PANX1 SLC10A7 RGMB G3BP1 UBE2G1 PELI2 AGFG1 RDH10 SETD2 CA13 CHORDC1 G2E3 AP1G1 ZBTB34 MET CERK PHYHIP ARL1 OSBPL8 MSN CHID1 PIKFYVE C4orf3 SMARCD1 POLR3K TSC22D1 FASN DIAPH2 TBK1 FUBP3 STAG2 FOXK2 AP1S2 PRDM2 LNPEP CEP350 CLN5 PRKAR2A YTHDF3 CHRAC1 GGA3 SSU72 TMEM159 USP14 DNAJC5 GLRX2 DNAJA4 HECTD2 SGPL1 FAM89B RBM23 C3 FAS FOXA2 HSPD1 EIF1 N4BP2L2 LEPRE1 BTRC UBR3 PGAP2 OTUD3 ZNF8 MGAT5 CPEB3 SLC2A3 ATP13A3 MAFK KIF5B THADA CSDE1 CLPTM1 ATP5B SPRTN HMGN3 NPM1 CARD8 URI1 MEF2A RAD9A HMGN4 ZNF24 RAI14 LTBP1 NDUFA2 MBD5 ERO1L MAU2 UBN2 COX10 MOB4 PAPPA PAN2 EVI5 DHX15 AGPS DLL1 BCL9L DPF2 BRD8 RET LZTFL1 MASTL MID1 AURKA PALM2-AKAP2 HCFC1 GRSF1 ZNF566 AHR ZFP36L2 PDRG1 PPP1R12A UHMK1 FAM69B NFE2L2 MTF1 ZNF778 NEK6 PGM2L1 NEK7 PAQR4 ZNF805 ARHGEF16 JADE1 EMP1 SLBP GOT2 RRAS2 AASDHPPT FAM222B ZDHHC4 ANKRD42 AHRR MANF DYNLL2 PRR14L MTHFD2 TPT1 CCDC113 CDK6 FBXO46 DCTN1 TNKS2 LUC7L DIXDC1 ASAP1 NSD1 AMMECR1 SEC13 FRMD6 CWF19L1 TLK1 RIMS3 ZNF384 RIF1 RER1 GATAD1 GOLGA8A CLDND1 TANK KIAA1147 CACUL1 TERF2 SLC38A2 CCDC86 ATN1 CASP14 KALRN PTGS2 KIF2C NT5DC3 EHD2 ADO OXR1 ATG4D ELF1 TCFL5 VEZT KDELR1 CDC42 TBX1 SETD5 TNKS DNAJC25 KDM6B SURF4 KCNK1 GLOD4 LIG4 AQR PEF1 FAM107B RIMKLB CRYBG3 CCDC127 TRIB1 NR1D2 PHF12 MDH2 SOCS4 CTSD VEGFA SLC4A7 TGFB1 ERCC3 TFRC MPDU1 ARPC1A SPATA2 TES SNRNP48 HIAT1 FZD10 ILF3 SLC35C2 SUPT16H PLK4 CCNT2 ZC3H12C FBN1 PLOD2 BGN PWWP2A ABT1 ARID2 SMG1 CALM2 ATL3 MAP4K5 LAMA5 KIF1B GNA13 PSRC1 HEG1 PANK3 MCL1 TXNIP FAF2 CCND3 CBX2 IGFBP5 TFG CTDP1 ERGIC3 CRTAP FBXO30 RPP30 ZMAT3 STMN1 MTMR9 RAN ETF1 NR2F2 INTS3 UBE2D1 AGPAT6 C11orf30 NUP210 AVL9 EFEMP1 TNRC6B NDUFC2 RNF213 PARP1 MYO1E ZFX URB2 TPD52 FH TAGLN TAX1BP1 VEZF1 SRCAP HAS3 BPGM C12orf49 GRAMD1B SLC11A2 FLT1 FEM1C SIAH2 ATP11B HNRNPD EXOC4 CBFB PPP1R12B KMT2D ZIC5 MARCKS TLE1 PLK2 EMC2 ENTPD6 BCL2L11 POMP ZFP91 MYO9A TFB2M GCC1 BRPF1 MAPK6 WDR33 TBCE RPS20 RAI1 MAPK1 DCAF4 CPEB2 PDLIM1 HNRNPA2B1 ZNF430 TNRC6A PTBP3 DIP2B MBD6 ETFDH TPP1 EFHD2 HNRNPF MAML1 CYB5B LIFR MUC6 TOX4 DENND1B ZBTB33 MGA TFAP4 CAPN2 EXPH5 ZNF451 REEP5 KIAA0391 PRC1 CREB1 DCLRE1A SRD5A1 MYT1L PSAT1 MYCBP2 DBI PIGQ MPHOSPH6 MARK2 SLAIN2 GNE PSMD6 DDX6 RPL28 ACTR1B TROVE2 FNDC3A MICB SZT2 HNRNPAB ARAP2 VPS18 MICAL2 FEN1 CPNE3 TMEM39B GOLGA2 THBS1 CAMSAP2 TMEM52B TSG101 HELLS GTF3C4 CMAS TMBIM6 STK4 C15orf39 HSP90AB1 ETS1 ZNF121 PMAIP1 GLYR1 SMU1 MTHFD1L WDHD1 SLC30A6 CYP51A1 IBTK STAU1 TMEM189-UBE2V1 TRIM28 STOML2 6-Mar DST FLAD1 ZBED4 KIAA1468 RAB11FIP1 PPRC1 H2AFV OAZ3 TOMM70A TRMT10A SLC30A5 SYDE1 PPP2R5D ABHD17B BACH1 ACVR1B PPP2R2D DSEL DTL RAPGEF2 OXNAD1 SRSF9 SMG7 RNF219 PLEKHA8 MLEC PURB SDE2 PDIA6 LURAP1L ZMYM2 C8orf76 DYRK1A PEMT RGP1 CDKN1A BRD2 MPHOSPH9 HLA-B TMEM30A ARHGEF17 ATXN1 ZNF431 GPD2 ARFGEF1 IMPA1 BTBD7 AUP1 TOR1AIP2 CPS1 PIGO DESI2 ADIPOR2 PIK3R1 FAM217B MCM6 PTPRJ NDUFA12 STAMBP ZC3H10 SOCS5 ASXL1 FBXO45 CUX1 NNT CDC42SE2 USO1 VMA21 BLZF1 ZNF654 CD164 PTP4A1 ARHGAP35 CLIP1 RGPD8 2-Sep RBM27 ADNP RGPD3 VIPAS39 ZNF714 BLOC1S6 ADSS SKIL BCL11A HMGCS1 F11R KIAA1462 POLR3F PYGO1 HDGF RNF114 KCTD12 ZNF207 SFT2D2 PSMA6 DCTD CNIH3 GTF3C1 UBTF CEPT1 RNF13 KHDRBS1 MYLK3 JDP2 NUP43 ELK3 ZXDB FAM111B CYB561A3 ANKMY2 SLC39A1 SEC24A MAP1S AKAP11 TRPS1 MAMDC2 CAPN7 LGR4 PGM3 MTMR3 UBE2D2 BMP2K DDX3X DGCR2 BMP5 ITPRIP PHTF2 TADA2B SRRM2 TMF1 GIGYF1 MYBBP1A SLC16A1 SF3B3 LAPTM4B MAD2L2 ARID1A RCN2 ERC1 PAK2 CENPF RPS6KA3 BRWD1 IGF2BP1 UQCRC2 SLC19A2 B3GNT2 FNBP1 KLHL24 MLLT4 RAB7A CNOT6L SENP5 GOLGA8B LBR PCIF1

**TargetScan: 152**

PLCE1 IGF1 NUP155 DAZAP1 ATXN7L1 TIA1 SMARCC2 RNF14 FRY ATAD2B DIRC2 SLC23A2 RP11-644F5.10 SP6 SAMD12 CECR2 GNAS GFPT2 PGRMC2 GAN MSH6 BTN3A2 PRDM16 ABCA1 C8orf44-SGK3 MMP16 MYO5B ATP1B4 PPP1R9A THRB F9 CDC42EP3 NRIP1 ITGA1 TMEM33 KDM4B ARMC8 MMAB TLE2 EGR1 AP3B1 C1orf21 SH3PXD2A NRF1 SRR AC073610.5 MOGS CDC42SE1 RORB COPS2 CERS1 RP1-170O19.20 STX11 C10orf53 SETD6 WNT9A REPS2 COL19A1 CRIM1 HHIPL1 SCAI TULP4 CCDC144A NR6A1 PHPT1 CREBZF CXXC5 UNKL FIGN RAPGEF4 DLL4 MMP24 B3GAT2 CC2D1B NDE1 TBC1D4 CITED2 LMAN2 RCOR1 YWHAG SOCS7 C9orf72 KLF7 TXLNG UBE2R2 RNF216 SOX5 PCGF5 PLXDC2 PPM1B STIM2 RREB1 TRIM13 ANKRD13C LEPROTL1 CALU ZHX2 BAZ2A FGF2 EHD3 MFN1 PURG PTPRG SLC30A1 UBE2H PDE7A KAL1 IKZF4 MON2 SUZ12 PPAP2B SLC45A4 ZBTB10 FAT3 TMEM200B PCDH17 FGF16 THRA ACER2 ATF6 ASB14 LMO3 DDHD1 NAV3 STK11 CHP1 OXSR1 FAM53B PDS5A SOWAHC C20orf194 ASTN1 CHRM3 ARID4B DDR2 POU2F1 SGK3 FBXO28 PPARGC1B NRD1 TWIST1 FAM180A TMEM110 TMEM106B WAPAL VGLL4 FRMD4B ADRBK2 NDUFB9 CCDC88A MEF2C IQGAP2

**Supplementary material Text 3:**

The list of prediction of hsa-miR-1306-3p target genes of venn diagrams.

**Tarbase TargetScan miRBD: 2**

GUF1 RNF169

**TargetScan miRBD : 21**

LPP GLYATL3 NFIX NXF2B DUSP4 DCUN1D4 NXF2 SLC12A6 GNA11 SLC43A2 UBE4B STON2 TIAM1 BFSP1 FOXN1 TANC2 SHOX RRAS ZMPSTE24 CDS2 FJX1

Tarbase TargetScan: 13

ATP9A HNRNPA2B1 NCOR2 LIMD1 H1FX ATF6 FOXP1 MIDN ZNF91 AIF1L INHBB AGO3 AVL9

**miRBD: 2**

JADE1 DNMT3A

**Tarbase: 104**

USP30 MTF2 LRRC41 VEZF1 MSL1 NSMCE4A ANKRD13B SEC16A CHD9 RMND5A FOXJ3 ZIC5 DUSP7 RPL7A PKD1 PRDX6 TPM4 ASCC3 LMNB2 SP6 RAC2 LRP1 DNMT1 ADAMTS1 KDM5C FAM63B CAPNS1 C5orf28 SPTLC2 TPI1 SOCS6 EMP2 HAND1 TSC22D2 GPN2 ANKFY1 YWHAQ CKAP2 CLPTM1 TMEM173 CAPRIN2 PPP1R3E HCFC2 TAF10 INO80D PMAIP1 WBP2 CCNG2 PPP1R9B GSPT1 NANOS3 LEMD2 HSPA4 RNF111 MED28 DR1 TFAP2A PBX1 USP9X RPL13 APOBEC3C HSPH1 PPP1CC RNF40 MARVELD1 C3orf58 KLC1 C1orf198 KBTBD2 CIT VIM RARS2 PPP2CB TIMM13 USP9Y CTPS2 NMT1 CUX1 MIF KANSL1 TMEM183A SEPHS2 XYLT2 CREBZF LIPT1 HIGD2A TRPS1 PEAR1 AKAP17A SMAP2

MT- ND1 VPS13D SH3GL1 PANK3 WDR82 NCOA6 MAZ IGFBP5 ATF7IP ETF1 CTTNBP2 CPSF2 RAB7A ZDHHC17

**TargetScan: 628**

MCF2L KIF26B TSPAN11 MTUS2 PDPN WDR81 CACNA1C AP5Z1 C6orf211 ZBTB37 ARHGAP31 C5orf45 SALL1 GLTP FAM178A ZSCAN18 NAGPA ZNF646 PLBD2 TGFB2 WNK2 GOLGA7B FRMD3 HMOX1 ARHGAP32 MEX3B STARD8 HIC2 ZNF701 ROGDI HOXD11 CACNA2D1 IMP4 PADI1 DFFA ZBED3 SMC2 SERGEF NDUFS1 TFDP2 RCOR1 SOD2 TNC CLHC1 FAM86A KCNAB3 PPP1R12B PHLDA3 C7orf55-LUC7L2 LPAR3 AGBL3 AFF2 YAE1D1 ZYG11B PNPLA3 AAR2 SOX4 RAB40B RRP7A C6orf223 SLC22A23 PHC1 PLEK CHMP2A ANKMY1 CSF3R GNAI2 LHFPL2 NMNAT1 NCS1 TANGO2 HDAC4 MRPL3 ZNF500 GDNF TECTB ELFN2 ZYG11A MECP2 PNPLA4 PRKCB CAND1 METAP1D ZNF623 NOS1 WDR33 ABCG1 LCLAT1 ZNF277 FAM65B CLCN6 TENM4 RNF126 CEP63 CD160 GSX2 DSCAML1 SBF1 RPS23 ORC2 GPR37L1 ZNF430 DGKI PLXDC2 ZNF793 RIN3 NUP93 C10orf128 SLC7A5 CYP20A1 FABP1 GLTSCR1 NFIB GNAS LTBP4 NPC1L1 EVI5L GOLGA6B UMODL1 MT-ND4L CTD-3214H19.16 STAU2 FFAR4 DRD5 HIF3A ZNF285 PRKD2 CHML ZNF321P XIRP1 ZNF317 DNAJA4 LBX2 HTT ADORA1 TMEM223 HTR5A-AS1 ALDH2 KIAA0391 TPGS2 ARHGAP19-SLIT1 EMC1 SWAP70 ABCB9 PPP1R3D SYT5 ZNF322 SLCO2A1 NEUROG3 ARHGEF15 PRDM16 C1orf216 PLA2G15 HIST1H3B CCDC101 SLC22A15 HSPA14 SLC25A16 SLC37A2 CHRNA4 LPIN2 NOP9 HIST2H4B FAM182B C2orf43 RBP2 OTUD3 YME1L1 AC132186.1 MBOAT1 WDR35 MGAT5 PLA2G4E KCNN1 KIF11 BBS4 TCEB1 PSD4 TBC1D10C OPCML ARID3A ACOX1 TLX2 DENND1A FGF2 C2orf71 SEC14L1 ZSCAN25 NAALADL1 LMF1 FAXC ENDOD1 ZNF544 TROVE2 MPP6 ZDHHC5 TMEM117 LIPG APOLD1 TRPV4 TNFSF8 CELA1 TMEM251 ZNF346 AGPAT3 TMEM221 C9orf66 NR5A2 LDB2 IGF1R TRAF3 CXCR2 LUC7L2 CPEB4 TNFAIP8 HLA-DQA2 TRIM54 ESRRA HECTD3 MRGPRF TNFRSF1B CAPN1 LHX6 KLHL18 BACE2 ZBED1 PIGR TMEM164 KDM2A IKZF3 QKI THBS2 CALN1 XPNPEP3 PCDHB16 MTDH METRN AHCYL2 SLC5A10 GLYR1 GATA5 ZNF641 GNG12 CNTN2 MOBP HOXB13 NBR1 C16orf11 PAPPA PPAP2B GDF11 CCL1 RAB26 ZNF655 CXorf21 UBXN7 ITGA8 KCMF1 RP11-463J10.2 FAM71B MCMDC2 ZDHHC13 KCNQ5 DLL1 GGCX ADAMTS2 GLYAT BRD8 CAMK4 SLC25A19 SUV420H1 TLE2 FGFR1OP ANKLE1 POGK MTO1 ADAR SMTNL2 GOLIM4 ZNF366 PTPN2 GRIK3 GPR56 CACNB2 METAP2 PARP14 KIAA0586 RAB21 PTGER1 PERP CEBPA REXO1L1 KIAA1468 DLG3 ZNF425 HEATR6 AL590235.1 ZCCHC24 GOLGA6C SEC14L2 SLC35E2 MTSS1L UBE2N ATP5J2-PTCD1 PRIM2 NEK7 TMEM246 KCNRG NACC1 ECE1 SNN FAM83F RP11-295D22.1 SOWAHB ZNF559 LEPR GABRB3 CEP41 DET1 BMP6 RPTN IL16 FAM230A MSTN FECH ACOT2 PRH2 IMPG1 MOGS LRRC20 CATSPER4 ZFP41 CKAP2L ZMYND12 AHRR SLC13A5 AC007405.2 FBXL16 DYNLL2 ZNF724P CAP2 SHROOM4 ZNF594 VWA2 UBA5 LRRC3 C17orf100 SMTNL1 CLCN2 PTAFR TOR1AIP1 SHPK PCDHB3 GNA14 VAPB KB-1980E6.3 VBP1 MOCS3 MRRF CAMK2B C21orf49 ZNF417 AFF4 ERVMER34-1 SOX12 ARL10 MARK4 GAS7 EDAR SLA ZNF93 MBD3 ZMYM2 TAB3 C6orf132 EID2 C9orf69 CENPP RBBP5 MYO1F FAM177A1 CLEC2D WSB2 POLR3G FAM227A ZNF726 SETD7 CYB561 MAD2L1BP NUDCD3 NREP ELAVL1 AP1M1 NRCAM KIAA1244 ACAP3 ZMIZ1 AC024940.1 TBRG1 FAXDC2 AKT1 GATSL2 HSPA6 SRGAP2 UBXN10 SGSM2 PQLC2 STAMBPL1 ADCY2 XAF1 ZNF737 ABCG8 PTCD1 TPH1 HEMK1 GRAP KXD1 PAPLN HS6ST3 PRKX IFNAR1 CSF2RA TCP11L2 CDC42SE2 KMO CLDN1 RPAP2 SLFN5 TGFBR1 SIX1 ARHGAP35 GPR156 AGPAT4 BTBD9 CARF LRPPRC TOMM40 RNF151 HIST1H2BD GALM FLNB WDFY4 TMEM132B CD93 LRRC3DN TULP4 MEI4 KLHL11 TUB SYT7 MKX KLF12 KCNN3 FKBP5 NFAT5 CDK4 VSIG4 DCAF7 CACNA2D4 MCAM CCAR2 SBK2 GCH1 KLF13 DNAL1 C10orf113 CRTC1 PLCXD3 TRMT10C NAP1L6 GPRIN2 APTX DCTD CLEC12B RFESD CALCOCO2 FICD HTR2A ZNF703 MAT2B KCNJ5 ZKSCAN1 TRIB1 LRP2BP KCNMB3 DHX37 PLA2G12A POFUT2 RALGDS PRKACB PEG10 LPIN1 KCNE1L MAP2K4 PCBD2 B3GALNT2 JDP2 TMEM184C CDKN2B PRR26 CYB561A3 BCAT1 PSAPL1 MFAP3L ARHGAP19 PHOSPHO1 GNA12 ZBTB7C PDK3 GOLGA6A AMACR SMIM19 IL18R1 VPS53 COX19 ATCAY TM6SF2 UBE2L3 MFI2 KCNJ15 AC005609.1 C10orf85 6-Sep ART4 EVX1 KIAA1841 OIP5 SLC35F3 CRISPLD2 ZNF557 FSD1L MYO1D ARHGAP10 LONP2 ARFIP2 AL590822.2 TPST2 WNT7B C21orf2 AP005482.1 ZNF486 TNFRSF13C ZMYM6 MAGI1 SLC35E2B CASK TIGD5 NCAPG IL13 KLHL3 FKBP6 RCC1 HEYL USP44 SPATS2 SLCO4C1 MIEF1 HUNK EGR3 LRPAP1 AMOTL2 RPL36A-HNRNPH2 NT5C1A TSPAN14 METTL21C FOXO1 KAT7 ARHGAP22 FBXL19 FLJ20306 BEND4 SHMT1 BAIAP2 USH1C AGAP2 LAMC1 STAT1 HEBP2 MBOAT2 NFAM1 ZNF629 MBD1 RNF44 WAPAL RCN2 NPTXR ERC1 TLDC1 SEC22A PAK2 LPHN3 UBE2T RPS6KA3 FAM131A AC013269.5 FOXK1 POLR2D MRPL44 APC2 SMURF2 NCR3LG1 ZNF558 C8orf82 USP3 PPP1R27 C20orf112 CD3EAP GPBP1 RNF165 NLRC3 UBC C4orf50 AGPAT6 ATRN CD1C NSUN4 ISY1 C19orf59 ALS2 TBC1D16 MOCS1 PLXNA4 PTDSS1 OLFM2 GRAMD1C DARS2 CXCR5 BAIAP2L1

**Supplementary material Text 4:**

The list of prediction of hsa-miR-30b-5p target genes of venn diagrams.

Tarbase Targetscan miRBD：539

SAMD4A GSKIP ITPK1 FAM214A GALNT3 UBAC1 HIC2 CHD9 HOXA1 SCYL3 NFIA CHD1 LHFPL2 ELOVL5 ARL4A HERC2 HOOK3 PCGF3 CAND1 SNX16 EED ATL2 CECR2 SEL1L3 CEP350 PPP3CA GFPT2 RAD23B CCNY LRRC8D FBXL14 GATA6 SLC6A15 LCOR CCDC43 OTUD4 PLXNA1 RFX3 RBFOX1 CPEB3 SON MZT1 PPP1R9A OTUD6B AVEN ADAM19 ADRA2A SEC23IP ZBTB11 PIP4K2B TRIP12 RGL1 DLGAP4 KIAA0355 INO80D SUV39H2 QKI NCAM1 ZBTB18 UBN2 CNOT6 PICALM CARS FAM126B ZNF711 CHMP2B RTCB HNRNPUL2 PGM2L1 SCN1A STRIP1 SNAI1 CCDC6 SPCS3 PDGFRB CELSR3 LIN28B NSD1 NEDD4L NHLH2 PAWR AGO2 DSTYK ELAVL2 FAM160B1 PGM1 KLHL28 CYB561 FBXL20 ZNF827 ZNF264 RAB15 BDP1 UBE2D3 GALNT2 ANKRD17 PLEKHO2 DPY19L3 NFAT5 GIGYF2 TMOD2 FAM199X IRS2 BTBD10 NFATC3 PTPN4 BCL6 ELL PITPNM2 SLC7A11 ZBTB41 GRIN2A SLC5A3 WDR26 BCL11B SOCS3 UNKL KLF9 FIGN TSPAN2 E2F7 YES1 FAM91A1 SP4 RAB4B GNA13 AMOTL2 CNKSR2 WDR82 CDH20 CBX2 MAN1A2 SSBP2 REEP1 DLL4 SETD3 STK39 PRDM1 FAM13A SMAP1 AVL9 TNRC6B FOXG1 GLCCI1 ZDHHC17 MCF2L COL9A3 DCTN4 FAM126A PPIL3 NUCKS1 FBXO34 RCOR1 CCDC117 PPP3CB USP15 SRSF7 LSM14B UBN1 VKORC1L1 SFXN1 YAF2 LCLAT1 G3BP2 CHD7 WDFY3 HNRNPA2B1 TNRC6A PPP3R1 MYSM1 SNX18 PPFIA2 MAML1 PIP4K2A SEC22C CFL2 RASA2 BAZ2B CPSF6 ZDHHC20 TMEM181 MINPP1 EML4 CEP85L TUSC3 TBL1XR1 PHACTR2 RPRD1A MAPK8 CPEB4 HCFC2 VIP CCNE2 PDE7A MTDH PAAF1 LMBR1L MBTPS2 EXTL2 FUCA1 SOCS1 RAB32 CAMK4 ERLIN1 MTA1 SBK1 SLC25A36 INTS2 ZNF507 CSNK1G1 TMEM87A P4HA1 AZIN1 ZDHHC21 ZFAND5 RAPGEF2 MAFG ZCCHC2 FAM124A CHIC1 ACAP2 SYPL1 PTGFRN MAP3K7 PPIP5K2 ZNF746 ATXN1 RASA1 S100PBP BNIP3L GABRA5 CCDC71L NRXN3 FAM210B KLHL20 NOVA1 RAP2C FZD3 SNX8 CFDP1 DLG5 FNDC3B RNMT JAKMIP3 SIRT1 OVOL1 INPP4A IGF2R LARP4 SKIL RAB10 EFR3A CERS6 IFNAR2 R3HDM1 PLA2G12A NRBF2 LIN7C LONRF1 JDP2 ERRFI1 GALNT1 SEC24A B4GALT5 SGK3 PPARGC1B TMEM56 OMG EEA1 GIGYF1 EDEM3 NDEL1 KMT2C ARID1A SATB1 BRWD1 AGO3 STT3B PSD3 AFF3 MLXIP KHNYN RNF122 ANKRA2 IER2 NUFIP2 MBNL2 MEX3B NID1 TIA1 TRIM23 DPYSL2 SOX4 MFSD6 FLVCR1 EPDR1 GJA1 GNAI2 ASCC3 MECP2 MAP3K5 STAG2 ZNF704 LRRC8C SYNGR3 YOD1 NAPG DGKH CDK12 RTN4R NEDD4 YTHDF3 ZNF148 FYCO1 REEP3 GNPDA1 SEMA3A DDAH1 EDC3 MIER3 CADM2 TWF1 SLC12A6 PAX9 BCL9 GPCPD1 MAGI3 CAMK2D RAI14 SGCB N4BP2 IFFO2 NACC2 ARID5B KRAS ACTR1A DCUN1D3 EVI5 MYBL2 PLXNC1 SNX10 STOX2 ZFP36L2 PBRM1 SSR3 GOLGA1 NR3C1 PROSER1 PFN2 CDC37L1 NRBP1 AP2A1 GABRB1 CHL1 AFF4 AKAP10 TTLL7 FRMD6 DGKZ RAB23 SAMD8 VIM CADPS B4GALT6 RHEBL1 KIAA1147 SLC38A2 CD2AP GID4 ADAM9 ZSCAN29 NT5DC3 MYO5A SPEN REV1 ADO OXR1 SETD5 TULP4 ZBTB7A NR6A1 NEUROD1 JPH4 GALNT7 KIAA2026 PER2 TMED2 PRUNE2 SLC4A7 CAMK2N1 BCOR PELI1 CELF3 CCNT2 JOSD1 ZBTB39 CSNK1A1 MAP3K1 RAPGEF4 ZBTB6 SNTB2 ZNF521 RAP2B USP37 BRD1 GMEB2 ZCCHC14 KCTD20 PRPF40A SEC23A AGO1 IRF4 CPNE8 PTPRK PPP1R2 GRB10 FAM13C TBL1X PHIP RBM12 RNF220 IKZF2 CBFB SACS LRP6 RORA CAPZA1 FAP DDIT4 NUS1 TPM4 UBE2R2 CPEB2 ORC2 PCGF5 SMARCD2 NFIB PIGA LRCH2 LIFR SRSF10 MAGI2 TNPO3 MAP3K2 DCUN1D1 MBNL1 FOXA1 ZFC3H1 TBPL1 JUNB SCN2A TP53INP1 XPO1 RHOB GNAQ EPG5 FNDC3A ATP8A1 CSAD NAV1 PLAGL2 MSI2 TMCC1 PAFAH1B2 SH2B3 RASD1 SLC41A2 TMEM170B C21orf91 FAM43A ZNF280B TNRC6C KMT2A ZEB2 STK35 USP48 ARID4A FAM133A MKRN3 SERPINE1 RARG BRWD3 TBC1D10B GXYLT1 AMOTL1 RAB11A CUL2 SETD7 HNRNPA3 HIPK2 DESI2 FRZB SLC6A9 TMEFF1 USP22 KCTD16 SLC9A8 FBXO45 PON2 TIMP2 PTP4A1 FOXD1 NAA25 ARID4B ELL2 UBE2V2 BECN1 RNF157 STX16 UBE3C KLF10 CTHRC1 RFX7 VPS13C KPNA6 TRPS1 KDM3A ATP2A2 PGM3 FAM81A GATM TAOK1 E2F3 PHTF2 USP44 NOTCH1 PTPN13 VAV3 MIB1 STK17B RNF44 DPY19L1 ATP2B1 LMBR1 DNAJC13 ZNRF1 ZNF200 SLC35B4 GOLGA8B

Tarbase miRBD：99

CHORDC1 PAPOLA ATG2B TRIQK PHF20 DSCC1 ANLN ZNF529 HECTD2 ALDH2 TMEM87B NKTR TMEM170A ZNF354C TANK OSBPL3 PLIN3 NEFL NCK2 NOL4L MAST4 ABCA12 B4GALT4 PDK4 RGS1 ALS2 ZFX PNISR SIKE1 EXOC4 RMND5A URGCP RCHY1 SAMHD1 USP46 DMXL2 TTC8 PLEKHB2 SYNCRIP ZNF585A LRRC8B CLIP4 SLC35G2 ABHD10 SLC35C1 TM4SF1 SLC39A10 ERMN ATG12 BCL10 LYRM7 ZFY RRN3 PRTG CTNND2 ENPP4 KDELC2 ZBTB38 KIF11 RAB14 RCOR3 NCEH1 PPP2R1B VPS41 ZNF566 CNR1 ARPC5 SLC1A2 GOLGA8A GM2A DENND4A RNF168 ERI2 IQCB1 EEF1A1 VCPKMT CACNB4 NAP1L3 MGAT2 DENND1B PLAG1 SPIRE1 MICAL2 TNFRSF10B SCML1 ZNF100 HBS1L YBX3 ALG10 RRAGD TRMT10C TNFSF9 PEG10 MEX3C CAPN7 TFPI2 RANBP2 ZNF678 C15orf40

Targetscan miRBD：539

PKNOX2 C10orf25 RTKN2 NAGPA SLC7A6 ADAM12 EPB41L4B ERG USP47 HCN1 CCDC97 SLC16A14 OSBPL8 NR4A2 SNX33 RPS6KA5 SDAD1 ENOX2 GRK5 PARP16 A1CF KPNA3 CEP170 VAT1L ST8SIA4 GOLGA6L10 KIAA1211L GPR75-ASB3 C8orf44-SGK3 ASCC1 PIRT SLC6A6 TNXB TASP1 EDNRA IRS1 ACTC1 ATF1 PALM2 SLC35A5 RARB HEPHL1 NAALADL2 PPP1R12A DGKD KCNA4 SH3PXD2A GNAO1 GOT2 RAB38 SCN3A DCX RRAD PPARGC1A RALGPS1 NCOA3 SOX9 PIEZO2 SLC35F1 PITX1 VSTM4 ADRB1 HOXB8 ITSN1 CELF5 ERMAP PDSS1 GRHL1 ATP2B2 YPEL5 RIMBP2 POLR3G DLGAP1 PLEKHM3 SLC29A3 NF1 SLC25A34 NEGR1 FAM49A TEX2 PPID UBE2J1 CCNK TMEM229A DNMT3A NT5E ITGA6 EAF1 TOX PGGT1B GCNT2 MEX3D CALB2 HERC3 SDK2 MXRA5 PDE4D ARHGAP26 ERC2 RASGEF1A RIMS1 PIK3CD PPM1E VAT1 CHST2 ADRA1D FRMD4A GLCE SLC30A2 FAM104A SNX29 WWP1 GCLC SPAST RPS6KA2 BNC1 ZNF608 SLC22A5 ABCC9 VPS26B MTF2 CACNA1C DOC2A GRM3 TRPM7 KIAA0408 ANO4 SLC22A23 GTF2H1 C9orf72 ELFN2 MIER2 IRX4 FNIP2 SBF1 PTBP3 EPC2 STIM2 PCDH20 RASGEF1B UNC5C CYSLTR1 NECAP1 CSGALNACT1 SMAD1 SOGA3 SORCS3 ANKHD1 TENM3 WDR7 FKBP3 PDE5A KCNJ12 NRG3 ACVR1 ESCO1 PIK3R2 ABL1 EBF2 CALCR DIO2 MAP3K12 SEMA6D CMTM4 WDR44 GLDC PI4K2B FBXL17 RAB22A BAHD1 GAS2 POP1 IFNLR1 METAP2 TNIP1 SHOC2 NTNG1 GOLGA6L4 RUNX2 MBNL3 PCDH17 RFX6 NADK MAN1B1 ADAMTS3 MED12L NHS GLI2 NAV3 PAXBP1 LOX TAB3 ILDR2 KCTD8 NEUROD6 CAT UNC5D TBC1D15 HELZ FRMPD1 TET1 DMD RABGAP1L IDH1 SH3RF1 MICAL1 ATRNL1 MIA3 NSG1 NEURL1B WIPF1 CALD1 HOXA11 CEACAM1 IDE SUCLG2 SEC61A2 UCP3 ACTN1 RSF1 SEMA6B KCTD3 NKX2-2 DOCK7 LIN28A FBXO28 BNC2 NRK CTTNBP2NL PABPC1L2B ME1 ROR1 EPHB2 UGT8 SCAF4 MID2 CCDC148 MSANTD4 MAB21L1 TRMT5 AMER1 MAST3 YPEL2 SLC6A3 BEAN1 EYA2 RFX2 HECW1 ZNF652 ACTR3C B3GNT5 LPP HIVEP1 TNIK RCBTB1 SOS1 TAF4B TRIO TUBGCP3 POU4F2 HMGB3 ZBTB34 KLF11 GPR180 SOX13 IRF2BP2 CLRN1 ALPK3 PTPDC1 ATAD2B KIAA1522 AIDA ABL2 DIP2C STX2 TMEM86A GLUD2 ZNF197 EML1 TRDN UBE2F PLCG1 ATP8B2 ZRANB3 UBE2I LPGAT1 RAPH1 C14orf28 CHST1 CACHD1 HDAC5 SOCS6 LMLN WDR1 MARK1 BDNF CEP170B MBOAT1 SKP2 JAKMIP2 ARID3A ARF4 LRRC17 CCSER1 ZPBP2 GNG10 PAG1 NEFM ADAMTS9 TRAF3 ZMYND8 KIF16B NRIP1 TRO ZNF382 MAU2 HACE1 GALR1 ITGA8 ASB3 RUNX1 DBF4 MTTP KCTD5 PPTC7 HDAC9 WIPF3 ZCCHC24 IP6K3 ABCD2 AGO4 LEPR TENM1 STK38L C4orf19 CCDC120 KSR1 CBLB RAB27B TLL2 TBC1D2B STC1 LYST SOX12 ARL10 ST8SIA1 SH3KBP1 ARHGEF3 ATG5 DOLPP1 TTBK1 STXBP5 KXD1 PABPC1L2A EIF5A2 ADAM10 TSPAN33 LHX8 PDS5B ZNF644 TNKS LRFN2 DNAJC25-GNG10 CTH COL4A3BP SLC30A4 HTRA3 KLF12 RUNDC3B OSTM1 POLR3E CDCA7 USP45 USP2 FAM155A CYYR1 CCNJL TXNDC5 RALGDS TRIM9 COL25A1 EPC1 SLC7A10 SLC35F3 ELMOD2 CHKA MSANTD3-TMEFF1 PDCD10 COL13A1 CDC7 SNAPIN GPR26 HNRNPC RNF165 FXR1 ICK LHX1 GZF1 XPR1 ITGA4 CLOCK KATNBL1 DACH2 JAG2 FOXP4 CACNA1D PRKAA2 UHRF1BP1 PGP GLUD1 IL1RAPL2 ASXL3 P4HA2 EPB41 LATS2 BRAP PLXNA2 TVP23B STX17 BCL2L11 LYN AFAP1L2 SLC36A1 SNX27 PHF6 KLF8 RAP1B ELAVL4 TRIM13 TFDP1 SLC35D3 RAB3D CALU TDG LIMCH1 SSH1 SAP30 GRM5 CA10 CEP76 LGI1 ARAP2 NR5A2 SNX30 SIX4 NLGN1 JARID2 KIAA1549 JAM2 PCDH10 LRRC40 THBS2 GATA5 FRK WNK3 SMIM14 PROM1 PRLR AP3S1 ZFYVE26 KLF14 NFATC2 CAPN5 GOLGA6C TMEM121 SALL4 SAP30BP BAZ1A C7orf43 FAM83F ZSWIM5 CEP41 RAVER2 RANBP10 BACH1 ARHGEF6 SRGAP3 NCALD PHF13 SLC35A3 DLD SLC38A1 MMD KCTD7 FAM217B STAC SCN8A EFNA3 CAMKK2 PAX3 PSMD7 SSX2IP SIK3 SIX1 ITGB3 CARF VOPP1 PPP1R14C GPT2 ADAM22 MAP4K4 KIF21B GMNC PRICKLE1 TTPA PPP1R18 FAM131B PEX5L MFHAS1 YTHDC1 ATAD5 FAM110B PRRT2 FBXO32 ESPN CRKL AP4E1 SLC38A7 CLCF1 FGD6 ABHD6 GOLGA4 LHX9 MAP3K19 CYP24A1 RFTN2 SLC35F4 PITPNB MAT2A PAPOLB SCN9A GRIA2 DACT1 CAMK2N2 MDM4 ATRN RAB8A RNF169 APBA1

**Tarbase Targetscan：140**

VPS33A G3BP1 TPRG1L TET3 CLCC1 ZIC2 BCLAF1 PPIL4 POLE3 RNF217 MFSD11 TSPYL4 B3GALT5 FOXB1 PHF16 PRRG1 HLF AHNAK SEPT3 C7orf60 PRRX1 YWHAZ ANKRD52 FST LDLR DDI2 LPPR4 FOXN2 SURF4 ALG9 PAPD4 UBE2K MFAP3L PANK3 PIAS1 LPHN3 BBX NAP1L1 SEPT7 FAM73B KDM5B PPP2R4 SIAH2 SMAD2 YWHAG PUM2 GDNF BIRC6 TACC1 PDCL NLK TNPO1 PDP2 CTPS1 ELMO1 RALGPS2 RASGRP3 STAU1 CLCN3 CRMP1 TMEM41A CBX3 C16orf87 KIF3A CPOX MAF STXBP1 ELMSAN1 PYGO1 SFMBT1 KLF13 C11orf84 STX6 KIAA0101 TECPR1 HNRNPA1 RANBP9 MEF2D WASL MATR3 NRP1 CDH13 CLN8 TIMP3 FUBP3 CTDSPL2 GAN LRRC58 C9orf41 ARL4C DHX40 HSPA4L FAM208B YY1 SMARCA5 PLS1 PRKAR1A CD226 FAM168B STX7 SNX2 AQR CBX8 DENND5B STYX RIOK3 HIAT1 FAF2 PIN4 RPRD2 MLK4 PRKRIR MARCKS PAPD5 INHBA BMPR2 PCDH19 FAM46A HSPA5 H6PD MARCH6 NECAB1 IGF1R DCP2 POLR1D SIDT2 TRIM71 SCAMP1 JAK1 GPRIN3 DLGAP2 RLIM PVRL3 ZNF770 CEP44 UBE2D2 DHX36 PXK HIPK1 TMEM106B

**Supplementary material Text 5:**

The list of prediction of hsa-miR-501-3p target genes of venn diagrams.

**Tarbase Targetscan miRBD：21**

TET3 ANP32E CLDN11 CLIC4 STIM2 PAFAH1B1 NAP1L5 OTUD4 CBLL1 RCC2 RPRD1B ZBTB43 AFF4 ATXN1 LDHA HMGCS1 PEG10 SBNO1 ZC3H12C WDR82 SEMA3C

Tarbase Targetscan：9

MARCKS RPL15 DHX40 SH3PXD2A SETD7 TSPAN4 CSNK1A1 FJX1 EEA1

Tarbase miRBD：20

RCOR1 TBCC RC3H1 SEPSECS NAT8L XPO1 RASSF5 ZBTB10 MTR SSR3 SERTAD2 JADE1 RAPGEF2 TJP1 NR1D2 NRBF2 SEC24A C1orf131 BCOR SRSF3

Targetscan miRBD：77

KDM4A RNF38 AGFG1 RAP1A KPNA4 TFDP2 SYNE2 EXOC5 KIAA0408 CDYL2 CCDC50 CPEB2 SAMD12 MLLT6 KCNA1 FBN2 HOXD10 GAN SCN2A TSHZ3 EML6 CSDE1 DCLK1 PSMD11 RAI14 UBE2H ARIH1 KRAS MYCN MYNN RNF144A ZBTB20 DCUN1D5 LYSMD3 ESRRG FAM126B PHOX2B YY1 ZMYM4 ADAMTS3 SIM1 SEC63 RBM41 CDK6 ZFHX4 TMEM248 PPP4R2 UNC80 UBE2E2 ELAVL2 SNX13 ADCY2 ASPHD2 GRIA1 SHPRH ST8SIA2 BCL7A UCHL5 PPP2R2C SREK1 PSIP1 KLF12 PPP2R5E DCC ACTN1 LIN7C JDP2 DOK6 B4GALT5 SCN2B ONECUT2 NCOA4 KCTD9 GABPA SMIM13 USP37 RBMS1

Supplementary material Text 6:

The list of prediction of hsa-miR-103a-2-5p target genes of venn diagrams.

Tarbase Targetscan miRBD:73

CARD8 DDX18 EPHA4 STRIP1 ACLY KIAA0232 MTF2 MYSM1 SESN1 PURA RASA2 ARHGAP5 PDHX NFYB TLK2 RPN2 JPH1 STX6 RBAK KCNE4 NUFIP2 ATP6V0D1 PRKAR2A PAFAH1B1 SLC38A2 OXR1 DENND5B ZBTB39 MCL1 MTX3 BMPR2 TMCC1 ACVR2B PAK2 CCDC88A TRIM33 PAPOLA ZNF280C ABHD2 SON ZNF711 ASH1L ZNF805 BVES SNX13 EPHA7 KCNN3 BBX TNRC6B CCPG1 ARL6IP5 SYNCRIP IGF2R MKRN1 DDX47 LPGAT1 RNF180 ASF1B STIM1 CDK6 MAN2A1 LAMC1 SEL1L ROCK1 TBCA LIFR MGA TMEM245 SRSF1 BACH1 CCND2 EIF4G2 ARAF

Tarbase Targetscan：196

C5orf42 VPS4A KCNC1 WHSC1L1 EIF1 POLG BTF3 CHMP7 RSU1 CCT4 ENTPD7 CUL4B PAQR3 TMED7 FAM120A IMPAD1 HOXA5 FOXN2 SULF2 NFAT5 ZBTB47 ZNF18 KIF5C MAN1A2 RFK AP1S3 KMT2D MYO9A TXLNG TACC1 SERTAD4 TRIM4 MDM2 ATP5G3 TACSTD2 KLHL18 IER5 CIRH1A NADK ZCCHC2 SV2A HELZ OST4 DOCK7 GMEB1 NCR3LG1 ATAD2B TTF2 SLC35F6 LNPEP TMEM66 R3HDM4 C19orf25 DUSP2 EXOSC10 RNF144A SMC1A POGK IREB2 GRSF1 MAPK14 PRR14L SMARCA5 TCP11L1 SAMD8 SCD ROBO1 TBC1D20 RABGAP1 IPMK KPNA5 IRF4 ATP6V1B2 PLCG2 CAPZA1 SERINC5 GCC1 WBP5 SASS6 TRAPPC1 NUP160 HELLS PLAGL2 DCP2 EDEM1 UBA2 SLC38A1 MLXIPL ZNF326 STX16 PRICKLE1 WNK1 MEX3C STK38 ITPRIP APPL1 APP IGF2BP1 ARNT2 TLN1 CXADR PDIA3 DCP1A NOP9 CELF1 POU6F1 VTCN1 OCRL CBX6 ANKRD52 ZFP41 CEBPD OSBPL3 PTGS2 ATP6V1A ATXN7 IL21R FAM91A1 ATP2A3 NUP35 GFPT1 NUCKS1 RCOR1 YWHAG PPP3CB SFXN1 MAPK1 ATP9A HN1L HNRNPA2B1 USP46 MEX3A ACSBG1 METTL21B WDR77 SP2 RAX SHOC2 SSR1 RAB11FIP1 SEC11A MAP3K7 ATXN1 ELAVL1 USP5 ELMSAN1 XRCC5 CERS6 GNA12 LIMK1 CALM1 SLC16A1 ERC1 PVRL1 SEC23B SFXN2 PTOV1 PCYT1A EDC3 TAF2 DHX33 BNIP2 CCNG2 HSPA4L UHMK1 AGO4 SNAP25 SOX12 SPEN CBX8 TRERF1 GID8 CSNK1A1 ICK UBE2G2 PHACTR4 PPP1R12B MARCKS ACADSB DDRGK1 NFIB CHST9 TBPL1 SYS1 SLC18A2 IGDCC4 TRRAP MSH2 STK35 SPCS2 TTC17 LRRC14 FCHO2 HIPK1 ANXA11 LMBR1

Tarbase miRBD：2

NF1 RCOR3

Targetscan miRBD：618

APBB2 SLC10A7 SLC6A11 C12orf66 C2orf49 ERG KCNK3 MSRB3 ADAM7 MRPL1 RELT SLC8A1 GAGE1 CEP350 LGI2 ANKRD6 ARHGEF9 TNFRSF9 UFM1 FAM174A LCOR CCAR1 GCLM CPEB3 BCL2 ARL13B WDFY2 TAF12 NIPSNAP3B RSPH4A SRP72 FGF1 TFAP2B ZBTB20 GNB4 LONRF2 BST1 FAM221B GFI1 FHL1 LUZP6 SORBS2 MUC15 SH2D2A LMO4 OPA3 MICALL1 TBX21 KCNJ6 SLC46A2 LDLRAD2 APBA2 TCF21 DDI2 KLHL28 LAMTOR3 TACR2 LGR5 XIAP NEGR1 CD83 GK5 NOTCH2 ADD1 HSCB FLI1 TMOD2 MAB21L2 CLIC5 FAM122B PTPN4 SETBP1 CDH11 ZBTB41 KANK4 NRXN1 ARSA SUPT16H MCAT MAGI1 ERVV-1 ACER3 KLHDC1 PLXNA4 DEPDC1 PTCD2 FAM83A CCL23 BMF GRAMD1B XPO6 DUSP3 TUBB2B CTNNA1 CNTD1 SH3TC2 PADI4 SLC17A6 NDST3 TNPO1 FZD5 PCNP TMPRSS3 PALMD IPCEF1 C2orf80 SZT2 HCFC2 UBE2H PIWIL2 NCOA1 PDZD2 TARDBP GRIK3 CACNB2 PDE10A RHOQ MAP3K4 NABP1 PHEX RAP2A ABHD10 UBE2A IFNA14 SULF1 KIF3A LURAP1L ZMYM2 DAGLA MIS12 KLHL26 EXO1 C20orf194 AGMO OTC NMT1 SOCS5 TMIE TIMM50 BMI1 SH3RF1 ZHX1 BCL6B CHRM3 UST VIPAS39 ARHGAP24 C16orf70 VAMP7 CYB5A SLIT2 NXPE3 PDK3 LYRM7 TNPO2 SYNM KAZN CSNK2A1 AAK1 AKT3 TBC1D8B BAAT ZNF280D MEF2C ZNF485 EIF4EBP2 RYK TGFB2 MBNL2 TAF4B RAP1A FCRL6 GNAI3 C5orf24 MSN CDK15 ABL2 STAG2 ARHGEF12 PTGDR THAP10 GALNT13 RBM33 DPH3 TMEM30B FAS SPRN PRTFDC1 CYSLTR2 COPA ANAPC13 SLCO3A1 SUPT5H PAX9 TCHHL1 ITGA8 NDUFB4 DSC2 LRRC31 ISPD PLAGL1 KLF6 ABCD2 BHLHE22 LTBP2 LEF1 ZFHX4 AKAP10 ANK2 NAA50 SPR ANGEL1 LIN7A ARSB CPM PLD5 DPYS CDC42 KLF12 LRRC15 TMED5 MAP7D2 PLCD4 SRGN CASP7 RCAN3 AGXT2 H2AFZ AKAP17A CHODL TXNIP TRIP13 POLR2D TMEM154 FXR1 PSMA2 PRKAA2 ABCG2 PCDHB7 DSC3 EZH1 USP31 KCNH8 CERS5 OPN5 SOX5 INA BCORL1 TBC1D30 IFI44L ARAP2 NAV1 SHISA9 PPM1L UBE4B HIF1A TMPRSS11F KMT2A AFMID EIF2AK4 SUPT20H UBQLN1 NKAIN2 DNAJB5 TSPAN6 TSSK1B RASAL2 PHF20L1 PLCD3 SOWAHC TSR1 ZFHX3 FBXO45 ANK3 PTP4A1 CDH12 SNX24 TMEM132B CBX5 IFT57 PPFIA4 FEZ2 ZBTB5 WNT9B ATG16L1 EOGT POM121 TRIM25 GATM NDFIP1 DAPK1 VAV3 DAAM1 GOLT1B POU5F1B HAPLN1 CNOT6L KPNA7 G3BP1 IMP4 TMCC3 SYCP3 SLC4A8 PCSK1 MTMR1 FADS1 EPS8 ACSBG2 KCNA1 GOLGA3 CLEC4E EMP2 UBR3 STARD13 ALCAM FAM162B ZNF605 RHEB ADAM19 DAO EIF3M DEPDC5 KCNE3 RGL1 OXTR PALM2 CLSTN2 C1orf141 AHCYL1 FAM126B MEGF10 HEXIM1 SCN1A CSTA PROX1 MTHFD1 AMMECR1 NSD1 IFT43 MYOZ2 LRBA TBC1D8 GAB4 TRAPPC2 ZNF827 ZNF439 HRCT1 BARD1 VEZT FCAR COL19A1 NET1 IPO5 ESR1 ANAPC1 PTPN14 FAM53A NFATC3 PDE3A IL36G PITPNM2 DERL2 FIGN ARID2 GNA13 TSPAN31 LRRC39 FAM217A EPHB1 DLL4 MYOCD PTGER4 ZBTB24 RGS1 ABCC9 HDDC2 FAM111A BLNK CHGB GLO1 SEC16A SMAD2 EFNA5 NOL4 METTL14 RALGAPB CCDC125 C5orf63 TBCEL NGEF GDNF PGF G3BP2 NHLRC2 POSTN GTF2A2 LILRB3 MS4A4A RPGRIP1L SLC22A7 ADAMTS15 KRTAP2-1 POLR2F TSC22D2 POLDIP2 RAB3GAP2 SOGA3 VPS37A CPSF6 C1QTNF6 BLCAP HMG20A MAPK8 PHF2 PID1 SYTL2 SMPD3 DNAJC21 CALN1 SEMA6D ZCCHC17 LYSMD3 HIST1H2BK TMEM200B SGCD TMEM108 GPR85 FFAR2 ENTPD1 SMG7 KIAA1211 BAG4 UBE2D4 TRAK1 USP12 RABGAP1L DLC1 MAPK8IP3 RAP2C POU5F1 NFXL1 GFRA1 COMMD3-BMI1 ACTA1 ECT2L SP8 LARP4 SOBP RSPO3 DNAL1 BEND3 CNNM2 SBNO1 SEC24A LRAT FSD1L C2orf40 PBX2 ZC3H12B ADARB2 NUDT21 ZNF440 STT3B TMTC2 COBLL1 MICU3 SOS1 AFF1 GTF2A1 SLC28A3 GPR180 SYNPO2 CYLC2 LYRM2 GPR3 RC3H1 TAF7 VEPH1 AIDA SLC23A2 PAIP2 DACH1 APOL6 DICER1 ZNF148 IKZF1 XIRP1 GAN MRGBP CDKN1B USP49 DTX3L MRPS11 CADM2 LRRC17 MAGI3 HAUS6 NRP2 CDH2 ATP7A IFT80 DLL1 C11orf58 NRAS OGG1 RAB21 C12orf29 PBRM1 NR3C1 NPAP1 USP13 NDUFA4L2 TENM1 NME6 ZNF236 AFF4 LYST SNX4 RIMS3 ASIC1 CLVS2 NSL1 SLC44A1 LIPA GALM SCAI TECPR2 RBM46 CDHR3 CD46 NEDD1 LRRC10B LONP2 RAB31 DROSHA HEG1 CLASP2 ZBTB8A GPR26 HMGA2 MTPAP HHAT TRAPPC13 CLOCK RILPL2 GPR82 WBP1L KCNK2 VEZF1 DNM3 LSM11 DPF3 EPB41 CLEC3A IKZF2 SOCS7 CRX ATP1A2 BCL2L11 SCPEP1 ZFP91 GPR137C PLCB1 KLF8 PLXDC2 SBF2 PCF11 ZBTB33 SPTLC2 SCN2A ALDH8A1 ASIC2 RALA ZNF449 APOBEC3H TET2 ELOVL6 MICAL2 PRKD1 CXCL9 SIX4 SLC20A2 ZDHHC23 TRIM32 DDC FAT3 CYLD METTL7A SERTM1 ZNF180 SRSF2 FAM84A ROBO2 ZMYND11 GXYLT1 DYRK1A NEDD9 MYRIP RGS10 PEX11A NPTX1 SLC34A2 SCN8A TJP2 SLC9A8 DUSP15 SEMA5A RBM27 AGAP1 CLIC2 ONECUT2 LGR4 CLDN18 TADA2B ANO6 PRKCA C17orf47 ANKRD23

**Supplementary material Text 7:**

The list of prediction of hsa-miR-181d-5p target genes of venn diagrams.

**Tarbase Targetscan miRBD:191**

GSKIP WASF1 DDX5 MAP3K3 OSBPL8 ZIC2 LRRC8D ST8SIA4 LCOR BCL2 CDK17 LIF INO80D SERTAD2 CCDC6 LIN28B IL1A ELAVL2 TANC2 UBE2D3 NOTCH2 ZNF136 ANP32A IRS2 SLC7A11 MME C2orf69 TXNDC12 FIGN E2F7 ZFAND6 ZBTB4 AVL9 GLCCI1 PNISR MTF2 ADARB1 ZNF664 AP1S3 PAM ZFP62 GFPT1 NUCKS1 FBXO34 YWHAG RMND5A SRSF7 CBX4 GHITM G3BP2 CHD7 BIRC6 EPC2 CCNJ DMXL2 KLHL15 ZDHHC3 TNPO1 ZFP36L1 CREB1 DDX3Y ABHD13 TBL1XR1 MAPK8 ZNF426 CPEB4 RBBP7 ANKRD44 USP33 PPP1CB MPP5 BHLHE40 TARDBP MTPN XRN1 SLC25A36 NAB1 TMEM64 TM9SF3 ZNF594 PURB C16orf87 NCOA2 KLHL5 ELMSAN1 WDR37 MED26 AFTPH DEK SBNO1 LPCAT1 GIGYF1 GATC KMT2C BRWD1 SLC19A2 IQGAP2 RLF HECA WASL TMEM127 YOD1 ATP8B2 PGRMC2 C14orf28 ATP1B1 MARK1 SLC2A3 GPCPD1 B4GALT1 DYNC1LI2 FAM135A NACC2 KRAS ENAH ZFP36L2 PIK3R3 PIK3C2A NEK7 AGO4 RORB PLEKHA3 COPS2 CALR NAA50 SCD CD2AP CRIM1 ABI3BP NR6A1 CRYBG3 RIMKLB E2F5 UBE2B JAZF1 BRD1 KIF3B MTMR9 RAN YWHAB C2CD5 PRKCE SLC25A25 BTBD3 EPB41 PPARA RORA PNRC2 DDIT4 BCL2L11 RAP1B SLC16A6 ZNF699 MGA S1PR1 CEP97 MBNL1 SRPK2 ZNF302 FOS HSPA5 ETS1 HSP90B1 KMT2A CTDSPL ACVR2B SERPINE1 MAP1B AKAP5 CREBRF TGFBR2 MLEC TCF7L2 ZNF800 EIF4A2 FOXP1 ARF3 SSX2IP BLOC1S6 UBE3C ETV6 ADAMTS5 ONECUT2 DDX3X TAOK1 TMF1 TMED4 PITPNB ATP2B1 PUM1 ARNT2

**Tarbase miRBD: 54**

ZNF83 PPP1R9A CDCA4 CEP120 H3F3B ARL2BP DIDO1 ZNF568 ZNF439 MKLN1 WSB1 AHCTF1 GTSE1 KIF1B C8orf59 NAP1L1 OGFRL1 PNMA2 SNX5 NUDT19 ZNF544 ZNF121 TMEM123 ADM LONRF1 ACSL4 ZNF850 ZNF440 ZNF780A PRTG ZNF189 TXNDC15 RAB3GAP1 N4BP2 VBP1 TMEM9B ZNF124 ZNF217 ZNF101 DENND5B SOAT1 ZMYM1 C1orf109 IKBIP LIFR ZNF773 LATS1 HK2 ZNF14 CD48 ZNF700 SCAMP2 TLL1 RNF19A

**Tarbase Targetscan:69**

FSTL1 SLMAP IGF2BP3 CACNA2D1 EPT1 CNOT1 FOXB1 UBN2 AHNAK C6orf62 HNRNPA0 RBM26 USP9Y GCNT2 DERL2 BEND4 SSBP2 GREB1L ZNF33B CDC27 HNRNPH1 ADRBK1 TMEM181 ZNF772 PPP6C SMG7 PDIA6 RPN2 RSF1 DNAJB14 CYR61 RBAK RCN2 PSD3 CKAP4 KLF3 WWC2 KMT2E AK3 RASL10B ATP11A DYRK2 YY1 SMAD7 KIAA1244 STAT3 MADD DCAF16 MCL1 HMGB2 ZCCHC14 PKN2 OGT LRP6 ADAMTS1 MAP3K2 SLC9A6 ARRDC3 BAZ2A MARCH6 CCNG1 IL6ST PPAP2B GAS7 NPTN RBM27 COL5A1 ZNF200 ZNF788

**Targetscan miRBD:635**

PKNOX2 INPP5E KLHL2 SLC10A7 MITF HIC2 TAF9B CLUH DCLK3 RAB3IP SPECC1L UNC5A PCSK1 HOXA1 LRRN1 ARF6 CHD1 PCDHAC1 CMPK2 ITGA3 BMP3 LUZP1 CAND1 CECR2 ZFP14 PDCD4 BCLAF1 RAD23B PCDHA13 EXOSC2 EVI2A DMTN DNAJC5 GATA6 CDK8 RIN2 CCAR1 RNF217 TRIM2 HYOU1 TUBB GCC2 OTUD4 C8orf44-SGK3 RFX3 CHMP1B PLCL2 MLF1 DUSP5 ERLIN2 ATP11C THRB USP42 CDC42BPA NIPBL GRIK2 IGSF11 PHLDA1 SLITRK4 KDM5A TMEM87B QKI PALM2 EPHA4 ZNF655 CDH8 KCNQ5 HLF HEPHL1 IGDCC3 GNB4 NR2C2 NAALADL2 FRYL FAM126B RSPO2 KCNA4 NMNAT2 ACVR2A PGM2L1 YWHAZ ANKRD52 SCHIP1 RPS6KA6 MAGOHB SNN ZC3H6 GOT2 NIPAL4 PROX1 KCNK10 RNF182 PDAP1 KCTD10 AKIRIN1 FHDC1 ITSN1 TNF SLA ATP2B2 PAWR MTMR12 SIX2 CPD RABGEF1 LRBA SPAG9 OSBPL3 TM9SF4 SPP1 MIP NEGR1 YTHDC2 MAP1A ATXN7 ZNF781 TGIF2 CCNK GAD2 ITGA6 PDCD6IP IPO5 ESR1 KCNN3 CCNDBP1 NFAT5 WIF1 PLCXD3 MELK CUL3 PODXL FAM160A2 GRM1 ZNF514 PCDHA12 NR1D2 ITGB8 PDE3A PTPN4 HIPK3 EPB41L3 SOCS4 MFAP3L ACAN ZBTB41 DOCK4 ZNF780B ZIC3 FHAD1 NRXN1 NAA15 NR4A3 EVX1 PCDHA7 ZFP1 ASAH2B GPD1L DUSP10 CNKSR2 WDR82 MAPK1IP1L PIAS1 LRRFIP1 CNOT2 GABRA1 METAP1 HMBS SLC25A24 SMAP1 GPBP1 ACER3 TNRC6B CACNA2D2 MYO1E KCNH1 TBC1D24 MYBL1 FAM3C KPNA4 FLT1 RCOR1 MKNK2 LRRC32 IGF2BP2 SLC2A14 PCDHA5 RALGAPB FMR1 C6orf89 CCDC117 USP15 ZDHHC7 ENTPD6 VKORC1L1 TBCEL FAM102A MAPK1 FNIP2 LCLAT1 GPSM1 SAMHD1 PTBP3 PPP3R1 STIM2 CDON MMP14 NLK SPTY2D1 HOXB4 IPPK NBEA FAM19A2 ZNF268 CLASP1 AFAP1 PAPOLG TTC39A PCNP TSC22D2 IQCJ-SCHIP1 BAZ2B CPSF6 EYA3 TTC39B SLAIN2 GLIS3 TCERG1 SLC35E1 PDE5A CBX7 F2R PHF2 APOO CAMSAP2 SRGAP1 CALCR DNAJC21 MBTPS2 PI4K2B FBXL17 MCC STAU1 GRIK3 METAP2 PAN3 PDXDC1 SOX6 CAMTA2 TRPM3 MB21D2 SMCO1 VCAN ZDHHC21 MED12L CHIC1 ACAP2 LOX UNC80 KIF3A TAB3 BAG4 PPIP5K2 UNC5D ATXN1 CPOX PAK4 KLHL42 RASSF8 PDGFRA NOVA1 SPRY4 KCNC2 SOWAHA CHURC1 FNDC3B ASTN1 SIRT1 NSG1 NEURL1B ECT2L RNF8 HOXC8 LARP4 HOXA11 SFMBT1 DNAL1 PSPC1 LMO1 GSE1 BEND3 CCP110 POU2F1 DOCK7 LIN28A SGK3 INO80 ADAM11 CTTNBP2NL BACH2 FSD1L CALM1 FAM13B ADAMTSL1 MID2 PAX5 AAK1 AKT3 ARL5A ADARB2 USP8 XPO7 ARSJ KAT2B MAP3K9 PCDHA6 MAEA AP1AR ZADH2 CBFA2T3 CDYL MLXIP ESM1 AMER2 KANK1 LPP TNIK ADAMTS18 PLAU MBNL2 PTEN AFF1 TBC1D14 AGFG1 MEX3B SLC25A37 HLTF AP1G1 ZBTB34 CD4 PTPRE MFSD6 RAB3C RAB8B PHLPP2 MECP2 DIP2C LYRM1 TIMP3 ZNF704 SCOC CARD11 YIPF4 MPP7 SLC16A7 PHF3 DCBLD2 PRKAR2A YTHDF3 PAFAH1B1 PCDHA4 CNTN4 CTIF DNAJA4 MINK1 AFG3L2 CNKSR3 MIER3 IPO8 PHC3 RNF34 TNFRSF11B SECISBP2 PAX9 DCLK1 MEF2A BCL9 MED8 FGD4 RNF145 GDI1 SIPA1L2 CDX2 PRDM4 PRKCD PLXNC1 ISPD HDAC9 DNAJC3 TMEM165 KIAA1549L CNR1 CPNE2 PHOX2B PCDHA2 FBXO33 PCDHA11 GOLGA1 ZBTB43 USP9X KLF6 NR3C1 PROSER1 HECW2 PEAK1 RALGAPA2 CBLB CAMKK1 ZFHX4 POC1B-GALNT4 ABTB2 SNAP25 TMEM47 STC1 LMBRD2 PLA2G4A PPP1R3B LPCAT2 PCDHA9 ARHGEF3 ASIC1 TTBK1 PDPK1 SRGAP2 GID4 CRISPLD1 STXBP5 ATXN3 ADO SLC26A4 DRAM1 TGFBR1 ACVR1C TULP4 MEGF9 ZBTB7A ATM PRRC2C TUB PCDHAC2 IPMK MAP3K10 MAN2A1 CLMN TNS1 MTMR10 KCNJ10 NMT2 ZNRF2 CREBZF ILF3 SLC35F3 LEMD3 HOXD1 POLQ ZFP82 ATL3 EGR3 MSANTD3-TMEFF1 KPNA1 PDIK1L PBX3 GALNT4 PRICKLE2 FOXK1 MTX3 SEL1L SLC4A10 TNFAIP1 CLOCK KATNBL1 LGALSL L1CAM PHACTR4 GAPVD1 KIAA1324L TBC1D4 PPP1R2 GRB10 PCDHA10 TBL1X PHIP ASXL3 CARM1 DLG2 BRAP SESN3 PPP1R12B NUS1 ZNF597 PSAP DUSP6 KLF7 GPR137C MGAT2 BPTF NPEPPS CUL5 SENP2 PRKG1 ZNF283 ELAVL4 NFIB DIP2B NLN GUCY1A2 STXBP6 SEC24C SRSF10 HCN2 ANKRD13C DCUN1D1 TBPL1 ANKFY1 PLAG1 TBC1D1 MYCBP2 LIMCH1 CCL8 RALA ZNF449 GNAQ DOCK10 RNF144B GRM5 FNDC3A SPIRE1 ST8SIA3 SACM1L RASSF2 CAPRIN2 ATP8A1 ANKRD50 RECK SIX4 RBM47 JARID2 MSI2 PAFAH1B2 TMEM131 CNTNAP2 SH2B3 TRIM71 SLC26A9 SEMA4G PRLR CDC73 NFATC2 ZEB2 WSCD2 PCDHA3 SLC12A5 ARL3 GABRA4 SALL4 KLHL29 OTOGL SYNPR RAB30 CLVS1 YLPM1 DEPTOR LMO3 ZNF470 NKAIN2 ZMYND11 NCALD SLITRK1 SLC35A3 PPFIA1 NOG KLF15 PCDHA1 PTBP2 HEY2 GPD2 VANGL1 ACSL6 QSER1 TOR1AIP2 HIPK2 PCDHA8 TMEFF1 SIK3 RAD21 MAP2K1 SS18L1 CLIP1 UBP1 TMEM132B RLIM KITLG PPP2R5E DERL1 MAP4K4 ZNF266 CDC5L KPNB1 ETNK1 CD69 WNK1 NKX3-2 FMNL2 MAMDC2 B3GALT1 ATP2A2 KRBOX4 TBC1D9 STRN SNAI2 TRNP1 PHTF2 TADA2B ADCY9 FAM160A1 GALNT16 FKBP1A RFTN2 INPP5A ZNF562 HIPK1 ATP8B1 RASSF1 MBOAT2 SIN3B TMEM106B SEMA4C ATMIN NPTXR VHL PARM1 RPS6KA3 DNAJC13 RPS6KB1 RNF169 APBA1
